# Optimal serum potassium concentrations in heart failure: an individual patient data meta-analysis

**DOI:** 10.1093/eurheartj/ehag341

**Published:** 2026-05-28

**Authors:** Ryohei Ono, Misato Chimura, Kieran F Docherty, Alasdair D Henderson, Ross Campbell, Akshay S Desai, Michel Komajda, Milton Packer, Marc A Pfeffer, Bertram Pitt, John R Teerlink, Faiez Zannad, Muthiah Vaduganathan, Orly Vardeny, Mingming Yang, Pardeep S Jhund, Scott D Solomon, John J V McMurray

**Affiliations:** British Heart Foundation Cardiovascular Research Centre, University of Glasgow, 126 University Place, Glasgow G12 8TA, UK; British Heart Foundation Cardiovascular Research Centre, University of Glasgow, 126 University Place, Glasgow G12 8TA, UK; Department of Cardiovascular Medicine, Osaka University Graduate School of Medicine, Suita, Japan; British Heart Foundation Cardiovascular Research Centre, University of Glasgow, 126 University Place, Glasgow G12 8TA, UK; British Heart Foundation Cardiovascular Research Centre, University of Glasgow, 126 University Place, Glasgow G12 8TA, UK; British Heart Foundation Cardiovascular Research Centre, University of Glasgow, 126 University Place, Glasgow G12 8TA, UK; Division of Cardiovascular Medicine, Brigham and Women’s Hospital and Harvard Medical School, Boston, MA, USA; Department of Cardiology, Hospital Saint Joseph, Paris, France; Baylor Scott & White Health, Dallas, TX, USA; Division of Cardiovascular Medicine, Brigham and Women’s Hospital and Harvard Medical School, Boston, MA, USA; Department of Internal Medicine-Cardiology, University of Michigan School of Medicine, Ann Arbor, MI, USA; Section of Cardiology, SanFrancisco Veterans Affairs Medical Center and School of Medicine, University of California San Francisco, San Francisco, CA, USA; Inserm Clinical Investigation Centre, CHU, Université de Lorraine, Nancy, France; Division of Cardiovascular Medicine, Brigham and Women’s Hospital and Harvard Medical School, Boston, MA, USA; Department of Medicine, University of Minnesota, Minneapolis, MN, USA; British Heart Foundation Cardiovascular Research Centre, University of Glasgow, 126 University Place, Glasgow G12 8TA, UK; British Heart Foundation Cardiovascular Research Centre, University of Glasgow, 126 University Place, Glasgow G12 8TA, UK; Division of Cardiovascular Medicine, Brigham and Women’s Hospital and Harvard Medical School, Boston, MA, USA; British Heart Foundation Cardiovascular Research Centre, University of Glasgow, 126 University Place, Glasgow G12 8TA, UK

**Keywords:** Clinical outcomes, Heart failure with reduced ejection fraction, Heart failure with preserved ejection fraction, Potassium, Prognosis

## Abstract

**Background and Aims:**

It remains unclear what the safe serum potassium range is in heart failure (HF) and whether it is the same in HF with reduced ejection fraction (HFrEF) and HF with preserved ejection fraction (HFpEF).

**Methods:**

A patient-level pooled analysis from 12 randomized controlled trials including 32 346 HFrEF and 13 723 HFpEF patients was performed. Baseline serum potassium level was categorized into six groups (<3.5, ≥3.5–<4.0, ≥4.0–<4.5, ≥4.5–<5.0, ≥5.0–<5.5, and ≥5.5 mmol/L) and serum potassium level at baseline was also analysed as a continuous variable using restricted cubic splines. The primary outcome was all-cause mortality. Secondary outcomes included cardiovascular death, sudden death, pump failure death, first HF hospitalization, and composites of HF hospitalization and cardiovascular or all-cause death.

**Results:**

The median follow-up was 24.2 and 36.8 months in HFrEF and HFpEF trials, respectively. In HFrEF, serum potassium levels showed a reverse J-shaped association with outcomes. Compared with ≥4.0–4.5 mmol/L (reference), potassium <3.5 mmol/L was associated with higher risks of all-cause mortality (adjusted hazard ratio 1.49; 95% confidence interval, 1.27–1.76), as well as cardiovascular, sudden, and pump failure death. The lowest risk for all outcomes was observed within the baseline serum potassium range of 4.2–5.0 mmol/L, but even ‘mild hyperkalaemia’ (5.0–5.5 mmol/L) was not associated with worse outcomes in HFrEF. Although the risk curve was U-shaped and flatter in HFpEF, the lowest incidence of all outcomes was observed over the same potassium range as HFrEF.

**Conclusion:**

In HFrEF, hypokalaemia is strongly associated with worse outcomes and should be avoided. In terms of safety, the optimal serum potassium concentration in both HFrEF and HFpEF appears to be in the range 4.2–5.0 mmol/L.

## Structured Graphical Abstract

**Structured Graphical Abstract:**
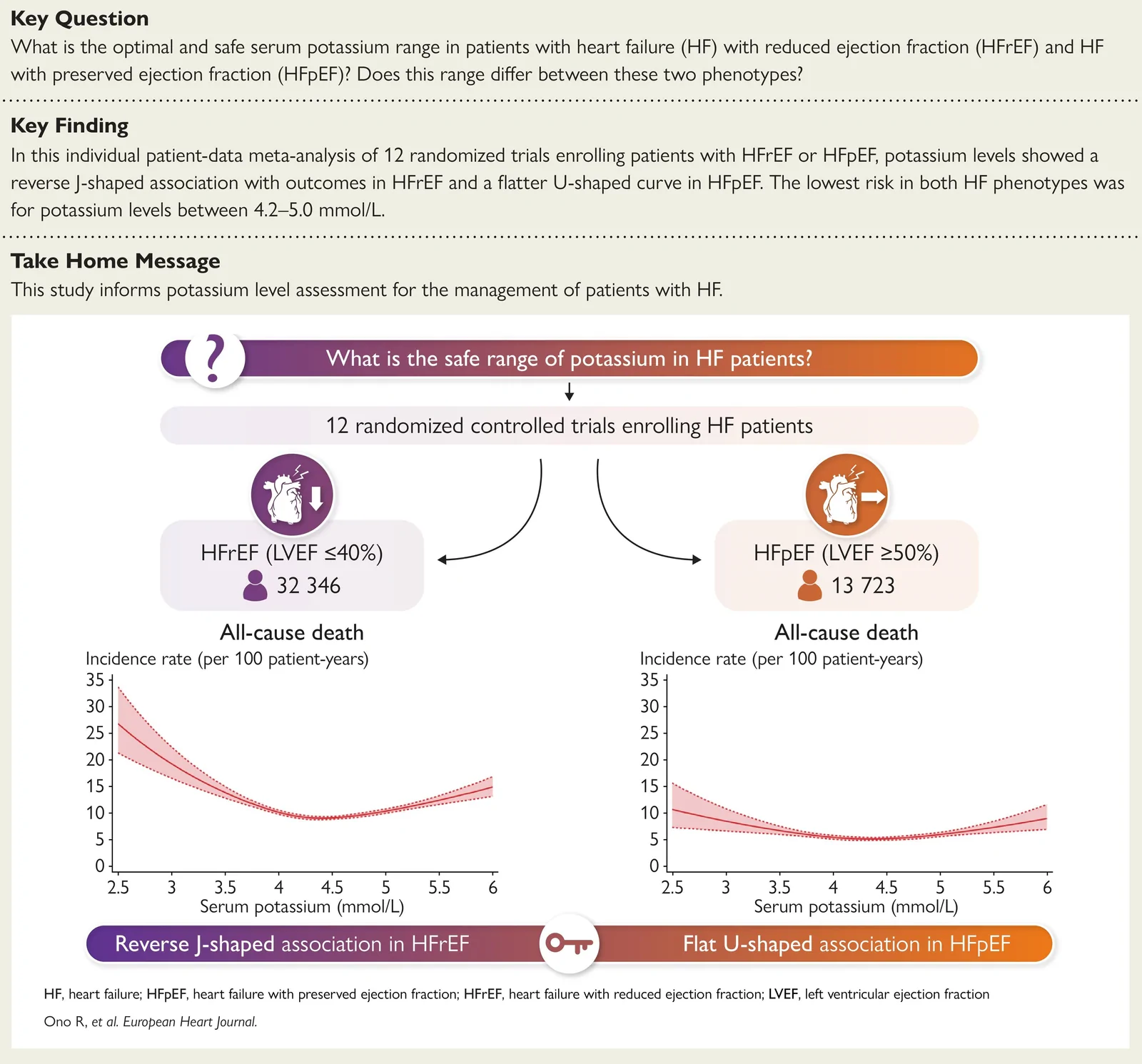
Association between serum potassium and clinical outcomes in heart failure with reduced and preserved ejection fraction. HF, heart failure; HFpEF, heart failure with preserved ejection fraction; HFrEF, heart failure with reduced ejection fraction; LVEF, left ventricular ejection fraction

## Introduction

Potassium plays an essential role in cellular function, particularly in neural and muscular tissue, including cardiomyocytes, where it is crucial for maintaining resting membrane potential and electrical stability.^1^ However, potassium homeostasis is often disrupted in patients with heart failure (HF) because of neurohormonal activation, medications (e.g. diuretics and mineralocorticoid receptor antagonists), and comorbidities (e.g. kidney dysfunction).^1–5^ Although there are many studies of the association between potassium concentrations and outcomes in HF, most have examined a single left ventricular ejection fraction (LVEF) phenotype or HF overall, without any stratification by LVEF.^3,6^ Second, most prior investigations have typically examined risk related to potassium using categories thought to reflect hypokalaemia, normokalaemia, and hyperkalaemia. However, it is not clear whether the potassium cut-points used to define these conventional categories are the thresholds that best reflect the association between potassium levels and outcomes in HF and recently interventions to increase potassium levels have been shown to improve cardiovascular outcomes.^7^ It is also not known whether the pattern of association between potassium levels and risk is the same in patients with HF and reduced ejection fraction (HFrEF, LVEF ≤40%) and those with HF and preserved ejection fraction (HFpEF, LVEF ≥50%).^4,6,8–12^ To address these critical knowledge gaps, we conducted a comprehensive analysis using individual participant-level data from 12 major randomized controlled trials, comprising 7 trials in HFrEF and 5 trials in HFpEF.^13–24^ By stratifying the population according to HF phenotype and examining a wide spectrum of baseline potassium levels, the aims of this study were to determine whether the relationship between serum potassium levels and clinical outcomes differs between HFrEF and HFpEF, and to identify the potassium range associated with the lowest risk in each subgroup.

## Methods

Individual participant-level data from seven trials of HFrEF (CHARM [Candesartan in Heart Failure Assessment of Reduction in Mortality and Morbidity]-Added, CHARM-Alternative, EMPHASIS-HF [Eplerenone in Mild Patients Hospitalization and Survival Study in Heart Failure], ATMOSPHERE [Aliskiren Trial to Minimize Outcomes in Patients with Heart Failure], PARADIGM-HF [Prospective Comparison of ARNI With ACEI to Determine Impact on Global Mortality and Morbidity in Heart Failure], DAPA-HF [Dapagliflozin and Prevention of Adverse Outcomes in Heart Failure], and GALACTIC-HF [Global Approach to Lowering Adverse Cardiac Outcomes Through Improving Contractility in Heart Failure]) and five trials of HFpEF (CHARM-Preserved, I-PRESERVE [Irbesartan in Heart Failure with Preserved Systolic Function], TOPCAT [Treatment of Preserved Cardiac Function Heart Failure With an Aldosterone Antagonist] Americas, PARAGON-HF [Prospective Comparison of Angiotensin Receptor Neprilysin Inhibitor With Angiotensin Receptor Blockers Global Outcomes in HF With Preserved Ejection Fraction], and FINEARTS-HF [Finerenone Trial to Investigate Efficacy and Safety Superior to Placebo in Patients with Heart Failure]) were included in the present study.^13–24^ The design, baseline characteristics, and results of these trials have been published in detail previously.^13–38^ Given the considerable regional heterogeneity in the conduct of the TOPCAT trial, we restricted the analysis to the TOPCAT America in this study.^39^ A summary of the designs is provided in Supplementary data online, *Table S1*. Each trial was approved by local ethics committees, and written informed consent was obtained from each patient. Serum rather than plasma potassium concentration was measured in all trials (serum potassium levels are slightly higher by approximately 0.1 to 0.4 mmol/L than plasma potassium levels).^40^ For each included study, patients from both the intervention and control arms were included in the present analysis. Exclusion criteria were a lack of baseline serum potassium level, potassium level >8.0 mmol/L considering the possibility of haemolysis, LVEF >40% or unknown in HFrEF trials, and LVEF <50% or unknown in HFpEF trials (see Supplementary data online, *Figure S1*).

### Trial outcomes

The primary outcome of each trial is summarized in Supplementary data online, *Table S1*. The primary endpoints of each trial were as follows: In CHARM, EMPHASIS-HF, ATMOSPHERE, PARADIGM-HF, and PARAGON-HF, the primary endpoint was a composite of cardiovascular death or HF hospitalization (total events in PARAGON-HF and time-to-first event in the others). In TOPCAT, the primary endpoint was the same composite plus resuscitated sudden death (time-to-first). The primary composite outcome in I-PRESERVE was all-cause death or cardiovascular hospitalization (time-to-first). In DAPA-HF, the primary endpoint was a composite of worsening HF (hospitalization or urgent visit resulting in intravenous therapy) or cardiovascular death. The primary composite outcome in GALACTIC-HF was the composite of HF events or cardiovascular death (time-to-first). In FINEARTS-HF, the primary outcome was total worsening HF events and death from cardiovascular causes. All-cause death was a secondary endpoint in all trials except ATMOSPHERE, in which it was an exploratory endpoint. In the present study, we used all-cause death, the most objective and arguably the most important outcome, as the primary endpoint. We also separately analysed non-cardiovascular death and cardiovascular death, including the two major types of cardiovascular death—sudden death and pump failure death—as well as first HF hospitalization, the composite of first HF hospitalization or all-cause death, and the composite of first HF hospitalization or cardiovascular death. Each event and cause of death was adjudicated by a central endpoint committee according to the prespecified criteria in each trial.

### Statistical analysis

The numbers of missing data in each trial are shown in Supplementary data online, *Table S2*. We divided the patients into two categories: HFrEF (LVEF ≤40%) and HFpEF (LVEF ≥50%). The following analyses were performed separately for patients with HFrEF and those with HFpEF. Based on serum potassium measurements at randomization in each study (only EMPHASIS-HF used at visit 1), patients were classified into the following potassium categories: <3.5 mmol/L, ≥3.5–<4.0 mmol/L, ≥4.0–<4.5 mmol/L, ≥4.5–<5.0 mmol/L, ≥5.0–<5.5 mmol/L, and ≥5.5 mmol/L. Baseline patient characteristics were reported as median (interquartile range) or mean ± standard deviation for continuous variables and as counts and percentages for categorical variables. According to potassium categories, continuous variables were compared by the Jonckheere–Terpstra trend test, and binary variables were compared by the Cochran–Armitage trend test.

The occurrence of each endpoint according to the potassium level was compared with Kaplan–Meier curves and the log-rank test. The incidence rates for each outcome were estimated per 100 patient-years. The associations between the potassium level, as a continuous variable, and the hazard ratios (HRs) of each major clinical outcome were modelled using restricted cubic splines with median potassium level as reference. The adjusted variables are listed below. The three knots (at 10th, 50th, and 90th percentiles) were placed at default positions according to the percentile of potassium level. In addition, the incidence rate for each individual and composite time-to-first outcome was evaluated across the spectrum of potassium level using a Poisson regression model, in which potassium level was examined using restricted cubic splines employing 4 knots. *P* for interaction was assessed using a likelihood ratio test, comparing models with and without the relevant interaction terms for potassium with baseline estimated glomerular filtration rate (eGFR) category (<60 vs ≥60 mL/min/1.73 m^2^) randomized treatment assignment, and use of mineralocorticoid receptor antagonists (MRAs). To evaluate the lowest risk of clinical outcomes, three approaches were employed: binary recursive partitioning,^41^ identification of the nadir of the HR, and the nadir of the incidence rate. Binary recursive partitioning was used to determine thresholds of clinical outcome risk, without prespecifying cut-off values, resulting in risk strata where the lowest relative hazard was observed. The nadir of HR was defined as the potassium level at which the spline-predicted adjusted HR was lowest, identified by calculating the minimum among the model-based predicted values. The nadir based on the spline-predicted incidence rate was also determined using the same approach to HR.

For time-to-first-event analysis, Cox proportional hazards models were used to compute HRs and 95% confidence interval (CI) according to potassium categories. In the Cox proportional hazard models, unadjusted HRs were stratified according to the trial and with the treatment assignment as a covariate. Adjusted HRs (aHRs) were further adjusted for age, sex, region, race, heart rate, New York Heart Association (NYHA) functional classes III or IV, systolic blood pressure, body mass index, LVEF, serum creatinine, haemoglobin, N-terminal pro-B-type natriuretic peptide (NT-proBNP, log-transformed), history of atrial fibrillation, history of myocardial infarction, history of diabetes, diuretic use, MRA use, and angiotensin-converting enzyme inhibitor/angiotensin receptor blocker/angiotensin receptor–neprilysin inhibitor use. Furthermore, additional analysis was made for the adjustment of the above variables and dose of oral loop diuretics, limited to more recent trials in which loop diuretic dose information was reported in a sufficiently detailed and standardized manner, including GALACTIC-HF, DAPA-HF, and PARADIGM-HF for HFrEF trials, and FINEARTS-HF and PARAGON-HF for HFpEF trials (see Supplementary data online, *Table S3*). For the dose of oral loop diuretics, total daily furosemide dose equivalents were calculated from medication records in each of the above trials. Bumetanide 1 mg, torsemide 20 mg, azosemide 60 mg, and ethacrynic acid 100 mg were considered equivalent to 80 mg of oral furosemide. In this calculation using the record, we only focused on: (1) oral diuretic use, (2) dosage units expressed in milligrams, (3) interpretable frequency of administration, and (4) exclusion of cases with total daily oral diuretic doses exceeding 1000 mg. In the cases with missing data, the missing indicator method was used for the NT-proBNP value and the dose of oral loop diuretics. In a sensitivity analysis, we conducted a two-stage meta-analysis in a random effects model for patients with HFrEF. We selected the potassium range of 4.0 to <4.5 mmol/L as the reference group, as it lies within both commonly used definitions of the normal range (3.5–5.0 and 4.0–5.0 mmol/L) in the previous studies and represents the midpoint or overlapping region of these prior categorizations. All statistical analyses were conducted using STATA version 18 (StataCorp LLC; College Station, TX, USA), and a *P*-value of < .05 was considered statistically significant.

## Results

Overall, 32 346 patients from HFrEF trials and 13 723 patients from HFpEF trials were analysed in this study (see Supplementary data online, *Figure S1*). The distributions of serum potassium concentrations are shown in Supplementary data online, *Figure S2*. The 95% interval of serum potassium concentrations was 3.6–5.5 mmol/L in HFrEF and 3.5–5.3 mmol/L in HFpEF. In HFrEF, 502 patients (1.6%) had a potassium concentration <3.5 mmol/L, 3301 participants (10.2%) had a potassium ≥3.5–<4.0 mmol/L, 11 511 (35.6%) potassium ≥4.0–<4.5 mmol/L, 11 963 (37.0%) potassium ≥4.5–<5.0 mmol/L, 4119 (12.7%) potassium ≥5.0–<5.5 mmol/L, and 950 patients (2.9%) had a potassium concentration ≥5.5 mmol/L. In HFpEF, the respective distribution of patients across these potassium categories was: 290 (2.1%) <3 .5 mmol/L, 1968 (14.3%) ≥3.5–<4.0 mmol/L, 5356 (39.0%) ≥ 4.0–<4.5 mmol/L, 4602 (33.5%) ≥ 4.5–<5.0 mmol/L, 1326 (9.7%) ≥ 5.0–<5.5 mmol/L, and 181 (1.3%) ≥ 5.5 mmol/L. Both mean and median potassium values were 4.5 mmol/L in HFrEF and 4.4 mmol/L in HFpEF, respectively (see Supplementary data online, *Figure S2*). The median follow-up was 24.2 and 36.8 months in HFrEF and HFpEF trials, respectively.

### Baseline characteristics according to potassium concentration

#### HFrEF

The baseline characteristics of each LVEF phenotype are presented in *Table 1*. From the lowest to the highest potassium category, the proportion of men increased (from 72.5% to 78.5%), as did the proportion of participants with a history of diabetes (from 39.4% to 43.9%), history of myocardial infarction (36.7% to 47.3%) and those in NYHA class III/IV (from 36.7% to 43.6%). When compared to patients with lower potassium levels, those with higher potassium levels had a slightly lower systolic blood pressure and much lower eGFR, and higher mean LVEF.

**Table 1:** Baseline characteristics according to the baseline potassium levels in patients with HFrEF and HFpEF

	HFrEF (LVEF ≤ 40%)						*P*-value for trend
	K < 3.5 *N* = 502	K ≥ 3.5-<4.0 *N* = 3301	K ≥ 4.0-<4.5 *N* = 11 511	K ≥ 4.5-<5.0 *N* = 11 963	K ≥ 5.0-<5.5 *N* = 4119	K ≥ 5.5 N = 950	
Age (years)	63.1 ± 12.5	63.9 ± 11.8	64.1 ± 11.7	65.1 ± 10.9	65.6 ± 10.7	65.4 ± 10.8	<.001
Male sex, *n* (%)	364 (72.5)	2531 (76.7)	8890 (77.2)	9389 (78.6)	3236 (78.6)	746 (78.5)	<.001
Region, *n* (%)							.015
North America	115 (22.9)	566 (17.2)	1811 (15.7)	1549 (13.0)	454 (11.0)	102 (10.7)	
Latin and Central America	80 (15.9)	494 (15.0)	1712 (14.9)	1840 (15.4)	705 (17.1)	186 (19.6)	
Western Europe	73 (14.5)	637 (19.3)	2403 (20.9)	2584 (21.6)	776 (18.8)	139 (14.6)	
Central and Eastern Europe (including Russia)	89 (17.7)	731 (22.1)	3206 (27.9)	3977 (33.2)	1561 (37.9)	395 (41.6)	
Asia-Pacific and other	145 (28.9)	873 (26.5)	2379 (20.7)	2013 (16.8)	623 (15.1)	128 (13.5)	
Race, *n* (%)							<.001
White	270 (53.8)	2037 (61.7)	7985 (69.4)	8952 (74.8)	3188 (77.4)	750 (79.0)	
Black	62 (12.4)	239 (7.2)	588 (5.1)	487 (4.1)	139 (3.4)	38 (4.0)	
Asian	124 (24.7)	779 (23.6)	2144 (18.6)	1741 (14.6)	506 (12.3)	112 (11.8)	
Other	46 (9.2)	246 (7.5)	794 (6.9)	783 (6.6)	286 (6.9)	50 (5.3)	
Heart failure characteristics							
NYHA functional class III or IV	184 (36.7)	1127 (34.2)	3780 (32.9)	4065 (34.0)	1551 (37.7)	413 (43.6)	<.001
KCCQ-CSS	69.1 (47.4–83.6)	73.4 (53.1–89.1)	75.0 (57.3–89.6)	75.9 (57.6–89.6)	74.0 (55.2–88.5)	69.4 (53.1–86.1)	.76
Physiological measurements							
Body mass index (kg/m^2^)	27.7 ± 6.3	27.9 ± 6.1	28.1 ± 5.8	28.1 ± 5.5	28.0 ± 5.6	27.5 ± 5.5	.078
Heart rate (bpm)	75.9 ± 13.0	73.2 ± 12.2	72.0 ± 12.0	71.7 ± 12.3	71.7 ± 12.1	73.1 ± 12.6	<.001
Systolic blood pressure (mmHg)	121.5 ± 17.8	122.0 ± 17.9	121.8 ± 17.1	120.6 ± 16.3	119.9 ± 15.6	119.5 ± 15.6	<.001
Diastolic blood pressure (mmHg)	73.8 ± 11.0	74.2 ± 11.1	73.7 ± 10.6	73.1 ± 10.4	72.8 ± 10.0	72.6 ± 10.3	<.001
Left ventricular ejection fraction (%)	27.3 ± 6.9	28.0 ± 6.5	28.3 ± 6.4	28.4 ± 6.3	28.7 ± 6.4	29.0 ± 6.3	<.001
Laboratory data							
Potassium (mmol/L)	3.2 ± 0.2	3.8 ± 0.1	4.2 ± 0.1	4.7 ± 0.1	5.2 ± 0.1	5.8 ± 0.3	
Estimated glomerular filtration rate (mL/min/1.73 m^2^)^a^	70.1 ± 29.1	70.2 ± 22.4	70.5 ± 22.8	66.3 ± 21.1	60.9 ± 20.5	56.6 ± 21.0	<.001
Estimated glomerular filtration rate <60 mL/min/1.73 m^2a^	192 (38.3)	1086 (32.9)	3719 (32.3)	4783 (40.0)	2122 (51.5)	564 (59.4)	<.001
Baseline NT-proBNP (ng/L)	2428 (1121–5299)	1730 (914–3654)	1510 (810–2982)	1486 (805–2929)	1676 (881–3313)	1885 (1043–3826)	.78
Haemoglobin (g/dL)	13.4 ± 1.9	13.6 ± 1.7	13.8 ± 1.6	13.8 ± 1.7	13.8 ± 1.7	13.6 ± 1.8	.025
Sodium (mmol/L)	140.1 ± 5.9	140.4 ± 3.3	140.4 ± 3.1	140.2 ± 3.3	140.1 ± 3.6	139.4 ± 4.1	<.001
Serum creatinine (umol/L)	90.7 ± 50.7	87.3 ± 46.2	89.4 ± 40.0	95.5 ± 40.8	108.8 ± 38.9	122.0 ± 48.3	<.001
Comorbidities							
Diabetes mellitus	198 (39.4)	1091 (33.1)	3790 (32.9)	4365 (36.5)	1639 (39.8)	417 (43.9)	<.001
Hypertension	360 (71.7)	2319 (70.3)	7739 (67.2)	8083 (67.6)	2912 (70.7)	688 (72.4)	.13
Myocardial infarction	184 (36.7)	1337 (40.5)	4843 (42.1)	5422 (45.3)	1911 (46.4)	449 (47.3)	<.001
Atrial fibrillation/flutter	189 (37.7)	1209 (36.6)	4176 (36.3)	4452 (37.2)	1535 (37.3)	359 (37.8)	.22
Stroke	49 (9.8)	280 (8.5)	1000 (8.7)	1046 (8.8)	376 (9.1)	80 (8.4)	.72
Drugs and devices							
Angiotensin-converting enzyme inhibitor/angiotensin receptor blocker/angiotensin receptor–neprilysin inhibitor	421 (83.9)	2995 (90.8)	10 679 (92.8)	11 254 (94.1)	3917 (95.1)	885 (93.2)	<.001
Beta-blocker	442 (88.1)	2948 (89.4)	10 461 (90.9)	10 949 (91.6)	3808 (92.5)	866 (91.2)	<.001
Sodium–glucose cotransporter 2 inhibitor	4 (0.8)	14 (0.4)	72 (0.6)	81 (0.7)	42 (1.0)	4 (0.4)	.043
Mineralocorticoid receptor antagonist	210 (41.8)	1409 (42.7)	5637 (49.0)	6741 (56.4)	2577 (62.6)	647 (68.1)	<.001
Calcium channel blockers	61 (12.2)	359 (10.9)	1080 (9.4)	959 (8.0)	333 (8.1)	77 (8.1)	<.001
Anticoagulant agent	211 (48.7)	1320 (46.1)	4771 (45.6)	5301 (48.0)	2013 (50.1)	503 (53.5)	<.001
Antiplatelet agent	269 (53.6)	1774 (53.8)	6339 (55.1)	6638 (55.5)	2352 (57.1)	535 (56.3)	.004
Diuretics	457 (91.0)	2929 (88.8)	9785 (85.1)	10 109 (84.5)	3466 (84.2)	834 (87.8)	<.001
Loop diuretics	370 (85.5)	2386 (83.3)	8268 (79.0)	8770 (79.5)	3167 (78.9)	779 (82.8)	.013
Dose of oral loop diuretics (furosemide equivalent dose) (mg/day)^b^	80 (40–160)	40 (40–80)	40 (40–80)	40 (40–80)	40 (40–80)	40 (40–80)	<.001
Thiazide diuretics	40 (9.2)	239 (8.3)	725 (6.9)	596 (5.4)	208 (5.2)	49 (5.2)	<.001
Implantable cardioverter defibrillator	100 (19.9)	615 (18.6)	2244 (19.5)	2521 (21.1)	863 (21.0)	179 (18.8)	.008
Pacemaker	40 (8.0)	340 (10.3)	1216 (10.6)	1229 (10.3)	424 (10.3)	84 (8.8)	.67

	HFpEF (LVEF ≥50%)						*P*-value for trend
	K < 3.5 *N* = 290	K ≥ 3.5–<4.0 *N* = 1968	K ≥ 4.0–<4.5 *N* = 5356	K ≥ 4.5–<5.0 *N* = 4602	K ≥ 5.0–<5.5 *N* = 1326	K ≥ 5.5 *N* = 181	
Age (years)	72.6 ± 10.0	72.3 ± 9.1	72.1 ± 8.8	72.6 ± 8.5	72.3 ± 8.1	72.3 ± 8.7	.60
Male sex, *n* (%)	120 (41.4)	847 (43.0)	2366 (44.2)	2032 (44.2)	642 (48.4)	85 (47.0)	.006
Region, *n* (%)							<.001
North America	108 (37.2)	683 (34.7)	1386 (25.9)	881 (19.1)	180 (13.6)	29 (16.0)	
Latin and Central America	31 (10.7)	208 (10.6)	562 (10.5)	589 (12.8)	153 (11.5)	18 (9.9)	
Western Europe	64 (22.1)	436 (22.2)	1273 (23.8)	1124 (24.4)	351 (26.5)	47 (26.0)	
Central and Eastern Europe (including Russia)	47 (16.2)	385 (19.6)	1542 (28.8)	1574 (34.2)	516 (38.9)	71 (39.2)	
Asia-Pacific and other	40 (13.8)	256 (13.0)	593 (11.1)	434 (9.4)	126 (9.5)	16 (8.8)	
Race, *n* (%)							<.001
White	217 (74.8)	1523 (77.4)	4483 (83.7)	3986 (86.6)	1180 (89.0)	157 (86.7)	
Black	27 (9.3)	151 (7.7)	232 (4.3)	142 (3.1)	19 (1.4)	4 (2.2)	
Asian	36 (12.4)	217 (11.0)	461 (8.6)	310 (6.7)	82 (6.2)	15 (8.3)	
Other	10 (3.5)	77 (3.9)	180 (3.4)	164 (3.6)	45 (3.4)	5 (2.8)	
Heart failure characteristics							
NYHA functional class III or IV	109 (37.6)	863 (44.0)	2249 (42.0)	1859 (40.4)	544 (41.0)	81 (44.8)	.17
KCCQ-CSS	63.8 (44.0–80.2)	64.7 (47.4–82.3)	68.8 (51.0–84.9)	69.8 (52.6–84.4)	71.2 (55.7–84.9)	72.4 (54.2–84.4)	<.001
Physiological measurements							
Body mass index (kg/m^2^)	31.2 ± 7.1	31.2 ± 6.9	30.7 ± 6.1	30.5 ± 5.9	30.2 ± 5.3	30.1 ± 5.7	<.001
Heart rate (bpm)	72.1 ± 12.9	71.4 ± 11.8	70.6 ± 11.4	70.3 ± 11.3	70.1 ± 11.7	71.7 ± 12.3	.001
Systolic blood pressure (mmHg)	131.2 ± 18.5	131.8 ± 16.4	132.0 ± 15.5	132.0 ± 15.7	132.2 ± 15.3	130.8 ± 16.4	.97
Diastolic blood pressure (mmHg)	74.1 ± 11.3	75.0 ± 10.8	75.2 ± 10.6	75.5 ± 10.4	75.4 ± 10.5	75.8 ± 11.0	.039
Left ventricular ejection fraction (%)	58.9 ± 6.6	59.5 ± 7.2	59.3 ± 7.2	59.4 ± 7.3	59.1 ± 7.0	58.5 ± 7.6	.31
Laboratory data							
Potassium (mmol/L)	3.3 ± 0.2	3.8 ± 0.1	4.2 ± 0.1	4.7 ± 0.1	5.1 ± 0.1	5.7 ± 0.3	
Estimated glomerular filtration rate (mL/min/1.73 m^2^)^a^	62.8 ± 20.7	66.1 ± 20.6	67.4 ± 21.4	64.0 ± 20.8	59.7 ± 20.5	54.7 ± 21.3	<.001
Estimated glomerular filtration rate <60 mL/min/1.73 m^2a^	137 (47.2)	829 (42.1)	2121 (39.6)	2158 (46.9)	745 (56.2)	121 (66.85)	<.001
Baseline NT-proBNP (ng/L)	901 (378–1849)	752 (272–1692)	565 (241–1342)	585 (249–1279)	543 (233–1255)	692 (338–1527)	<.001
Haemoglobin (g/dL)	13.0 ± 1.7	13.4 ± 1.7	13.4 ± 1.7	13.4 ± 1.7	13.4 ± 1.8	13.4 ± 1.8	.092
Sodium (mmol/L)	140.5 ± 3.9	140.5 ± 3.1	140.5 ± 2.9	140.3 ± 3.1	140.1 ± 3.3	139.3 ± 4.0	<.001
Serum creatinine (umol/L)	98.9 ± 30.2	94.6 ± 29.6	92.7 ± 28.2	96.8 ± 29.8	104.6 ± 34.6	114.6 ± 36.9	<.001
Comorbidities							
Diabetes mellitus	113 (39.0)	703 (35.7)	1985 (37.1)	1773 (38.5)	591 (44.6)	98 (54.1)	<.001
Hypertension	265 (91.4)	1822 (92.6)	4860 (90.7)	4182 (90.9)	1219 (91.9)	164 (90.6)	.43
Myocardial infarction	44 (15.2)	355 (18.0)	1155 (21.6)	978 (21.3)	322 (24.3)	40 (22.1)	<.001
Atrial fibrillation/flutter	163 (56.2)	979 (49.8)	2484 (46.4)	2044 (44.4)	574 (43.3)	80 (44.2)	<.001
Stroke^c^	41 (14.2)	269 (13.7)	556 (10.4)	466 (10.1)	137 (10.3)	26 (14.4)	.003
Drugs and devices							
Angiotensin-converting enzyme inhibitor/angiotensin receptor blocker/angiotensin receptor–neprilysin inhibitor	187 (64.5)	1228 (62.4)	3547 (66.2)	3205 (69.7)	950 (71.6)	130 (71.8)	<.001
Beta-blocker	209 (72.1)	1423 (72.3)	3901 (72.9)	3455 (75.1)	985 (74.3)	114 (63.0)	.17
Sodium–glucose cotransporter 2 inhibitor	19 (6.6)	84 (4.3)	201 (3.8)	153 (3.3)	39 (2.9)	9 (5.0)	.007
Mineralocorticoid receptor antagonist	17 (5.9)	127 (6.5)	486 (9.1)	597 (13.0)	274 (20.7)	41 (22.7)	<.001
Calcium channel blockers	115 (40.0)	895 (45.5)	2113 (39.5)	1686 (36.6)	477 (36.0)	50 (27.6)	<.001
Anticoagulant agent	137 (47.2)	770 (39.1)	1843 (34.4)	1457 (31.7)	409 (30.8)	57 (31.5)	<.001
Antiplatelet agent	79 (27.2)	672 (34.2)	1731 (32.3)	1434 (31.2)	408 (30.8)	51 (28.2)	.056
Diuretics	279 (96.2)	1867 (94.9)	4880 (91.1)	4169 (90.6)	1219 (91.9)	168 (92.8)	<.001
Loop diuretics	158 (76.3)	1167 (73.4)	3339 (71.2)	3003 (71.7)	947 (72.7)	136 (76.0)	.81
Dose of oral loop diuretics (furosemide equivalent dose) (mg/day)^d^	40 (40–80)	40 (40–80)	40 (20–60)	40 (20–40)	40 (20–40)	40 (40–40)	<.001
Thiazide diuretics	63 (30.4)	408 (25.6)	1035 (22.1)	902 (21.5)	262 (20.1)	30 (16.8)	<.001
Implantable cardioverter defibrillator	4 (1.4)	16 (0.8)	30 (0.6)	22 (0.5)	5 (0.4)	1 (0.6)	.026
Pacemaker	35 (12.1)	166 (8.4)	414 (7.7)	345 (7.5)	106 (8.0)	20 (11.1)	.26

Values are mean ± standard deviation, *n* (%), or median (interquartile range).

Abbreviations: HFpEF, heart failure with preserved ejection fraction; KCCQ-CSS, Kansas City Cardiomyopathy Questionnaire Clinical Summary Score; NYHA, New York Heart Association; LVEF, left ventricular ejection fraction; NT-proBNP, *n*-terminal pro-B-type natriuretic peptide.

^a^In CHARM, the estimated glomerular filtration rate was calculated with Chronic Kidney Disease-Epidemiology Collaboration creatinine equation 2021.

^b^Only GALACTIC-HF, DAPA-HF, and PARADIGM-HF were analysed.

^c^In I-PRESERVE, transient ischaemic attack was included in the stroke.

^d^Only PARAGON-HF and FINEARTS-HF were analysed.

Patients with higher potassium levels were more frequently treated with renin-angiotensin system inhibitors, an angiotensin receptor–neprilysin inhibitor (ARNI), and, especially, MRAs. Loop diuretic use was only slightly more common in patients with low versus higher potassium concentrations, although loop diuretic dose and use of thiazide diuretics were greater in patients with lower potassium levels.

#### HFpEF

Among individuals with HFpEF, those with higher potassium levels were more often male, had a higher prevalence of diabetes and myocardial infarction, and a greater proportion in NYHA class III/IV, but a lower prevalence of atrial fibrillation/flutter, compared to those with lower potassium concentrations. Patients with higher potassium levels had a lower average eGFR, sodium concentration, and NT-proBNP level than those with lower potassium concentrations. People with higher potassium levels were more likely to be treated with a renin-angiotensin system blocker and MRA, but less likely to be treated with a calcium channel blocker and thiazide diuretic (although the median dose of oral loop diuretic was similar across all potassium groups).

### Clinical outcomes according to potassium level in HFrEF and HFpEF

**Figure 1:**
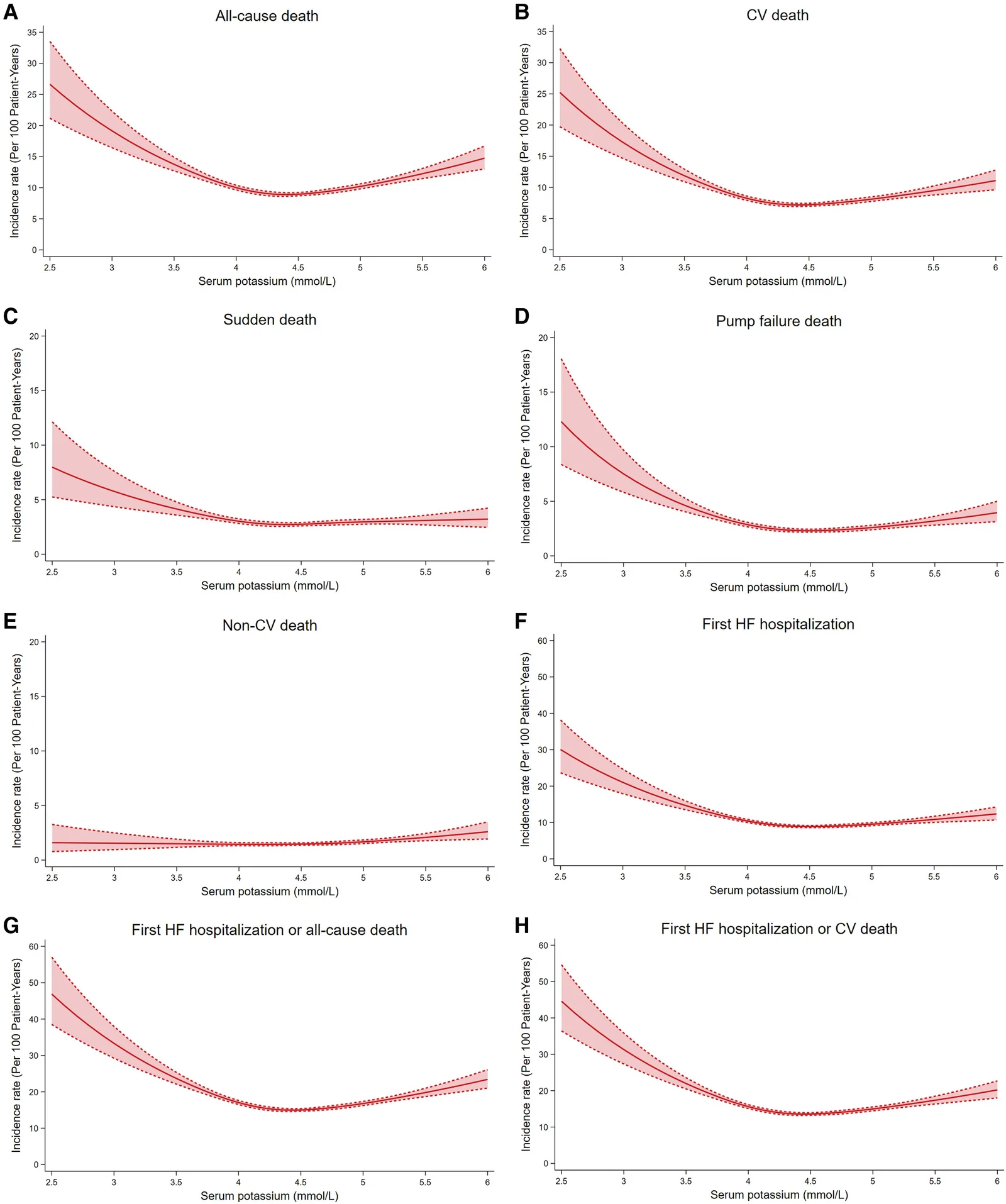
Incidence rate for each outcome across the range of potassium levels in HFrEF (analysed as a continuous variable). (*A*) All-cause death, (*B*) CV death, (*C*) Sudden death, (*D*) Pump failure death, (*E*) Non-CV death, (*F*) First HF hospitalization, (*G*) First HF hospitalization or all-cause death, and (*H*) First HF hospitalization or CV death. The X axis shows potassium, and the Y axis shows the unadjusted incidence rate for each outcome. The solid line shows a continuous incidence rate, and the interrupted lines on either side of this show the 95% CI. All the incidence rates are shown per 100 patient-years. CV indicates cardiovascular, HF, heart failure; and HFrEF, heart failure with reduced ejection fraction

**Figure 2:**
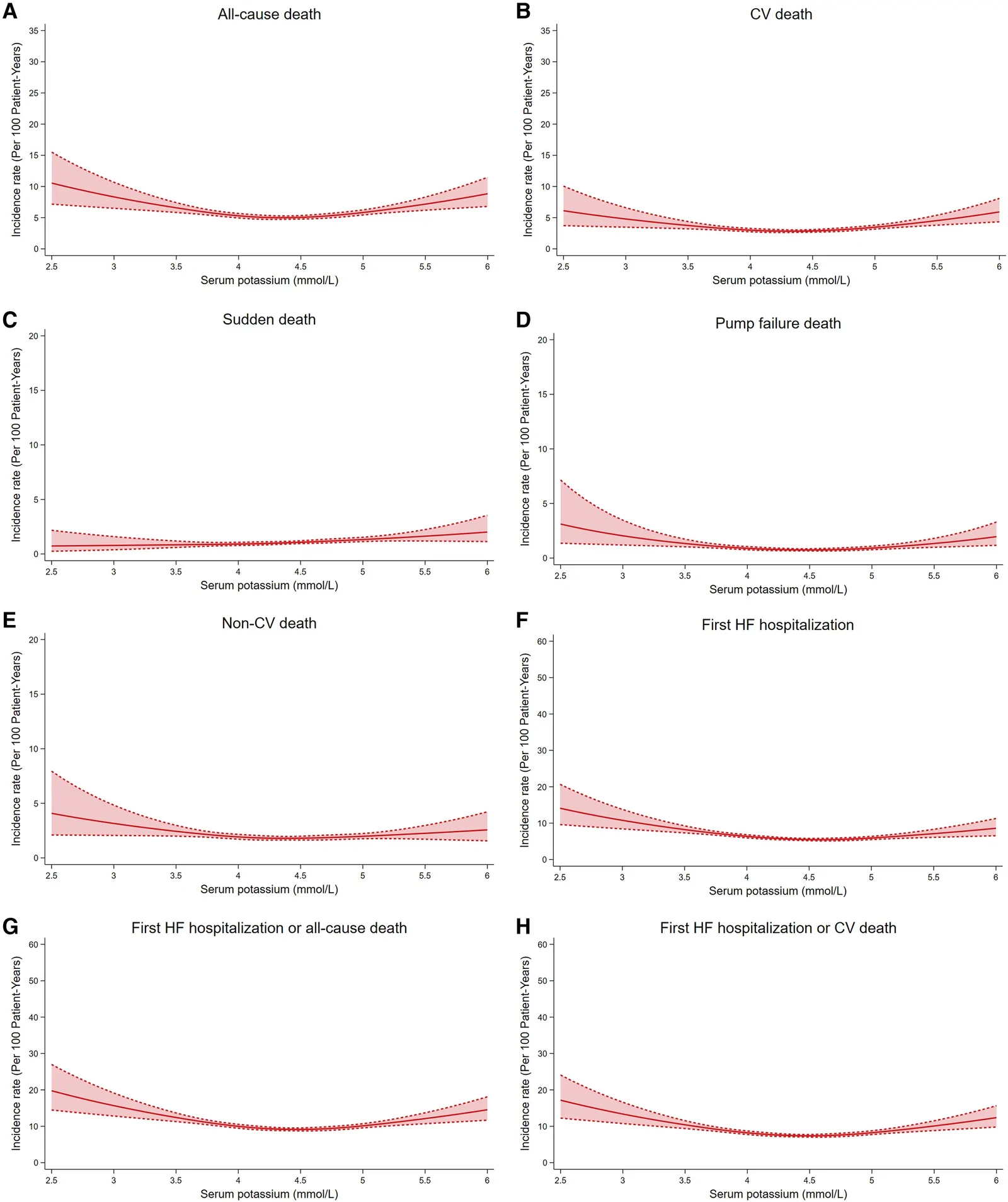
Incidence rate for each outcome across the range of potassium levels in HFpEF (analysed as a continuous variable). (*A*) All-cause death, (*B*) CV death, (*C*) Sudden death, (*D*) Pump failure death, (*E*) Non-CV death, (*F*) First HF hospitalization, (*G*) First HF hospitalization or all-cause death, and (*H*) First HF hospitalization or CV death. The X axis shows potassium, and the Y axis shows the unadjusted incidence rate for each outcome. The solid line shows a continuous incidence rate, and the interrupted lines on either side of this show the 95% CI. All the incidence rates are shown per 100 patient-years. CV indicates cardiovascular, HF, heart failure; and HFpEF, heart failure with preserved ejection fraction

The incidence rates for each outcome of interest, according to serum potassium concentration, illustrated as a continuous variable, are shown in *Figures 1* and *Figure 2* and Supplementary data online, *Figures S3–S5*. Cumulative incidence rate plots for each outcome of interest, according to potassium category, are depicted in Supplementary data online, *Figures S6* and *S7*, and the results of the time-to-first-event analyses are shown in *Table 2*. The HRs for each outcome, according to potassium level shown as a continuous variable, are illustrated in Supplementary data online, *Figures S8*–*S11*.

**Table 2:** Clinical outcomes according to the baseline potassium levels in patients with HFrEF and HFpEF

	Crude incidence rate, 100 patients-year (95% CI)	Unadjusted hazard ratios^a^ (95% CI)	Adjusted hazard ratios^b^ (95% CI)
	**HFrEF (LVEF ≤ 40%)**		
All-cause death			
K < 3.5 (*n* = 502)	17.7 (15.1–20.6)	1.86 (1.58–2.18)	1.49 (1.27–1.76)
K ≥ 3.5–<4.0 (*n* = 3301)	11.1 (10.3–11.9)	1.19 (1.10–1.29)	1.07 (0.98–1.16)
K ≥ 4.0–<4.5 (*n* = 11 511)	9.4 (9.0–9.7)	Reference	Reference
K ≥ 4.5–<5.0 (*n* = 11 963)	9.3 (9.0–9.7)	0.98 (0.93–1.04)	0.94 (0.89–1.00)
K ≥ 5.0–<5.5 (*n* = 4119)	10.5 (9.9–11.2)	1.07 (0.99–1.16)	0.96 (0.89–1.04)
K ≥ 5.5 (*n* = 950)	13.8 (12.2–15.6)	1.32 (1.16–1.51)	1.02 (0.89–1.16)
Cardiovascular death			
K < 3.5	15.4 (13.0–18.1)	1.98 (1.67–2.36)	1.58 (1.32–1.88)
K ≥ 3.5–<4.0	9.4 (8.7–10.1)	1.23 (1.13–1.34)	1.10 (1.00–1.20)
K ≥ 4.0–<4.5	7.6 (7.3–8.0)	Reference	Reference
K ≥ 4.5–<5.0	7.4 (7.1–7.8)	0.96 (0.90–1.02)	0.92 (0.87–0.98)
K ≥ 5.0–<5.5	8.5 (7.9–9.1)	1.07 (0.98–1.16)	0.96 (0.88–1.04)
K ≥ 5.5	10.4 (9.0–11.9)	1.25 (1.08–1.45)	0.95 (0.81–1.11)
Non-cardiovascular death			
K < 3.5	2.0 (1.2–3.1)	1.36 (0.84–2.18)	1.15 (0.70–1.91)
K ≥ 3.5–<4.0	1.4 (1.1–1.7)	0.97 (0.78–1.22)	0.92 (0.73–1.16)
K ≥ 4.0–<4.5	1.4 (1.3–1.6)	Reference	Reference
K ≥ 4.5–<5.0	1.6 (1.4–1.8)	1.09 (0.95–1.26)	1.03 (0.89–1.19)
K ≥ 5.0–<5.5	1.6 (1.3–1.9)	1.05 (0.86–1.28)	0.93 (0.76–1.14)
K ≥ 5.5	2.6 (2.0–3.5)	1.61 (1.19–2.18)	1.26 (0.92–1.72)
Sudden death			
K < 3.5	5.4 (4.1–7.1)	1.87 (1.40–2.50)	1.58 (1.18–2.12)
K ≥ 3.5–<4.0	3.4 (3.0–3.8)	1.18 (1.02–1.37)	1.07 (0.92–1.24)
K ≥ 4.0–<4.5	2.9 (2.7–3.1)	Reference	Reference
K ≥ 4.5–<5.0	2.8 (2.6–3.0)	0.99 (0.90–1.10)	0.98 (0.88–1.09)
K ≥ 5.0–<5.5	2.8 (2.4–3.1)	0.98 (0.85–1.14)	0.93 (0.80–1.07)
K ≥ 5.5	3.5 (2.7–4.4)	1.24 (0.96–1.61)	1.05 (0.81–1.36)
Pump failure death			
K < 3.5	6.4 (4.9–8.2)	2.38 (1.82–3.12)	1.71 (1.29–2.25)
K ≥ 3.5–<4.0	3.4 (3.0–3.9)	1.38 (1.19–1.60)	1.15 (0.98–1.33)
K ≥ 4.0–<4.5	2.5 (2.3–2.7)	Reference	Reference
K ≥ 4.5–<5.0	2.3 (2.2–2.5)	0.90 (0.81–1.01)	0.86 (0.76–0.96)
K ≥ 5.0–<5.5	2.9 (2.6–3.3)	1.04 (0.89–1.20)	0.92 (0.79–1.07)
K ≥ 5.5	3.5 (2.8–4.5)	1.12 (0.87–1.45)	0.84 (0.65–1.09)
First heart failure hospitalization			
K < 3.5	17.2 (14.5–20.4)	1.66 (1.39–1.98)	1.22 (1.02–1.46)
K ≥ 3.5–<4.0	12.3 (11.5–13.2)	1.28 (1.18–1.39)	1.13 (1.04–1.23)
K ≥ 4.0–<4.5	9.5 (9.1–9.9)	Reference	Reference
K ≥ 4.5–<5.0	8.9 (8.6–9.3)	0.91 (0.85–0.96)	0.88 (0.83–0.94)
K ≥ 5.0–<5.5	10.0 (9.4–10.8)	0.97 (0.90–1.05)	0.92 (0.84–1.00)
K ≥ 5.5	11.3 (9.8–13.0)	0.98 (0.85–1.14)	0.81 (0.70–0.95)
First heart failure hospitalization or all-cause death			
K < 3.5	29.3 (25.7–33.4)	1.77 (1.55–2.02)	1.37 (1.20–1.58)
K ≥ 3.5–<4.0	19.6 (18.5–20.7)	1.24 (1.16–1.32)	1.11 (1.03–1.18)
K ≥ 4.0–<4.5	15.8 (15.3–16.3)	Reference	Reference
K ≥ 4.5–<5.0	15.4 (14.9–15.9)	0.96 (0.91–1.00)	0.92 (0.88–0.97)
K ≥ 5.0–<5.5	17.4 (16.5–18.4)	1.04 (0.98–1.11)	0.96 (0.90–1.02)
K ≥ 5.5	21.6 (19.4–23.9)	1.19 (1.07–1.33)	0.95 (0.85–1.06)
First heart failure hospitalization or cardiovascular death			
K < 3.5	26.8 (23.4–30.7)	1.77 (1.54–2.04)	1.36 (1.18–1.58)
K ≥ 3.5–<4.0	18.1 (17.1–19.2)	1.26 (1.18–1.35)	1.12 (1.04–1.20)
K ≥ 4.0–<4.5	14.4 (13.9–14.9)	Reference	Reference
K ≥ 4.5–<5.0	13.9 (13.4–14.3)	0.94 (0.90–0.99)	0.91 (0.87–0.96)
K ≥ 5.0–<5.5	15.6 (14.8–16.5)	1.03 (0.96–1.10)	0.95 (0.89–1.02)
K ≥ 5.5	18.5 (16.5–20.7)	1.12 (1.00–1.26)	0.89 (0.79–1.01)
	**HFpEF (LVEF ≥ 50%)**		
All-cause death			
K < 3.5 (*n* = 290)	7.4 (5.7–9.5)	1.30 (1.00–1.70)	1.14 (0.87–1.48)
K ≥ 3.5–<4.0 (*n* = 1968)	5.8 (5.3–6.5)	1.11 (0.98–1.26)	1.07 (0.94–1.21)
K ≥ 4.0–<4.5 (*n* = 5356)	5.1 (4.8–5.5)	Reference	Reference
K ≥ 4.5–<5.0 (*n* = 4602)	5.3 (4.9–5.7)	1.06 (0.96–1.17)	1.03 (0.93–1.13)
K ≥ 5.0–<5.5 (*n* = 1326)	5.8 (5.1–6.5)	1.21 (1.05–1.40)	1.05 (0.91–1.22)
K ≥ 5.5 (*n* = 181)	9.0 (6.8–11.9)	1.92 (1.43–2.57)	1.29 (0.96–1.74)
Cardiovascular death			
K < 3.5	3.9 (2.8–5.6)	1.31 (0.91–1.88)	1.15 (0.79–1.65)
K ≥ 3.5–<4.0	3.5 (3.1–4.1)	1.21 (1.03–1.43)	1.19 (1.01–1.41)
K ≥ 4.0–<4.5	2.8 (2.6–3.1)	Reference	Reference
K ≥ 4.5–<5.0	3.0 (2.7–3.3)	1.07 (0.94–1.22)	1.05 (0.91–1.20)
K ≥ 5.0–<5.5	3.7 (3.2–4.3)	1.36 (1.14–1.64)	1.17 (0.97–1.42)
K ≥ 5.5	5.4 (3.8–7.8)	2.01 (1.38–2.93)	1.25 (0.84–1.85)
Non-cardiovascular death			
K < 3.5	3.1 (2.1–4.6)	1.48 (0.98–2.23)	1.30 (0.86–1.96)
K ≥ 3.5–<4.0	1.9 (1.6–2.3)	1.01 (0.82–1.26)	0.95 (0.76–1.18)
K ≥ 4.0–<4.5	1.9 (1.7–2.1)	Reference	Reference
K ≥ 4.5–<5.0	2.0 (1.7–2.2)	1.08 (0.92–1.27)	1.02 (0.86–1.20)
K ≥ 5.0–<5.5	1.8 (1.4–2.2)	1.05 (0.81–1.35)	0.91 (0.70–1.18)
K ≥ 5.5	2.6 (1.6–4.4)	1.55 (0.91–1.20)	1.18 (0.68–2.03)
Sudden death			
K < 3.5	0.7 (0.3–1.6)	0.75 (0.33–1.69)	0.71 (0.31–1.62)
K ≥ 3.5–<4.0	0.9 (0.7–1.2)	0.97 (0.71–1.31)	0.96 (0.70–1.30)
K ≥ 4.0–<4.5	1.0 (0.8–1.1)	Reference	Reference
K ≥ 4.5–<5.0	1.2 (1.0–1.3)	1.20 (0.97–1.50)	1.17 (0.94–1.46)
K ≥ 5.0–<5.5	1.2 (0.9–1.6)	1.29 (0.94–1.77)	1.06 (0.76–1.47)
K ≥ 5.5	2.6 (1.6–4.4)	2.76 (1.59–4.77)	1.87 (1.07–3.28)
Pump failure death			
K < 3.5	1.6 (0.9–2.8)	1.79 (1.01–3.17)	1.47 (0.83–2.63)
K ≥ 3.5–<4.0	1.1 (0.9–1.5)	1.39 (1.04–1.86)	1.28 (0.95–1.73)
K ≥ 4.0–<4.5	0.8 (0.7–1.0)	Reference	Reference
K ≥ 4.5–<5.0	0.8 (0.6–0.9)	0.97 (0.75–1.25)	0.98 (0.75–1.27)
K ≥ 5.0–<5.5	0.9 (0.7–1.2)	1.22 (0.85–1.76)	1.10 (0.76–1.60)
K ≥ 5.5	2.1 (1.1–3.7)	2.83 (1.53–5.24)	1.46 (0.77–2.79)
First heart failure hospitalization			
K < 3.5	8.3 (6.4–10.7)	1.25 (0.97–1.63)	1.09 (0.84–1.42)
K ≥ 3.5–<4.0	7.4 (6.7–8.2)	1.24 (1.10–1.39)	1.14 (1.01–1.28)
K ≥ 4.0–<4.5	5.8 (5.5–6.2)	Reference	Reference
K ≥ 4.5–<5.0	5.5 (5.2–6.0)	0.97 (0.88–1.07)	0.96 (0.87–1.06)
K ≥ 5.0–<5.5	6.2 (5.4–7.0)	1.14 (0.98–1.32)	1.00 (0.86–1.17)
K ≥ 5.5	7.9 (5.7–10.9)	1.44 (1.03–2.00)	0.99 (0.70–1.39)
First heart failure hospitalization or all-cause death			
K < 3.5	13.6 (11.1–16.5)	1.29 (1.05–1.58)	1.13 (0.92–1.39)
K ≥ 3.5–<4.0	11.1 (10.2–12.0)	1.15 (1.04–1.26)	1.08 (0.98–1.19)
K ≥ 4.0–<4.5	9.4 (9.0–9.9)	Reference	Reference
K ≥ 4.5–<5.0	9.4 (8.8–9.9)	1.01 (0.94–1.09)	0.99 (0.91–1.07)
K ≥ 5.0–<5.5	10.3 (9.3–11.4)	1.17 (1.04–1.31)	1.02 (0.91–1.15)
K ≥ 5.5	14.5 (11.4–18.4)	1.63 (1.28–2.09)	1.15 (0.89–1.48)
First heart failure hospitalization or cardiovascular death			
K < 3.5	10.9 (8.8–13.6)	1.29 (1.03–1.63)	1.13 (0.90–1.43)
K ≥ 3.5–<4.0	9.4 (8.6–10.3)	1.20 (1.08–1.34)	1.13 (1.02–1.26)
K ≥ 4.0–<4.5	7.6 (7.2–8.1)	Reference	Reference
K ≥ 4.5–<5.0	7.5 (7.1–8.0)	1.00 (0.92–1.09)	0.99 (0.91–1.08)
K ≥ 5.0–<5.5	8.7 (7.8–9.7)	1.22 (1.08–1.38)	1.07 (0.94–1.21)
K ≥ 5.5	11.3 (8.6–14.8)	1.56 (1.18–2.05)	1.07 (0.80–1.43)

CI, confidence interval; HFpEF, heart failure with preserved ejection fraction; HFrEF, heart failure with reduced ejection fraction; K, potassium; and LVEF, left ventricular ejection fraction.

^a^Models were stratified according to the trial and with the treatment assignment as covariate.

^b^Models were stratified according to the trial and with the treatment assignment as covariate, and adjusted for age, sex, region, race, heart rate, New York Heart Association functional classes III or IV, systolic blood pressure, body mass index, left ventricular ejection fraction, serum creatinine, haemoglobin, NT-proBNP (log-transformed), history of atrial fibrillation, history of myocardial infarction, history of diabetes, diuretics use, mineralocorticoid receptor antagonist use, angiotensin-converting enzyme inhibitor/angiotensin receptor blocker/angiotensin receptor neprilysin inhibitor use, and treatment arm. In the cases with missing data, the missing indicator method was used for NT-proBNP values.

### Clinical outcomes according to potassium concentration in HFrEF

#### Mortality

In HFrEF, the highest cumulative incidence rate of all-cause death was observed among those in the lowest potassium category (i.e. potassium <3.5 mmol/L) and the lowest incidence rate was observed among those in the potassium category ≥4.5–<5.0 mmol/L (*Table 2*). Compared to those in the potassium category ≥4.0–<4.5 (reference group), the aHR for all-cause death were 1.49 (95% CI 1.27–1.76), 1.07 (95% CI 0.98–1.16), 0.94 (95% CI 0.89–1.00), 0.96 (95% CI 0.89–1.04), and 1.02 (95% CI 0.89–1.16) in potassium categories <3.5 mmol/L, ≥3.5–<4.0 mmol/L, ≥4.5–<5.0 mmol/L, ≥5.0–<5.5 mmol/L, and ≥5.5 mmol/L, respectively (*Table 2* and Supplementary data online, *Figure S6*). No meaningful differences were observed in the estimates between the two-stage meta-analysis and the one-stage meta-analysis stratified by trial; therefore, the results of the one-stage meta-analysis stratified by trial are presented in the following section (see Supplementary data online, *Table S4*). The significantly higher risk in patients with serum potassium level <3.5 mmol/L was observed even after further adjustment for the dose of oral loop diuretic (see Supplementary data online, *Table S5*). When potassium level was analysed as a continuous variable, the unadjusted spline curve for the incidence rate of all-cause death showed a reverse J-curve relationship (*Figure 1*). The corresponding spline curves for HRs are shown in Supplementary data online, *Figures S8* and *S9*.

The association between potassium concentration and cardiovascular death was similar to that observed for all-cause death (*Figure 1*, Supplementary data online, *Figures S8* and *S9*). Incidence rates and aHR for both sudden death and pump failure death were also highest in the lowest potassium category (potassium <3.5 mmol/L) (*Table 2*). Examination of these outcomes according to continuous potassium concentration demonstrated a reverse J-curve association, with significantly elevated aHR for potassium <4.5 mmol/L, while the association with hyperkalaemia was attenuated and not statistically significant (see Supplementary data online, *Figure S8*).

By contrast, the incidence of death from non-cardiovascular causes was low and varied little across potassium categories (*Table 2* and *Figure 1*).

#### First heart failure hospitalization and composite outcomes

The association between serum potassium concentration and the incidence of HF hospitalization was very similar to the association observed for all-cause death and cardiovascular death, and this was also seen for composites of HF hospitalization and all-cause and cardiovascular mortality (*Table 2* and *Figure 1*).

#### Serum potassium level and clinical outcomes stratified by kidney function

Patients with an eGFR <60 mL/min/1.73 m^2^ had higher incidence rates for all outcomes compared to those with an eGFR ≥60 mL/min/1.73 m^2^ (see Supplementary data online, *Figure S3*). Across all-cause death, cardiovascular death, non-cardiovascular death, the composites of HF hospitalization and all-cause and cardiovascular mortality, there were significant interactions across strata of eGFR (see Supplementary data online, *Table S6*). In patients with eGFR <60 mL/min/1.73 m^2^, the serum potassium level (4.8 mmol/L) corresponding to the nadir of the incidence rate for these outcomes was higher than among those with an eGFR ≥60 mL/min/1.73 m^2^ (4.3–4.4 mmol/L). However, in both strata, these potassium levels fell within the overall optimal range (4.2–5.0 mmol/L).

#### Serum potassium level and clinical outcomes stratified by treatment arm or MRA use

In categorical analyses of serum potassium, no significant interactions were observed with either treatment arm or MRA use (see Supplementary data online, *Tables S7* and *S8*). When serum potassium was analysed as a continuous variable, no interaction was observed with treatment arm, but significant interactions by MRA use were observed for all-cause death and for cardiovascular death, with a higher nadir of potassium concentration for the incidence of these events in the MRA group compared with the no MRA group (see Supplementary data online, *Figure S5*). However, the difference in the serum potassium level corresponding to the nadir of the incidence rate between patients receiving MRAs and those not receiving MRAs was small (4.5 vs 4.4 mmol/L) (see Supplementary data online, *Table S6*).

#### Potassium concentration associated with lowest risk

For all-cause death, binary recursive partitioning identified 3.7–5.0 mmol/L as the lowest-risk range, whereas potassium associated with the nadir of the aHR was 4.7 mmol/L and for nadir of the incidence rate it was 4.4 mmol/L (see Supplementary data online, *Table S9*). When considering all clinical outcomes, the overlapping range associated with the lowest risk was 4.2–5.0 mmol/L.

### Clinical outcomes according to potassium concentration in HFpEF

#### Mortality

Compared to HFrEF, crude incidence rates were substantially lower across all potassium categories. In contrast to HFrEF, the cumulative incidence rate for all-cause death in HFpEF was highest in those in the highest potassium category (i.e. potassium ≥5.5 mmol/L) (see Supplementary data online, *Figure S7*). The aHR for all-cause death compared with potassium ≥4.0–<4.5 (reference category) were 1.14 (95% CI 0.87–1.48), 1.07 (95% CI 0.94–1.21), 1.03 (95% CI 0.93–1.13), 1.05 (95% CI 0.91–1.22), and 1.29 (95% CI 0.96–1.74) in potassium categories <3.5 mmol/L, ≥ 3.5–<4.0 mmol/L, ≥ 4.5–<5.0 mmol/L, ≥5.0–<5.5 mmol/L, and ≥5.5 mmol/L, respectively (*Table 2*). Examination of potassium level as a continuous variable showed a flattened U-shape relationship for all-cause mortality, with relatively little variation across the baseline potassium level (*Figure 2*, Supplementary data online, *Figures S10* and *S11*). The picture was similar for cardiovascular death and pump failure death, with an even flatter relationship for sudden death, the incidence of which was very low (*Table 2* and *Figure 2*).

#### First heart failure hospitalization and composite outcomes

There was a flattened U-shape association between first HF hospitalization, and the composite outcomes analysed, in patients with HFpEF (*Figure 2* and Supplementary data online, *Figure S10*).

#### Serum potassium level and clinical outcomes stratified by kidney function

Similar to HFrEF, HFpEF patients with an eGFR <60 mL/min/1.73 m^2^ had higher incidence rates for all outcomes compared to those with an eGFR ≥60 mL/min/1.73 m^2^, but the associations between potassium concentration and outcomes were similar in each eGFR stratum, with no significant interaction (see Supplementary data online, *Figure S4* and Supplementary data online, *Table S5*).

#### Serum potassium level and clinical outcomes stratified by treatment arm or MRA use

In analyses of serum potassium levels and clinical outcomes stratified by treatment arm or MRA use, no significant interactions were observed with either treatment arm or MRA use in the categorical analyses (see Supplementary data online, *Tables S10* and *S11*). Similarly, when serum potassium was analysed as a continuous variable to estimate incidence rates, no significant interactions with treatment arm or MRA use were observed (see Supplementary data online, *Table S6*).

#### Potassium concentration associated with lowest risk

In HFpEF, the nadir of the aHR and incidence rate converged around a potassium concentration of 4.3–4.6 mmol/L, while binary recursive partitioning produced broader boundaries, e.g. 3.8–5.3 mmol/L for all-cause death (see Supplementary data online, *Table S9*). Considering all clinical outcomes, the overlapping range associated with the lowest risk was 4.2–5.1 mmol/L.

### Serious adverse events related to hyperkalaemia and hypokalaemia across trials

Serious adverse events (SAEs) related to hyperkalaemia were reported in 203 of 26 515 patients (0.8%) in the control group and 238 of 28 878 patients (0.8%) in the intervention group; among these, fatal SAEs occurred in 4 (0.02%) and 10 (0.03%) patients, respectively.

Similarly, SAEs related to hypokalaemia were reported in 108 of 26 515 patients (0.4%) in the control group and 75 of 28 878 patients (0.3%) in the intervention group; among these, fatal SAEs occurred in 1 patient in each group (0.004% in the control and 0.003% in the intervention group) (see Supplementary data online, *Table S12*).

## Discussion

In this large, pooled analysis of 12 HF trials, we found that the association between serum potassium levels and clinical outcomes differed notably between HFrEF and HFpEF. In HFrEF, there was a reverse J-shaped association between potassium concentration and adverse clinical outcomes, with a strong association between hypokalaemia and higher rates of death and HF hospitalization, whereas potassium concentrations up to 5.5 mmol/L were not associated with worse outcomes after multivariable adjustment. The lowest event rates in people with HFrEF were observed over the potassium range 4.2–5.0 mmol/L. In HFpEF, event rates were lower than in HFrEF for any given potassium concentration, and the associations between potassium concentration and outcomes were much flatter, with a shallow U-shaped relationship observed. The lowest event rates in HFpEF were observed over the potassium range 4.2–5.1 mmol/L (*Structured Graphical Abstract*).

These results have several clinical implications. Concerning HFrEF, although our findings are observational in nature and do not necessarily represent cause and effect, they are robust, with the differential risks related to lower and higher potassium levels persisting through extensive adjustment for other prognostic variables and use of treatments, including diuretics (and dose of diuretics). This reduces the likelihood that the observed risk related to low potassium level was solely due to confounding, and our findings reinforce prior observations in other smaller HFrEF datasets with less complete multivariable adjustment. The lack of association between potassium concentration and non-cardiovascular death also argues against a non-specific finding. More importantly, a large trial, POTCAST (Targeted Potassium Levels to Decrease Arrhythmia Burden in High-Risk Patients with Cardiovascular Diseases) recently showed that elevating potassium (to a ‘high-normal’ plasma concentration of 4.5 to 5.0 mmol/L) reduced the risk of a composite of sustained ventricular tachycardia or appropriate implantable cardioverter–defibrillator therapy, hospitalization for arrhythmia or HF, or death from any cause, supporting the findings of an earlier trial showing that a potassium-enriched salt substitute reduced cardiovascular events compared to salt alone.^7^ However, it should be noted that POTCAST reported plasma potassium concentrations rather than serum potassium concentrations. As serum potassium concentrations are generally reported to be approximately 0.1–0.4 mmol/L higher than plasma potassium concentrations,^40^ we assumed an approximate difference of 0.2 mmol/L for conversion between the two measurements. Accordingly, the targeted plasma potassium range in POTCAST (4.5–5.0 mmol/L) would correspond to an approximate serum potassium range of 4.7–5.2 mmol/L. The targeted potassium range in POTCAST closely aligns with the range associated with a nadir in event rates in the present analyses. However, although patients with plasma potassium levels ≤4.3 mmol/L (approximately corresponding to serum potassium ≤4.5 mmol/L) were included in POTCAST, the lower end of the serum potassium range associated with the lowest event rates in our study (approximately 4.2–4.5 mmol/L, corresponding to plasma potassium levels of 4.0–4.3 mmol/L) was not the primary range targeted for potassium elevation or specifically evaluated as an optimal range in POTCAST. Therefore, differences in measurement modality (plasma vs serum) and in the potassium ranges actively targeted between studies should be considered when interpreting the apparent concordance between the two studies. While it could also be argued that the similar association between potassium concentration and HF hospitalization is mechanistically harder to explain than the association with death, potassium influences cardiac contractility, and arrhythmias may also precipitate worsening HF. Indeed, in another recent report, a retrospective analysis from the LOOP trial (Atrial Fibrillation Detected by Continuous Electrocardiogram Monitoring Using Implantable Loop Recorder to Prevent Stroke in High Risk Individuals) found that implantable loop recorder-documented atrial fibrillation was associated with lower potassium concentrations.^42^ Each mmol/L decrease in plasma potassium was associated with a five-fold increase in odds of atrial fibrillation, and the risk of atrial fibrillation was mainly associated with potassium concentrations below 4 mmol/L. Beyond the arrhythmic risk, hypokalaemia may also adversely influence the course of HF through several systemic mechanisms. Low serum potassium can stimulate renin–angiotensin–aldosterone system activity, contribute to sodium retention and increased arterial tone. Furthermore, hypokalaemia may serve as a marker of excess aldosterone and/or mineralocorticoid receptor activation, which could partly explain the observed association with adverse outcomes.^43^ Collectively, these observational and interventional data question the focus on hyperkalaemia in HFrEF, current concepts about what is a ‘normal’ potassium concentration in these patients, and whether patients are potentially being denied life-saving treatments because of unnecessary concerns about potassium concentration. The totality of the data argues that, from a safety perspective, the optimum serum potassium concentration is in the range of 4.5 to 5.0 mmol/L in these patients, and that consideration should be given to defining ‘hypokalaemia’ as a potassium concentration <4.0 mmol/L. In support of this, a recent study in patients with chronic kidney disease also reported that serum potassium concentration <4.0 mmol/L was strongly associated with adverse renal and cardiovascular outcomes.^44^ Therefore, practitioners should be at least as concerned about low potassium concentrations as high potassium in HFrEF.

The picture related to HFpEF appeared different, with a much weaker and U-shaped (rather than reverse J-shaped) association between potassium level and outcomes. The flatter risk gradients in HFpEF may reflect distinct underlying mechanisms compared to HFrEF, where neurohormonal activation is more pronounced and patients are more susceptible to ventricular arrhythmias. In contrast, patients with HFpEF may have a greater comorbidity burden and few cardiovascular deaths as a contribution to overall mortality. Potentially, serum potassium level may function more as a surrogate marker of comorbidity burden and concomitant treatments rather than a direct mediator of risk. Nonetheless, even in HFpEF, modest potassium deviations from the nadir range were associated with higher risk, indicating that dyskalaemia should not be entirely overlooked in this population, and the optimum serum potassium concentration range appears to be the same as for HFrEF, i.e. approximately 4.2–5.0 mmol/L. Here, it is worth noting that many patients in POTCAST had preserved rather than reduced LVEF.^7^

What are the practical implications of our findings for the management of patients with HF? First, they underscore the importance of avoiding hypokalaemia, particularly in HFrEF. A serum potassium concentration of 4.2–5.0 mmol/L appears optimal, from a safety perspective, for patients with both HFrEF and HFpEF. Second, mild hyperkalaemia should not reflexively trigger discontinuation of guideline-directed medical therapy, especially in HFrEF, where such treatments are potentially life-saving.^5,45–47^ We found that approximately 13% of patients with HFrEF had a serum potassium concentration between 5.0 and 5.5 mmol/L, which was not independently associated with higher mortality. A strategy of excluding other causes of hyperkalaemia and close monitoring of potassium level may be preferable to discontinuing essential therapies. The role of potassium-binding treatments is still unclear, with an increased risk of hypokalaemia and, in the case of sodium zirconium cyclosilicate, an elevated incidence of worsening HF, possibly due to the sodium content of the drug causing volume overload.^5,48^ In HFpEF, strict potassium targets may be less critical than in HFrEF, given the overall lower event rates and attenuated potassium–outcome associations in this HF phenotype, although general prudence remains appropriate.

### Limitations

First, this study represents a *post hoc* analysis, which may be subject to residual confounding. The analysed data were derived from patients enrolled in randomized controlled trials with specific inclusion and exclusion criteria, and the findings may not be generalizable to the broader HF patients in the general population. Notably, several trials excluded patients with hyperkalaemia at baseline, potentially underestimating the prognostic impact of severe potassium disturbances. Most trials also excluded patients with severe kidney dysfunction, although, in practice, these patients generally do not receive therapies likely to increase potassium level. However, our findings are complemented by a ‘real-world’ analysis of over 6000 patients with HF, which showed findings with higher risk associated with hypokalaemia and no increase in risk associated with those with a mild hyperkalaemia (5.0–5.5 mmol/L).^49^ In addition, serum potassium levels may be affected by pre-analytical factors such as sample haemolysis, phlebotomy technique, or delays in processing, which were not uniformly controlled across studies. Furthermore, this analysis focused exclusively on patients with HFrEF (LVEF ≤40%) and HFpEF (LVEF ≥50%) and did not include those with HF with mildly reduced ejection fraction (HFmrEF), limiting the applicability of our findings to HFmrEF.

## Conclusions

In conclusion, this study demonstrates differential associations between serum potassium level and clinical outcomes in HFrEF and HFpEF. In HFrEF, a reverse J-shaped risk curve was observed, emphasizing the importance of avoiding hypokalaemia. In contrast, a flatter U-shaped relationship between potassium level and outcomes was observed in HFpEF. In both HF phenotypes, the optimum potassium concentration appeared to be 4.2–5.0 mmol/L, but even concentrations of ≥5.0–<5.5 mmol/L were associated with minimal risk. Based upon risk, there is an argument to redefine hypokalaemia as a potassium concentration <4.0 mmol/L.

## Supplementary Material

ehag341_Supplementary_Data

## Declarations

### Data Availability

The original data are available through the specific application process offered by each sponsor of the trials included in the present analyses.

### Pre-registered Clinical Trial Number

URL: https://www.clinicaltrials.gov; Unique identifiers: NCT00853658 (ATMOSPHERE), NCT00634309 (CHARM Added), NCT00634400 (CHARM-Alternative), NCT00634712 (CHARM Preserved), NCT00232180 (EMPHASIS-HF), NCT00095238 (I-PRESERVE), NCT00094302 (TOPCAT), NCT01035255 (PARADIGM-HF), NCT01920711 (PARAGON-HF), NCT03036124 (DAPA-HF), NCT02929329 (GALACTIC-HF), and NCT04435626 (FINEARTS-HF).
