# Supplementary material for: Optimal serum potassium concentrations in heart failure: an individual patient data meta-analysis
Source: Eur Heart J. 2026 May 28;47(32):4480–99. doi: 10.1093/eurheartj/ehag341 (PMC13500161; doi:10.1093/eurheartj/ehag341)
Supplement: Supplementary file 1 [file ehag341_supplementary_data.docx]

**SUPPLEMENTAL MATERIAL**

**Supplemental Tables:1-12**

**Supplemental Figures:1-11**

**Supplemental Table 1**. **Summary of design of the included trials**

|  | **HFrEF** | | | | | |
| --- | --- | --- | --- | --- | --- | --- |
|  | CHARM-Added /Alternative | EMPHASIS-HF | ATMOSPHERE | PARADIGM-HF | DAPA-HF | GALACTIC-HF |
| N | 4,576 | 2,737 | 7,016 | 8,399 | 4,744 | 8,232 |
| Intervention | Candesartan | Eplerenone | Aliskiren/enalapril Aliskiren | Sacubitril/valsartan | Dapagliflozin | Omecamtiv Mecarbil |
| Compared with | Placebo | Placebo | Enalapril | Enalapril | Placebo | Placebo |
| Period | 1999-2003 | 2006-2010 | 2009-2015 | 2009-2014 | 2017-2018 | 2017-2020 |
| Duration | Median 41 (Added)/33.7(Alternative) months | Median 21 months | Median 36.6 months | Median 27 months | Median 18.2 months | Median 12.8 months |
| Core inclusion criteria (age, NYHA, and LVEF) | Age≥18 years NYHA class: II-IV; LVEF≤40% | Age≥55 years NYHA class: II; LVEF≤35% | Age≥18 years NYHA class: II-IV; LVEF≤35% | Age≥18 years NYHA class: II-IV; LVEF≤40%/35% (since December 2012) | Age≥18 years NYHA class: II-IV; LVEF≤40%; established DM or HbA1c ≥6.5% | Age 18-85 years NYHA class: II-IV; LVEF≤35% |
| Prior HF hospitalization (HHF), Natriuretic peptides (pg/mL) in inclusion criteria | Hospitalization for a cardiac reason within previous 6 months if NYHA class II | Hospitalization for a cardiac reason within previous 6 months; if not, BNP ≥250 or NT-proBNP ≥500 in men/750 in women | If HHF within prior 12 mo, BNP ≥100 or NT-proBNP ≥400;  if not, BNP ≥150 or NT-proBNP ≥600 | If HHF within prior 12 mo, BNP ≥100 or NT-proBNP ≥400;  if not, BNP ≥150 or NT-proBNP ≥600 | If HHF within prior 12 mo, NT-proBNP ≥400; if not, BNP ≥150 or NT-proBNP ≥600; if AF/flutter on baseline ECG, NT-proBNP ≥900 | Currently HHF or urgent visit for HF/HHF within 1 year |
| Potassium-related exclusion criteria | Potassium ≥5.5 mmol/L | Potassium ≥5.0 mmol/L | potassium ≥5.0 mmol/L at screening, or ≥5.2 mmol/L at randomization | Potassium >5.2 mmol/L at screening, or >5.4 mmol/L at randomization | None | None |
| Other key exclusion criteria | Symptomatic hypotension; serum Cr ≥265 umol/L | NYHA class III or IV; eGFR <30 ml/min/1.73m2 | Symptomatic hypotension; SBP <95 mmHg at visit 1 or <90 mmHg at randomization; eGFR <40 ml/min/1.73m^2^ at screening, or <35 ml/min/1.73m^2^ at randomization, or decrease >25% from screening to randomization | Symptomatic hypotension; SBP <100 mmHg at screening or <95 mmHg at randomization; eGFR <30 ml/min/1.73m^2^ at screening, or <35 ml/min/1.73m^2^ at randomization | Symptoms of hypotension or SBP < 95 mmHg; eGFR < 30 ml/min/1.73m^2^ (or rapidly declining renal function) | SBP >140 or < 85 mmHg; DBP > 90 mmHg; eGFR < 20 ml/min/1.73m^2^ |
| Primary outcomes | 1st of either CVD or HHF | 1st of either CVD or HHF | 1st occurrence of either CVD or HHF | 1st occurrence of either CVD or HHF | Worsening HF (hospitalization or urgent visit resulting in IV therapy) or CVD | 1st occurrence of either HF event (urgent clinic visit, emergency department visit or hospitalization for worsening HF or treatment intensification beyond a change in oral diuretic therapy) or CVD |

|  | **HFpEF** | | | | |
| --- | --- | --- | --- | --- | --- |
|  | CHARM-Preserved | I-Preserve | TOPCAT | PARAGON-HF | FINEARTS-HF |
| N | 3,032 | 4,128 | 1,767 | 4,796 | 6,001 |
| Intervention | Candesartan | Irbesartan | Spironolactone | Sacubitril/valsartan | Finerenone |
| Compared with | Placebo | Placebo | Placebo | Valsartan | Placebo |
| Period | 1999-2003 | 2002-2008 | 2006-2013 | 2014-2016 | 2020-2024 |
| Duration | Median 36.6 months | Median 49.5 months | Mean 3.3 years | Median 35 months | Median 32 months |
| Core inclusion criteria (age, NYHA, and LVEF) | Age≥18 years NYHA class: II-IV; LVEF>40% | Age≥60 years NYHA class: II-IV; LVEF≥45% | Age≥50 years LVEF≥45% | Age≥50 years NYHA class: II-IV; LVEF≥45% | Age≥40 years NYHA class: II-IV; LVEF≥40% |
| Prior HF hospitalization (HHF), Natriuretic peptides (pg/mL) in inclusion criteria | Hospitalization for a cardiac reason within previous 6 months if NYHA class II | HHF within previous 6 months;  if not, ongoing class III/IV symptoms with corresponding evidence | HHF within prior 12 months; if not, a BNP ≥100 or NT-proBNP ≥360 within 60 days before randomization | Required diuretic therapy ≥ 30 days before screening; If HHF within past 9 months, NT-proBNP >200;  if without recent HHF, NT-proBNP >300; tripled NT-proBNP if AF at screening; evidence of SHD | On diuretic treatment ≥ 30 days prior to randomization; HHF within 90 days or urgent visit for HF requiring IV diuretic therapy; NT-proBNP ≥300 pg/mL (BNP ≥100 pg/mL) in SR or or NT-proBNP ≥900 pg/mL (BNP ≥300 pg/mL) in AF |
| Potassium-related exclusion criteria | Potassium ≥5.0 mmol/L | None | Potassium ≥5.0 mmol/L | Potassium >5.2 mmol/L at screening, or >5.4 mmol/L at randomization | Potassium ≥5.0 mmol/L |
| Other key exclusion criteria | Symptomatic hypotension; serum Cr ≥265 umol/L | SBP <100 mmHg, or SBP >160 mmHg or DBP >95 mmHg despite antihypertensive therapy;  serum Cr>221 umol/L | SBP >160 mmHg; serum Cr ≥221 umol/L, or eGFR <30 ml/min/1.73m^2^ | Prior LVEF <40% using ECHO; an alternative diagnosis that could account for symptoms, and SBP <110 mmHg or ≥180 mmHg; SBP >150 mmHg were excluded unless receiving ≥ 3 antihypertensive medications at screening | Symptomatic hypotension with SBP < 90 mmHg; SBP >160 mmHg  if not on treatment with ≥3 blood pressure lowering medications or ≥180 mmHg irrespective of treatments on 2 consecutive measurements at least 2-minute apart; eGFR <25 mL/min/1.73 m² |
| Primary outcomes | 1st of either CVD or HHF | 1st of either all-cause death or CVH | 1st occurrence of either CVD, HHF, or RSD | CVD and total HHF (1st and recurrent) | Total worsening heart failure events and death from cardiovascular causes |

ATOMOSPHERE = Aliskiren Trial to Minimize Outcomes in Patients with Heart Failure; CHARM = Candesartan in Heart failure: Assessment of Reduction in Mortality and morbidity; DAPA-HF = Dapagliflozin And Prevention of Adverse outcomes in Heart Failure; EMPHASIS-HF = Eplerenone in Mild Patients Hospitalization and Survival Study in Heart Failure, FINEARTS-HF = Finerenone Trial to Investigate Efficacy and Safety Superior to Placebo in Patients with Heart Failure, GALACTIC-HF = Global Approach to Lowering Adverse Cardiac Outcomes Through Improving Contractility in Heart Failure, I-Preserve = Irbesartan in heart failure with Preserved ejection fraction; PARADIGM-HF = Prospective Comparison of ARNI With ACEI to Determine Impact on Global Mortality and Morbidity in Heart Failure; PRAGON-HF = Prospective Comparison of ARNI with ARB Global Outcomes in HF with Preserved Ejection Fraction; TOPCAT = Treatment of Preserved Cardiac Function Heart Failure with an Aldosterone Antagonist; HFrEF, heart failure with reduced ejection fraction; HFpEF, heart failure with preserved ejection fraction.

AF, atrial fibrillation; BNP, B-type natriuretic peptide; BP, blood pressure; Cr, creatinine; CVD, cardiovascular death; CVH, cardiovascular hospitalization; DBP, diastolic blood pressure; DM, diabetes mellitus; ECG, electrocardiogram; eGFR, estimated glomerular filtration rate; HbA1c, hemoglobin A1c; HHF, hospitalization for heart failure; IV, intravenous; LVEF, left ventricular ejection fraction; NT-proBNP, N-terminal pro B-type natriuretic peptide; SBP, systolic blood pressure; SHD, structural heart disease; NYHA, New York Heart Association; RSD, resuscitated sudden death.

**Supplemental Table 2**. **Missing number of analyzed variables in each trial**

|  | **HFrEF** | | | | | | **HFpEF** | | | | |
| --- | --- | --- | --- | --- | --- | --- | --- | --- | --- | --- | --- |
| Trials Variable | CHARM-Added/Alternative N=1,584 | EMPHASIS-HF N=2,586 | ATMOSPHERE N=7,011 | PARADIGM-HF N=8,228 | DAPA-HF N=4,734 | GALACTIC-HF N=8,203 | CHARM-Preserved N=740 | I-Preserve N=3,520 | TOPCAT N=1,568 | PARAGON-HF N=4,066 | FINEARTS-HF N=3,829 |
| Age (years) | 0 | 0 | 0 | 0 | 0 | 0 | 0 | 0 | 0 | 0 | 0 |
| Male -no(%) | 0 | 0 | 0 | 0 | 0 | 0 | 0 | 0 | 0 | 0 | 0 |
| *Region -no(%)* |  |  |  |  |  |  |  |  |  |  |  |
| North America | 0 | 0 | 0 | 0 | 0 | 0 | 0 | 0 | 0 | 0 | 0 |
| Latin and Central America | 0 | 0 | 0 | 0 | 0 | 0 | 0 | 0 | 0 | 0 | 0 |
| Western Europe | 0 | 0 | 0 | 0 | 0 | 0 | 0 | 0 | 0 | 0 | 0 |
| Central and Eastern Europe (including Russia) | 0 | 0 | 0 | 0 | 0 | 0 | 0 | 0 | 0 | 0 | 0 |
| Asia-Pacific and other | 0 | 0 | 0 | 0 | 0 | 0 | 0 | 0 | 0 | 0 | 0 |
| *Race -no(%)* |  |  |  |  |  |  |  |  |  |  |  |
| White | 0 | 0 | 0 | 0 | 0 | 0 | 0 | 0 | 0 | 0 | 0 |
| Black | 0 | 0 | 0 | 0 | 0 | 0 | 0 | 0 | 0 | 0 | 0 |
| Asian | 0 | 0 | 0 | 0 | 0 | 0 | 0 | 0 | 0 | 0 | 0 |
| Other | 0 | 0 | 0 | 0 | 0 | 0 | 0 | 0 | 0 | 0 | 0 |
| *Heart failure characteristics* |  |  |  |  |  |  |  |  |  |  |  |
| New York Heart Association functional class III or IV | 0 | 3 | 1 | 13 | 0 | 0 | 0 | 1 | 4 | 2 | 1 |
| Kansas City Cardiomyopathy Questionnaire Clinical Summary Score | 1,584 | 2,586 | 1,085 | 760 | 298 | 62 | 740 | 3,520 | 38 | 80 | 8 |
| *Physiological measurement* |  |  |  |  |  |  |  |  |  |  |  |
| Body mass index (kg/m^2^) | 8 | 15 | 19 | 0 | 2 | 16 | 2 | 14 | 7 | 1 | 11 |
| Heart rate (beats/min) | 0 | 0 | 2 | 0 | 0 | 0 | 0 | 3 | 3 | 0 | 3 |
| Systolic blood pressure (mmHg) | 0 | 0 | 2 | 0 | 0 | 0 | 0 | 0 | 2 | 0 | 1 |
| Diastolic blood pressure (mmHg) | 1 | 0 | 2 | 0 | 0 | 0 | 0 | 0 | 2 | 0 | 1 |
| Left ventricular ejection fraction (%) | 0 | 0 | 0 | 0 | 0 | 0 | 0 | 3 | 0 | 0 | 8 |
| *Laboratory data* |  |  |  |  |  |  |  |  |  |  |  |
| Potassium (mmol/L) | 0 | 0 | 0 | 0 | 0 | 0 | 0 | 0 | 0 | 0 | 0 |
| Estimated glomerular filtration rate (mL/min/1.73m^2^) | 0 | 3 | 0 | 0 | 0 | 0 | 0 | 0 | 0 | 0 | 0 |
| Baseline NT-proBNP (ng/L) | 1,584 | 2,586 | 662 | 14 | 0 | 3 | 740 | 496 | 1,256 | 1,272 | 117 |
| Hemoglobin (g/dL) | 18 | 60 | 44 | 175 | 35 | 287 | 17 | 56 | 15 | 0 | 210 |
| Sodium (mmol/L) | 1 | 82 | 0 | 7 | 1 | 0 | 0 | 3 | 0 | 2 | 97 |
| Serum creatinine (umol/L) | 0 | 6 | 0 | 0 | 0 | 0 | 0 | 0 | 0 | 0 | 0 |
| *Comorbidities* |  |  |  |  |  |  |  |  |  |  |  |
| Diabetes mellitus | 0 | 0 | 0 | 0 | 0 | 0 | 0 | 0 | 1 | 0 | 0 |
| Hypertension | 0 | 0 | 0 | 0 | 0 | 0 | 0 | 0 | 1 | 0 | 0 |
| Myocardial infarction | 0 | 2 | 0 | 0 | 0 | 0 | 0 | 0 | 1 | 0 | 0 |
| Atrial fibrillation/flutter | 1 | 0 | 0 | 0 | 0 | 0 | 0 | 0 | 1 | 4 | 0 |
| Stroke | 0 | 27 | 0 | 0 | 0 | 0 | 0 | 0 | 1 | 7 | 0 |
| *Drugs and devices* |  |  |  |  |  |  |  |  |  |  |  |
| Angiotensin-converting enzyme inhibitor/angiotensin receptor blocker/angiotensin receptor neprilysin inhibitor | 0 | 14 | 0 | 0 | 0 | 0 | 0 | 0 | 1 | 0 | 0 |
| Beta blocker | 0 | 14 | 0 | 0 | 0 | 0 | 0 | 1 | 1 | 0 | 0 |
| Sodium-glucose cotransporter 2 inhibitor | 0 | 0 | 0 | 0 | 0 | 0 | 0 | 1 | 1 | 0 | 0 |
| Mineralocorticoid receptor antagonist | 0 | 0 | 0 | 0 | 0 | 0 | 0 | 1 | 1 | 0 | 0 |
| Calcium channel blockers | 0 | 0 | 0 | 0 | 0 | 0 | 0 | 1 | 1 | 0 | 0 |
| Anticoagulant agent | 0 | 2,586 | 0 | 0 | 0 | 0 | 0 | 1 | 1 | 0 | 0 |
| Antiplatelet agent | 0 | 14 | 0 | 0 | 0 | 0 | 0 | 1 | 1 | 0 | 0 |
| Diuretics | 0 | 14 | 0 | 0 | 0 | 0 | 0 | 1 | 1 | 0 | 0 |
| Loop diuretics | 0 | 2,586 | 0 | 0 | 0 | 0 | 0 | 1 | 1,568 | 0 | 0 |
| Dose of loop diuretics | 1,584 | 2,586 | 7,011 | 2,889 | 1,128 | 1,408 | 740 | 3,520 | 1,568 | 946 | 863 |
| Thiazide diuretics | 0 | 2,586 | 0 | 0 | 0 | 0 | 0 | 1 | 1,568 | 0 | 0 |
| Implantable cardioverter defibrillator | 0 | 3 | 0 | 0 | 0 | 0 | 0 | 0 | 1 | 0 | 0 |
| Pacemaker | 0 | 9 | 0 | 0 | 0 | 0 | 0 | 0 | 1 | 0 | 0 |
|  |  |  |  |  |  |  |  |  |  |  |  |

**Supplemental Table 3**. **Baseline characteristics according to the baseline potassium levels in patients with HFrEF (GALACTIC-HF, DAPA-HF, and PARADIGM-HF) and HFpEF (FINEARTS-HF and PARAGON-HF)**

| **HFrEF (LVEF≤40%) in GALACTIC-HF, DAPA-HF, and PARADIGM-HF** | **K <3.5** | **K ≥ 3.5-<4.0** | **K ≥ 4.0-<4.5** | **K ≥ 4.5-<5.0** | **K ≥ 5.0-<5.5** | **K ≥ 5.5** | P value for trend |
| --- | --- | --- | --- | --- | --- | --- | --- |
|  | N=322 | N=2,003 | N=7,006 | N=7,922 | N=3,141 | N=771 |  |
| Age (years) | 63.0±12.9 | 63.6±12.1 | 63.9±11.8 | 65.0±10.9 | 66.0±10.6 | 65.7±10.6 | <0.001 |
| Male -no(%) | 235 (73.0) | 1,535 (76.6) | 5,438 (77.6) | 6,220 (78.5) | 2,477 (78.9) | 607 (78.7) | 0.006 |
| *Region -no(%)* | | | | | | | <0.001 |
| North America | 75 (23.3) | 348 (17.4) | 966 (13.8) | 881 (11.1) | 292 (9.3) | 69 (9.0) |  |
| Latin and Central America | 66 (20.5) | 394 (19.7) | 1,285 (18.3) | 1,338 (16.9) | 561 (17.9) | 157 (20.4) |  |
| Western Europe | 46 (14.3) | 287 (14.3) | 1,237 (17.7) | 1,522 (19.2) | 547 (17.4) | 109 (14.1) |  |
| Central and Eastern Europe (including Russia) | 52 (16.2) | 458 (22.9) | 2,054 (29.3) | 2,844 (35.9) | 1,291 (41.1) | 349 (45.3) |  |
| Asia-Pacific and other | 83 (25.8) | 516 (25.8) | 1,464 (20.9) | 1,337 (16.9) | 450 (14.3) | 87 (11.3) |  |
| *Race -no(%)* | | | | | | | <0.001 |
| White | 175 (54.4) | 1,183 (59.1) | 4,758 (67.9) | 5,940 (74.5) | 2,464 (78.5) | 626 (81.2) |  |
| Black | 46 (14.3) | 187 (9.3) | 435 (6.2) | 390 (4.9) | 117 (3.7) | 29 (3.8) |  |
| Asian | 66 (20.5) | 450 (22.5) | 1,278 (18.2) | 1,109 (14.0) | 347 (11.1) | 73 (9.5) |  |
| Other | 35 (10.9) | 183 (9.2) | 535 (7.6) | 519 (6.6) | 213 (6.8) | 43 (5.6) |  |
| *Heart failure characteristics* | | | | | | | |
| New York Heart Association functional class III or IV | 125 (38.8) | 738 (36.8) | 2,330 (33.3) | 2,745 (34.7) | 1,151 (36.7) | 329 (42.8) | 0.008 |
| KCCQ-CSS | 66.7 (46.5-83.3) | 70.8 (50.5-87.3) | 74.5 (56.3-88.9) | 75.0 (56.3-89.6) | 73.4 (54.2-87.5) | 68.2 (51.6-85.9) | 0.25 |
| *Physiological measurement* | | | | | | | |
| Body mass index (kg/m^2^) | 28.0±6.5 | 28.2±6.4 | 28.3±6.0 | 28.3±5.7 | 28.2±5.7 | 27.7±5.7 | 0.34 |
| Heart rate (beats/min) | 75.2±12.7 | 73.3±12.1 | 72.2±11.8 | 71.9±12.1 | 71.7±12.0 | 72.9±12.6 | <0.001 |
| Systolic blood pressure (mmHg) | 119.6±17.5 | 119.2±16.3 | 119.9±16.2 | 119.4±15.4 | 119.4±15.1 | 119.1±15.1 | 0.76 |
| Diastolic blood pressure (mmHg) | 72.9±10.8 | 73.0±10.8 | 72.9±10.5 | 72.7±10.1 | 72.6±9.9 | 72.3±10.2 | 0.10 |
| Left ventricular ejection fraction (%) | 27.8±7.3 | 28.3±7.0 | 28.6±6.7 | 28.8±6.5 | 28.9±6.5 | 29.2±6.4 | <0.001 |
| *Laboratory data* | | | | | | | |
| Potassium (mmol/L) | 3.2±0.2 | 3.8±0.1 | 4.2±0.1 | 4.7±0.1 | 5.2±0.1 | 5.8±0.3 |  |
| Estimated glomerular filtration rate (mL/min/1.73m^2^) * | 65.9±29.9 | 67.2±22.1 | 67.6±21.4 | 63.9±19.6 | 59.2±19.4 | 55.2±20.6 | <0.001 |
| Estimated glomerular filtration rate <60 mL/min/1.73m^2^* | 143 (44.4) | 772 (38.5) | 2,619 (37.4) | 3,488 (44.0) | 1,717 (54.7) | 474 (61.5) | <0.001 |
| Baseline NT-proBNP (ng/L) | 2,588 (1,158-5,640) | 1,939 (1,015-4,131) | 1,654 (886-3,340) | 1,597 (868-3,133) | 1,776 (957-3,535) | 2,012 (1,139-4,061) | 0.12 |
| Hemoglobin (g/dL) | 13.4±1.9 | 13.6±1.7 | 13.8±1.7 | 13.8±1.7 | 13.8±1.7 | 13.6±1.8 | 0.015 |
| Sodium (mmol/L) | 140.8±4.3 | 140.7±3.3 | 140.6±3.2 | 140.5±3.2 | 140.2±3.6 | 139.6±4.2 | <0.001 |
| Serum creatinine (umol/L) | 109.0±40.0 | 104.5±36.2 | 101.8±31.7 | 106.4±32.5 | 113.8±35.0 | 125.7±48.9 | <0.001 |
| *Comorbidities* | | | | | | | |
| Diabetes mellitus | 124 (38.5) | 713 (35.6) | 2,527 (36.1) | 3,122 (39.4) | 1,341 (42.7) | 359 (46.6) | <0.001 |
| Hypertension | 235 (73.0) | 1,453 (72.5) | 4,901 (70.0) | 5,587 (70.5) | 2,327 (74.1) | 589 (76.4) | 0.003 |
| Myocardial infarction | 118 (36.7) | 771 (38.5) | 2,849 (40.7) | 3,488 (44.0) | 1,453 (46.3) | 373 (48.4) | <0.001 |
| Atrial fibrillation/flutter | 144 (44.7) | 809 (40.4) | 2,721 (38.8) | 3,098 (39.1) | 1,232 (39.2) | 297 (38.5) | 0.24 |
| Stroke | 30 (9.3) | 181 (9.0) | 626 (8.9) | 725 (9.2) | 295 (9.4) | 70 (9.1) | 0.62 |
| *Drugs and devices* | | | | | | | |
| Angiotensin-converting enzyme inhibitor/angiotensin receptor blocker/angiotensin receptor neprilysin inhibitor | 268 (83.2) | 1,819 (90.8) | 6,557 (93.6) | 7,455 (94.1) | 2,985 (95.0) | 713 (92.5) | <0.001 |
| Beta blocker | 305 (94.7) | 1,853 (92.5) | 6,593 (94.1) | 7,499 (94.7) | 2,969 (94.5) | 720 (93.4) | 0.050 |
| Sodium-glucose cotransporter 2 inhibitor | 4 (1.2) | 14 (0.7) | 72 (1.0) | 81 (1.0) | 42 (1.3) | 4 (0.5) | 0.39 |
| Mineralocorticoid receptor antagonist | 186 (57.8) | 1,198 (59.8) | 4,542 (64.8) | 5,593 (70.6) | 2,239 (71.3) | 572 (74.2) | <0.001 |
| Calcium channel blockers | 43 (13.4) | 208 (10.4) | 668 (9.5) | 613 (7.7) | 261 (8.3) | 62 (8.0) | <0.001 |
| Anticoagulant agent | 185 (57.5) | 1,045 (52.2) | 3,724 (53.2) | 4,304 (54.3) | 1,760 (56.0) | 449 (58.2) | 0.001 |
| Antiplatelet agent | 167 (51.9) | 1,029 (51.4) | 3,707 (52.9) | 4,296 (54.2) | 1,773 (56.5) | 431 (55.9) | <0.001 |
| Diuretics | 301 (93.5) | 1,804 (90.1) | 6,091 (86.9) | 6,805 (85.9) | 2,692 (85.7) | 677 (87.8) | <0.001 |
| Loop diuretics | 277 (86.0) | 1,698 (84.8) | 5,692 (81.2) | 6,388 (80.6) | 2,531 (80.6) | 638 (82.8) | 0.002 |
| Dose of oral loop diuretics (Furosemide equivalent dose) (mg/day) | 80 (40-160) | 40 (40-80) | 40 (40-80) | 40 (40-80) | 40 (40-80) | 40 (40-80) | <0.001 |
| Thiazide diuretics | 25 (7.8) | 165 (8.2) | 439 (6.3) | 416 (5.3) | 154 (4.9) | 40 (5.2) | <0.001 |
| Implantable cardioverter defibrillator | 89 (27.6) | 485 (24.2) | 1,670 (23.8) | 1,939 (24.5) | 718 (22.9) | 156 (20.2) | 0.033 |
| Pacemaker | 23 (7.1) | 203 (10.1) | 703 (10.0) | 759 (9.6) | 315 (10.0) | 65 (8.4) | 0.64 |

| **HFpEF (LVEF≥50%) in FINEARTS-HF and PARAGON-HF** | **K <3.5** | **K ≥ 3.5-<4.0** | **K ≥ 4.0-<4.5** | **K ≥ 4.5-<5.0** | **K ≥ 5.0-<5.5** | **K ≥ 5.5** | P value for trend |
| --- | --- | --- | --- | --- | --- | --- | --- |
|  | N=163 | N=992 | N=3,008 | N=2,797 | N=830 | N=105 |  |
| Age (years) | 73.8±9.9 | 73.7±8.8 | 73.0±8.8 | 73.4±8.5 | 72.9±8.3 | 72.7±8.7 | 0.15 |
| Male -no(%) | 65 (39.9) | 464 (46.8) | 1,352 (45.0) | 1,273 (45.5) | 417 (50.2) | 57 (54.3) | 0.022 |
| *Region -no(%)* | | | | | | | 0.80 |
| North America | 20 (12.3) | 121 (12.2) | 336 (11.2) | 285 (10.2) | 84 (10.1) | 10 (9.5) |  |
| Latin and Central America | 16 (9.8) | 89 (9.0) | 254 (8.4) | 252 (9.0) | 62 (7.5) | 13 (12.4) |  |
| Western Europe | 47 (28.8) | 299 (30.1) | 785 (26.1) | 722 (25.8) | 224 (27.0) | 15 (14.3) |  |
| Central and Eastern Europe (including Russia) | 42 (25.8) | 242 (24.4) | 1,069 (35.5) | 1,124 (40.2) | 347 (41.8) | 52 (49.5) |  |
| Asia-Pacific and other | 38 (23.3) | 241 (24.3) | 564 (18.8) | 414 (14.8) | 113 (13.6) | 15 (14.3) |  |
| *Race -no(%)* | | | | | | | <0.001 |
| White | 116 (71.2) | 718 (72.4) | 2,408 (80.1) | 2,346 (83.9) | 722 (87.0) | 87 (82.9) |  |
| Black | 8 (4.9) | 27 (2.7) | 57 (1.9) | 55 (2.0) | 6 (0.7) | 1 (1.0) |  |
| Asian | 33 (20.3) | 207 (20.9) | 442 (14.7) | 301 (10.8) | 71 (8.6) | 13 (12.4) |  |
| Other | 6 (3.7) | 40 (4.0) | 101 (3.4) | 95 (3.4) | 31 (3.7) | 4 (3.8) |  |
| *Heart failure characteristics* | | | | | | | |
| New York Heart Association functional class III or IV | 47 (28.8) | 272 (27.5) | 785 (26.1) | 666 (23.8) | 182 (21.9) | 28 (26.7) | <0.001 |
| KCCQ-CSS | 65.9 (47.4-82.1) | 68.8 (51.6-84.9) | 69.8 (53.1-85.9) | 70.8 (54.2-84.9) | 71.4 (56.3-85.4) | 72.9 (55.2-84.4) | 0.009 |
| *Physiological measurement* | | | | | | | |
| Body mass index (kg/m^2^) | 29.3±5.5 | 30.3±6.1 | 30.3±5.7 | 30.3±5.5 | 30.5±5.2 | 30.3±5.8 | 0.064 |
| Heart rate (beats/min) | 72.4±14.0 | 71.8±12.2 | 70.6±11.9 | 70.4±11.7 | 70.1±12.4 | 70.4±12.9 | 0.002 |
| Systolic blood pressure (mmHg) | 131.9±18.0 | 130.6±16.3 | 130.5±15.3 | 130.7±15.5 | 130.0±15.1 | 129.4±14.9 | 0.25 |
| Diastolic blood pressure (mmHg) | 74.6±11.1 | 74.7±10.9 | 74.7±10.5 | 74.7±10.4 | 73.9±10.6 | 76.3±11.6 | 0.26 |
| Left ventricular ejection fraction (%) | 58.5±6.2 | 58.2±6.4 | 58.3±6.4 | 58.5±6.6 | 58.5±6.3 | 57.6±6.7 | 0.15 |
| *Laboratory data* | | | | | | | |
| Potassium (mmol/L) | 3.3±0.2 | 3.8±0.1 | 4.2±0.1 | 4.7±0.1 | 5.1±0.1 | 5.7±0.3 |  |
| Estimated glomerular filtration rate (mL/min/1.73m^2^) | 61.0±19.7 | 62.8±19.4 | 63.3±19.2 | 60.4±18.6 | 56.5±18.2 | 52.9±18.6 | <0.001 |
| Estimated glomerular filtration rate <60 mL/min/1.73m^2^ | 82 (50.3) | 461 (46.5) | 1,385 (46.0) | 1,480 (52.9) | 519 (62.5) | 72 (68.6) | <0.001 |
| Baseline NT-proBNP (ng/L) | 1,037 (449-1,855) | 971 (396-1,870) | 710 (332-1,495) | 729 (322-1,407) | 672 (310-1,379) | 826 (348-1,578) | <0.001 |
| Hemoglobin (g/dL) | 13.0±1.6 | 13.4±1.6 | 13.4±1.6 | 13.3±1.6 | 13.3±1.7 | 13.5±1.8 | 0.55 |
| Sodium (mmol/L) | 141.3±4.4 | 141.2±2.9 | 141.0±2.8 | 140.7±3.0 | 140.4±3.3 | 140.3±3.8 | <0.001 |
| Serum creatinine (umol/L) | 97.0±28.9 | 95.9±29.8 | 94.6±27.8 | 98.7±29.0 | 106.2±30.3 | 115.2±33.9 | <0.001 |
| *Comorbidities* | | | | | | | |
| Diabetes mellitus | 66 (40.5) | 388 (39.1) | 1,209 (40.2) | 1,192 (42.6) | 406 (48.9) | 58 (55.2) | <0.001 |
| Hypertension | 150 (92.0) | 930 (93.8) | 2,811 (93.5) | 2,595 (92.8) | 782 (94.2) | 99 (94.3) | 0.89 |
| Myocardial infarction | 23 (14.1) | 158 (15.9) | 599 (19.9) | 564 (20.2) | 183 (22.1) | 25 (23.8) | <0.001 |
| Atrial fibrillation/flutter | 106 (65.0) | 621 (62.7) | 1,728 (57.5) | 1,499 (53.6) | 433 (52.2) | 55 (52.4) | <0.001 |
| Stroke | 27 (16.7) | 152 (15.3) | 340 (11.3) | 321 (11.5) | 85 (10.2) | 22 (21.0) | 0.021 |
| *Drugs and devices* | | | | | | | |
| Angiotensin-converting enzyme inhibitor/angiotensin receptor blocker/angiotensin receptor neprilysin inhibitor | 122 (74.9) | 826 (83.3) | 2,649 (88.1) | 2,522 (90.2) | 785 (94.6) | 96 (91.4) | <0.001 |
| Beta blocker | 118 (72.4) | 804 (81.1) | 2,413 (80.2) | 2,300 (82.2) | 675 (81.3) | 78 (74.3) | 0.14 |
| Sodium-glucose cotransporter 2 inhibitor | 19 (11.7) | 84 (8.5) | 201 (6.7) | 153 (5.5) | 39 (4.7) | 9 (8.6) | <0.001 |
| Mineralocorticoid receptor antagonist | 14 (8.6) | 74 (7.5) | 281 (9.3) | 405 (14.5) | 178 (21.5) | 23 (21.9) | <0.001 |
| Calcium channel blockers | 66 (40.5) | 417 (42.0) | 1,116 (37.1) | 995 (35.6) | 297 (35.8) | 32 (30.5) | <0.001 |
| Anticoagulant agent | 88 (54.0) | 473 (47.7) | 1,285 (42.7) | 1,075 (38.4) | 315 (38.0) | 36 (34.3) | <0.001 |
| Antiplatelet agent | 15 (9.2) | 115 (11.6) | 366 (12.2) | 340 (12.2) | 96 (11.6) | 14 (13.3) | 0.58 |
| Diuretics | 158 (96.9) | 970 (97.8) | 2,927 (97.3) | 2,723 (97.4) | 807 (97.2) | 100 (95.2) | 0.42 |
| Loop diuretics | 128 (78.5) | 814 (82.1) | 2,422 (80.5) | 2,295 (82.1) | 681 (82.1) | 82 (78.1) | 0.48 |
| Dose of oral loop diuretics (Furosemide equivalent dose) (mg/day) | 40 (40-80) | 40 (40-80) | 40 (20-60) | 40 (20-40) | 40 (20-40) | 40 (40-40) | <0.001 |
| Thiazide diuretics | 42 (25.8) | 176 (17.7) | 488 (16.2) | 415 (14.8) | 115 (13.9) | 15 (14.3) | <0.001 |
| Implantable cardioverter defibrillator | 0 (0) | 5 (0.5) | 10 (0.3) | 7 (0.3) | 3 (0.4) | 1 (1.0) | 0.94 |
| Pacemaker | 11 (6.8) | 71 (7.2) | 224 (7.5) | 212 (7.6) | 74 (8.9) | 9 (8.6) | 0.17 |

Abbreviations: HFpEF, heart failure with preserved ejection fraction; KCCQ-CSS, Kansas City Cardiomyopathy Questionnaire Clinical Summary Score; LVEF, left ventricular ejection fraction; NT-proBNP, N-terminal pro-B-type natriuretic peptide. Values are mean ± standard deviation, n (%), or median (interquartile range).

**Supplemental Table 4**. **Two-stage meta-analysis of all-cause death across the HFrEF studies and stratified hazard ratio.**

|  | **ATMOSPHERE** | **CHARM-Added/Alternative** | **DAPA-HF** | **EMPHASIS-HF** | **GALACTIC-HF** | **PARADIGM-HF** | **Overall (Two stage meta-analysis)** | **Stratified hazard ratios (95% CI)** |
| --- | --- | --- | --- | --- | --- | --- | --- | --- |
| **All-cause death** | | | | | | | | |
| K <3.5 | 1.81 (1.25-2.60) | 1.09 (0.56-2.13) | 1.86 (1.14-3.06) | 1.03 (0.52-2.02) | 1.98 (1.51-2.60) | 2.50 (1.81-3.45) | 1.83 (1.47-2.27) | 1.86 (1.58-2.18) |
| K ≥ 3.5-<4.0 | 1.07 (0.91-1.25) | 1.23 (0.91-1.67) | 1.25 (0.95-1.64) | 1.29 (0.97-1.72) | 1.29 (1.11-1.50) | 1.15 (0.96-1.37) | 1.19 (1.10-1.29) | 1.19 (1.10-1.29) |
| K ≥ 4.0-<4.5 | Reference | Reference | Reference | Reference | Reference | Reference | Reference | Reference |
| K ≥ 4.5-<5.0 | 1.04 (0.94-1.16) | 1.07 (0.86-1.32) | 1.03 (0.85-1.25) | 1.05 (0.82-1.33) | 0.90 (0.81-1.00) | 0.96 (0.85-1.09) | 0.99 (0.92-1.06) | 0.98 (0.93-1.04) |
| K ≥ 5.0-<5.5 | 1.07 (0.92-1.25) | 1.05 (0.74-1.49) | 1.01 (0.77-1.30) | 0.88 (0.49-1.59) | 1.03 (0.90-1.17) | 1.16 (1.00-1.36) | 1.07 (0.99-1.15) | 1.07 (0.99-1.16) |
| K ≥ 5.5 | 1.34 (0.99-1.81) | 0.83 (0.39-1.76) | 1.69 (1.18-2.44) | 1.59 (0.39-6.42) | 1.21 (0.99-1.47) | 1.49 (1.11-2.01) | 1.33 (1.16-1.53) | 1.32 (1.16-1.51) |

HRs with 95% confidence intervals for all-cause death were estimated using potassium ≥ 4.0-<4.5 mmol/L as the reference category within each trial. In the Cox proportional hazard models, each HR was adjusted with the treatment assignment. Stratified HR of the pooled dataset was stratified according to the trial and adjusted with the treatment assignment.

Abbreviations: CI, confidence interval; HFrEF, heart failure with reduced ejection fraction; HR, hazard ratio; K, potassium.

**Supplemental Table 5**. **Clinical outcomes according to the baseline potassium levels in patients with HFrEF (GALACTIC-HF, DAPA-HF, and PARADIGM-HF) and HFpEF (FINEARTS-HF and PARAGON-HF)**

| **HFrEF (LVEF≤40%) in GALACTIC, DAPA, and PARADIGM-HF** | **Crude incidence rate, 100 patients-year (95% CI)** | **Unadjusted hazard ratios (95% CI)*** | **Adjusted hazard ratios without dose of oral loop diuretics (95% CI)**** | **Adjusted hazard ratios with dose of oral loop diuretics (95% CI)***** |
| --- | --- | --- | --- | --- |
| **All-cause death** | | | | |
| K < 3.5 (n=322) | 22.1 (18.4-26.6) | 2.13 (1.76-2.58) | 1.63 (1.34-1.99) | 1.60 (1.31-1.95) |
| K ≥ 3.5-<4.0 (n=2,003) | 12.4 (11.3-13.6) | 1.23 (1.10-1.36) | 1.07 (0.96-1.19) | 1.05 (0.94-1.17) |
| K ≥ 4.0-<4.5 (n=7,006) | 10.2 (9.7-10.7) | Reference | Reference | Reference |
| K ≥ 4.5-<5.0 (n=7,922) | 9.7 (9.2-10.2) | 0.94 (0.87-1.01) | 0.93 (0.86-1.00) | 0.94 (0.87-1.01) |
| K ≥ 5.0-<5.5 (n=3,141) | 11.3 (10.4-12.2) | 1.07 (0.98-1.18) | 0.99 (0.90-1.10) | 1.01 (0.92-1.11) |
| K ≥ 5.5 (n=771) | 14.9 (13.0-17.2) | 1.34 (1.16-1.56) | 1.07 (0.92-1.25) | 1.09 (0.94-1.27) |
| **Cardiovascular death** | | | | |
| K < 3.5 | 18.6 (15.2-22.8) | 2.28 (1.85-2.81) | 1.74 (1.40-2.15) | 1.70 (1.37-2.11) |
| K ≥ 3.5-<4.0 | 10.2 (9.2-11.3) | 1.28 (1.14-1.44) | 1.11 (0.98-1.25) | 1.09 (0.97-1.23) |
| K ≥ 4.0-<4.5 | 8.0 (7.5-8.5) | Reference | Reference | Reference |
| K ≥ 4.5-<5.0 | 7.5 (7.1-8.0) | 0.93 (0.86-1.02) | 0.93 (0.85-1.01) | 0.94 (0.86-1.02) |
| K ≥ 5.0-<5.5 | 8.8 (8.0-9.5) | 1.07 (0.96-1.19) | 0.99 (0.89-1.11) | 1.01 (0.90-1.12) |
| K ≥ 5.5 | 11.0 (9.4-12.9) | 1.28 (1.07-1.52) | 1.02 (0.86-1.22) | 1.04 (0.87-1.24) |
| **Non-cardiovascular death** | | | | |
| K < 3.5 | 2.9 (1.8-4.9) | 1.74 (1.03-2.94) | 1.35 (0.77-2.37) | 1.31 (0.75-2.31) |
| K ≥ 3.5-<4.0 | 1.6 (1.2-2.1) | 0.97 (0.73-1.30) | 0.90 (0.67-1.21) | 0.88 (0.66-1.19) |
| K ≥ 4.0-<4.5 | 1.7 (1.5-1.9) | Reference | Reference | Reference |
| K ≥ 4.5-<5.0 | 1.6 (1.4-1.8) | 0.94 (0.79-1.13) | 0.91 (0.76-1.10) | 0.93 (0.77-1.12) |
| K ≥ 5.0-<5.5 | 1.8 (1.5-2.1) | 1.03 (0.82-1.30) | 0.94 (0.74-1.19) | 0.96 (0.75-1.22) |
| K ≥ 5.5 | 2.8 (2.1-3.9) | 1.54 (1.09-2.17) | 1.20 (0.84-1.72) | 1.23 (0.86-1.76) |
| **Sudden death** | | | | |
| K < 3.5 | 5.1 (3.5-7.5) | 1.80 (1.21-2.68) | 1.56 (1.05-2.33) | 1.56 (1.04-2.33) |
| K ≥ 3.5-<4.0 | 3.2 (2.6-3.8) | 1.12 (0.91-1.37) | 1.00 (0.81-1.24) | 1.00 (0.81-1.23) |
| K ≥ 4.0-<4.5 | 2.8 (2.6-3.1) | Reference | Reference | Reference |
| K ≥ 4.5-<5.0 | 2.7 (2.4-2.9) | 0.94 (0.82-1.09) | 0.94 (0.81-1.09) | 0.94 (0.81-1.09) |
| K ≥ 5.0-<5.5 | 2.6 (2.3-3.1) | 0.94 (0.78-1.14) | 0.90 (0.74-1.09) | 0.90 (0.74-1.09) |
| K ≥ 5.5 | 3.6 (2.8-4.8) | 1.32 (0.98-1.78) | 1.17 (0.86-1.59) | 1.17 (0.87-1.59) |
| **Pump failure death** | | | | |
| K < 3.5 | 9.6 (7.3-12.7) | 3.08 (2.29-4.14) | 2.02 (1.48-2.74) | 1.97 (1.45-2.67) |
| K ≥ 3.5-<4.0 | 4.4 (3.7-5.1) | 1.50 (1.25-1.80) | 1.18 (0.98-1.43) | 1.16 (0.96-1.40) |
| K ≥ 4.0-<4.5 | 3.0 (2.7-3.3) | Reference | Reference | Reference |
| K ≥ 4.5-<5.0 | 2.7 (2.4-3.0) | 0.88 (0.77-1.01) | 0.88 (0.76-1.01) | 0.89 (0.77-1.03) |
| K ≥ 5.0-<5.5 | 3.2 (2.8-3.7) | 1.02 (0.86-1.21) | 0.95 (0.80-1.14) | 0.98 (0.82-1.17) |
| K ≥ 5.5 | 4.1 (3.1-5.3) | 1.16 (0.87-1.54) | 0.94 (0.71-1.26) | 0.97 (0.72-1.29) |
| **First heart failure hospitalization** | | | | |
| K < 3.5 | 22.9 (18.8-27.9) | 1.93 (1.57-2.37) | 1.30 (1.05-1.61) | 1.25 (1.01-1.55) |
| K ≥ 3.5-<4.0 | 14.7 (13.4-16.0) | 1.33 (1.20-1.48) | 1.11 (1.00-1.24) | 1.08 (0.97-1.20) |
| K ≥ 4.0-<4.5 | 11.1 (10.5-11.7) | Reference | Reference | Reference |
| K ≥ 4.5-<5.0 | 10.2 (9.6-10.7) | 0.90 (0.83-0.97) | 0.88 (0.82-0.96) | 0.90 (0.83-0.97) |
| K ≥ 5.0-<5.5 | 11.1 (10.2-12.0) | 0.95 (0.86-1.05) | 0.91 (0.82-1.01) | 0.93 (0.84-1.03) |
| K ≥ 5.5 | 12.9 (11.0-15.1) | 1.00 (0.84-1.18) | 0.84 (0.71-1.00) | 0.85 (0.72-1.01) |
| **First heart failure hospitalization or all-cause death** | | | | |
| K < 3.5 | 38.0 (32.6-44.4) | 2.05 (1.75-2.41) | 1.49 (1.26-1.76) | 1.44 (1.22-1.69) |
| K ≥ 3.5-<4.0 | 22.6 (21.0-24.3) | 1.28 (1.17-1.39) | 1.09 (1.00-1.19) | 1.07 (0.98-1.17) |
| K ≥ 4.0-<4.5 | 17.7 (17.0-18.5) | Reference | Reference | Reference |
| K ≥ 4.5-<5.0 | 16.7 (16.0-17.4) | 0.93 (0.87-0.98) | 0.91 (0.86-0.97) | 0.92 (0.87-0.98) |
| K ≥ 5.0-<5.5 | 18.9 (17.8-20.1) | 1.03 (0.95-1.11) | 0.97 (0.90-1.05) | 0.99 (0.91-1.07) |
| K ≥ 5.5 | 24.2 (21.5-27.1) | 1.22 (1.08-1.38) | 1.00 (0.88-1.14) | 1.01 (0.89-1.15) |
| **First heart failure hospitalization or cardiovascular death** | | | | |
| K < 3.5 | 34.2 (29.1-40.3) | 2.03 (1.72-2.41) | 1.46 (1.23-1.74) | 1.41 (1.19-1.68) |
| K ≥ 3.5-<4.0 | 20.7 (19.2-22.4) | 1.30 (1.19-1.42) | 1.11 (1.01-1.21) | 1.08 (0.99-1.18) |
| K ≥ 4.0-<4.5 | 16.0 (15.3-16.7) | Reference | Reference | Reference |
| K ≥ 4.5-<5.0 | 15.0 (14.3-15.6) | 0.92 (0.86-0.98) | 0.91 (0.85-0.97) | 0.92 (0.86-0.98) |
| K ≥ 5.0-<5.5 | 16.8 (15.7-17.9) | 1.01 (0.93-1.09) | 0.96 (0.88-1.04) | 0.97 (0.90-1.06) |
| K ≥ 5.5 | 20.4 (18.0-23.1) | 1.14 (0.99-1.30) | 0.94 (0.82-1.08) | 0.95 (0.83-1.09) |

| **HFpEF (LVEF≥50%) in FINEARTS-HF and PARAGON-HF** | **Crude incidence rate, 100 patients-year (95% CI)** | **Unadjusted hazard ratios (95% CI)*** | **Adjusted hazard ratios  without dose of oral loop diuretics (95% CI)**** | **Adjusted hazard ratios  with dose of oral loop diuretics (95% CI)***** |
| --- | --- | --- | --- | --- |
| **All-cause death** | | | | |
| K < 3.5 (n=163) | 7.9 (5.6-11.2) | 1.49 (1.04-2.13) | 1.39 (0.97-2.00) | 1.36 (0.95-1.96) |
| K ≥ 3.5-<4.0 (n=992) | 6.6 (5.7-7.7) | 1.29 (1.08-1.54) | 1.20 (0.99-1.45) | 1.18 (0.98-1.42) |
| K ≥ 4.0-<4.5 (n=3,008) | 5.0 (4.5-5.5) | Reference | Reference | Reference |
| K ≥ 4.5-<5.0 (n=2,797) | 5.4 (4.9-5.9) | 1.10 (0.96-1.26) | 1.08 (0.94-1.24) | 1.10 (0.95-1.26) |
| K ≥ 5.0-<5.5 (n=830) | 5.9 (5.0-7.0) | 1.26 (1.04-1.54) | 1.19 (0.97-1.46) | 1.23 (1.01-1.51) |
| K ≥ 5.5 (n=105) | 9.5 (6.4-14.1) | 2.01 (1.34-3.02) | 1.63 (1.08-2.46) | 1.67 (1.10-2.52) |
| **Cardiovascular death** | | | | |
| K < 3.5 | 4.0 (2.4-6.5) | 1.63 (0.98-2.71) | 1.62 (0.97-2.72) | 1.60 (0.95-2.68) |
| K ≥ 3.5-<4.0 | 3.7 (3.1-4.6) | 1.56 (1.22-1.99) | 1.51 (1.17-1.94) | 1.48 (1.15-1.90) |
| K ≥ 4.0-<4.5 | 2.4 (2.1-2.8) | Reference | Reference | Reference |
| K ≥ 4.5-<5.0 | 2.6 (2.3-3.0) | 1.09 (0.90-1.33) | 1.09 (0.89-1.34) | 1.11 (0.90-1.36) |
| K ≥ 5.0-<5.5 | 3.6 (2.9-4.5) | 1.56 (1.20-2.02) | 1.49 (1.14-1.95) | 1.55 (1.18-2.02) |
| K ≥ 5.5 | 4.6 (2.6-8.0) | 1.96 (1.10-3.52) | 1.51 (0.83-2.72) | 1.54 (0.85-2.78) |
| **Non-cardiovascular death** | | | | |
| K < 3.5 | 3.5 (2.1-5.8) | 1.57 (0.91-2.72) | 1.43 (0.82-2.49) | 1.41 (0.81-2.45) |
| K ≥ 3.5-<4.0 | 2.3 (1.8-3.0) | 1.11 (0.82-1.49) | 0.98 (0.71-1.35) | 0.97 (0.71-1.34) |
| K ≥ 4.0-<4.5 | 2.0 (1.7-2.3) | Reference | Reference | Reference |
| K ≥ 4.5-<5.0 | 2.2 (1.9-2.5) | 1.13 (0.91-1.40) | 1.10 (0.88-1.37) | 1.11 (0.89-1.39) |
| K ≥ 5.0-<5.5 | 1.8 (1.3-2.5) | 1.00 (0.71-1.41) | 0.91 (0.63-1.30) | 0.93 (0.65-1.33) |
| K ≥ 5.5 | 3.0 (1.5-6.1) | 1.64 (0.81-3.34) | 1.51 (0.73-3.10) | 1.54 (0.75-3.16) |
| **Sudden death** | | | | |
| K < 3.5 | 0.2 (0.0-1.8) | 0.28 (0.04-2.04) | 0.28 (0.04-2.01) | 0.28 (0.04-2.03) |
| K ≥ 3.5-<4.0 | 1.1 (0.7-1.5) | 1.26 (0.81-1.97) | 1.19 (0.75-1.90) | 1.18 (0.74-1.88) |
| K ≥ 4.0-<4.5 | 0.8 (0.6-1.0) | Reference | Reference | Reference |
| K ≥ 4.5-<5.0 | 1.1 (0.9-1.4) | 1.38 (1.00-1.91) | 1.38 (0.99-1.92) | 1.39 (1.00-1.93) |
| K ≥ 5.0-<5.5 | 1.3 (0.9-1.9) | 1.69 (1.09-2.62) | 1.60 (1.01-2.51) | 1.62 (1.03-2.56) |
| K ≥ 5.5 | 1.9 (0.8-4.6) | 2.45 (0.99-6.08) | 1.98 (0.79-4.98) | 2.02 (0.80-5.09) |
| **Pump failure death** | | | | |
| K < 3.5 | 2.2 (1.2-4.3) | 2.39 (1.19-4.80) | 2.42 (1.19-4.94) | 2.37 (1.16-4.83) |
| K ≥ 3.5-<4.0 | 1.2 (0.9-1.8) | 1.38 (0.91-2.09) | 1.29 (0.83-2.00) | 1.25 (0.81-1.93) |
| K ≥ 4.0-<4.5 | 0.9 (0.7-1.1) | Reference | Reference | Reference |
| K ≥ 4.5-<5.0 | 0.7 (0.6-1.0) | 0.85 (0.60-1.21) | 0.88 (0.61-1.26) | 0.91 (0.63-1.32) |
| K ≥ 5.0-<5.5 | 1.0 (0.7-1.5) | 1.22 (0.76-1.95) | 1.22 (0.75-1.99) | 1.31 (0.80-2.13) |
| K ≥ 5.5 | 1.9 (0.8-4.6) | 2.30 (0.93-5.70) | 1.38 (0.54-3.56) | 1.41 (0.55-3.64) |
| **First heart failure hospitalization** | | | | |
| K < 3.5 | 8.3 (5.8-11.8) | 1.26 (0.88-1.82) | 1.22 (0.85-1.76) | 1.17 (0.81-1.69) |
| K ≥ 3.5-<4.0 | 8.0 (6.9-9.2) | 1.23 (1.04-1.46) | 1.15 (0.96-1.37) | 1.12 (0.94-1.34) |
| K ≥ 4.0-<4.5 | 6.4 (5.9-7.0) | Reference | Reference | Reference |
| K ≥ 4.5-<5.0 | 6.3 (5.7-6.9) | 0.98 (0.86-1.12) | 1.00 (0.87-1.14) | 1.03 (0.90-1.17) |
| K ≥ 5.0-<5.5 | 7.0 (5.9-8.2) | 1.11 (0.92-1.33) | 1.04 (0.86-1.26) | 1.10 (0.91-1.34) |
| K ≥ 5.5 | 7.1 (4.4-11.4) | 1.10 (0.68-1.79) | 0.84 (0.50-1.41) | 0.87 (0.52-1.47) |
| **First heart failure hospitalization or all-cause death** | | | | |
| K < 3.5 | 13.1 (9.9-17.4) | 1.28 (0.95-1.70) | 1.20 (0.90-1.61) | 1.16 (0.87-1.55) |
| K ≥ 3.5-<4.0 | 12.6 (11.3-14.2) | 1.26 (1.10-1.44) | 1.18 (1.02-1.35) | 1.15 (1.00-1.33) |
| K ≥ 4.0-<4.5 | 9.9 (9.2-10.7) | Reference | Reference | Reference |
| K ≥ 4.5-<5.0 | 10.1 (9.4-10.9) | 1.03 (0.93-1.15) | 1.03 (0.92-1.14) | 1.05 (0.95-1.17) |
| K ≥ 5.0-<5.5 | 11.0 (9.7-12.6) | 1.15 (0.99-1.33) | 1.08 (0.93-1.26) | 1.14 (0.98-1.33) |
| K ≥ 5.5 | 14.2 (10.1-19.9) | 1.45 (1.03-2.05) | 1.16 (0.81-1.66) | 1.19 (0.83-1.70) |
| **First heart failure hospitalization or cardiovascular death** | | | | |
| K < 3.5 | 10.2 (7.4-14.0) | 1.26 (0.91-1.75) | 1.21 (0.87-1.69) | 1.17 (0.84-1.63) |
| K ≥ 3.5-<4.0 | 10.4 (9.1-11.8) | 1.30 (1.12-1.52) | 1.23 (1.05-1.44) | 1.20 (1.03-1.41) |
| K ≥ 4.0-<4.5 | 7.9 (7.3-8.5) | Reference | Reference | Reference |
| K ≥ 4.5-<5.0 | 7.8 (7.2-8.5) | 1.00 (0.89-1.12) | 1.00 (0.89-1.13) | 1.03 (0.91-1.16) |
| K ≥ 5.0-<5.5 | 9.2 (8.0-10.6) | 1.19 (1.01-1.40) | 1.13 (0.95-1.34) | 1.19 (1.01-1.42) |
| K ≥ 5.5 | 10.0 (6.7-14.9) | 1.27 (0.84-1.91) | 0.99 (0.64-1.53) | 1.03 (0.67-1.59) |

Abbreviations: CI, confidence interval; HFpEF, heart failure with preserved ejection fraction; HFrEF, heart failure with reduced ejection fraction; and LVEF, left ventricular ejection fraction.

*****Models were stratified according to the trial and with the treatment assignment as covariate.

******Models were stratified according to the trial and with the treatment assignment as covariate, and adjusted for age, sex, region, race, heart rate, New York Heart Association functional classes III or IV, systolic blood pressure, body mass index, left ventricular ejection fraction, serum creatinine, hemoglobin, NT-proBNP (log-transformed), history of atrial fibrillation, history of myocardial infarction, history of diabetes, diuretics use, mineralocorticoid receptor antagonist use, and angiotensin-converting enzyme inhibitor/angiotensin receptor blocker/angiotensin receptor neprilysin inhibitor use.

******* Models were stratified trial and adjusted for above variables and dose of oral loop diuretics.

In the cases with missing data, the missing indicator method was used for NT-proBNP values and dose of oral loop diuretics.

**Supplemental Table 6**. **Comparison of models with and without interaction terms for serum potassium levels**

| **HFrEF** | | | | | | | |
| --- | --- | --- | --- | --- | --- | --- | --- |
|  | P for interaction between serum potassium levels and treatment assignment in incidence rate | P for interaction between serum potassium levels and MRA use in incidence rate | Serum potassium level at the nadir of the incidence rate  MRA (-) | Serum potassium level at the nadir of the incidence rate  MRA (+) | P for interaction between serum potassium levels and eGFR (<60 or ≥60 mL/min/1.73m^2^) in incidence rate | Serum potassium level at the nadir of the incidence rate  eGFR <60 mL/min/1.73m^2^ | Serum potassium level at the nadir of the incidence rate  eGFR ≥60 mL/min/1.73m^2^ |
| All-cause death | 0.80 | 0.014 | 4.4 | 4.5 | <0.001 | 4.8 | 4.3 |
| CV death | 0.94 | 0.029 | 4.4 | 4.5 | 0.022 | 4.8 | 4.4 |
| Sudden death | 0.85 | 0.25 |  |  | 0.14 |  |  |
| Pump failure death | 0.73 | 0.73 |  |  | 0.38 |  |  |
| Non-CV death | 0.77 | 0.11 |  |  | <0.001 | 4.2 | Undefined |
| First HF hospitalization | 0.061 | 0.24 |  |  | 0.073 |  |  |
| First HF hospitalization + All-cause death | 0.061 | 0.12 |  |  | <0.001 | 4.8 | 4.4 |
| First HF hospitalization + CV death | 0.095 | 0.19 |  |  | 0.004 | 4.8 | 4.4 |
|  |  |  |  |  |  |  |  |
| **HFpEF** | | | | | | | |
|  | P for interaction between serum potassium levels and treatment assignment in incidence rate | P for interaction between serum potassium levels and MRA use in incidence rate | Serum potassium level at the nadir of the incidence rate  MRA (-) | Serum potassium level at the nadir of the incidence rate  MRA (+) | P for interaction between serum potassium levels and eGFR (<60 or ≥60 mL/min/1.73m^2^) in incidence rate | Serum potassium level at the nadir of the incidence rate  eGFR <60 mL/min/1.73m^2^ | Serum potassium level at the nadir of the incidence rate  eGFR ≥60 mL/min/1.73m^2^ |
| All-cause death | 0.70 | 0.53 |  |  | 0.43 |  |  |
| CV death | 0.77 | 0.61 |  |  | 0.10 |  |  |
| Sudden death | 0.22 | 0.83 |  |  | 0.14 |  |  |
| Pump failure death | 0.89 | 0.63 |  |  | 0.85 |  |  |
| Non-CV death | 0.68 | 0.44 |  |  | 0.58 |  |  |
| First HF hospitalization | 0.84 | 0.31 |  |  | 0.71 |  |  |
| First HF hospitalization + All-cause death | 0.30 | 0.71 |  |  | 0.97 |  |  |
| First HF hospitalization + CV death | 0.63 | 0.70 |  |  | 0.90 |  |  |

Abbreviations: CV, cardiovascular; eGFR, estimated glomerular filtration rate; HF, heart failure; HFpEF, heart failure with preserved ejection fraction; HFrEF, heart failure with reduced ejection fraction; and MRA, mineralocorticoid receptor antagonist.

P for interaction was assessed using a likelihood ratio test, comparing models with and without the relevant interaction terms for potassium with baseline(eGFR category (<60 vs ≥60 mL/min/1.73 m²), randomized treatment assignment, and use of MRA.

Nadirs of serum potassium levels of interest were calculated only when a statistically significant interaction (P<0.05) was observed.

**Supplemental Table 7**. **Clinical outcomes according to the treatment assignment (control vs intervention) in patients with HFrEF**

| **Controls (n = 15,016)** | | | |  |
| --- | --- | --- | --- | --- |
| **HFrEF (LVEF≤40%)** | **Crude incidence rate, 100 patients-year (95% CI)** | **Unadjusted hazard ratios (95% CI)*** | **Adjusted hazard ratios (95% CI)**** | **P for interaction (adjusted hazard ratios) between treatment assignment and serum potassium levels** |
| **All-cause death** | | | | 0.83 |
| K <3.5 (n=233) | 18.7 (14.9-23.3) | 1.84 (1.46-2.32) | 1.38 (1.08-1.75) |  |
| K ≥ 3.5-<4.0 (n=1,555) | 11.5 (10.4-12.8) | 1.15 (1.02-1.30) | 1.05 (0.93-1.18) |  |
| K ≥ 4.0-<4.5 (n=5,419) | 10.0 (9.5-10.6) | Reference | Reference |  |
| K ≥ 4.5-<5.0 (n=5,552) | 9.7 (9.2-10.3) | 0.95 (0.88-1.03) | 0.93 (0.85-1.01) |  |
| K ≥ 5.0-<5.5 (n=1,897) | 11.5 (10.4-12.6) | 1.09 (0.98-1.22) | 1.00 (0.89-1.12) |  |
| K ≥ 5.5 (n=460) | 14.4 (12.1-17.1) | 1.31 (1.09-1.58) | 1.07 (0.89-1.30) |  |
| **Cardiovascular death** | | | | 0.86 |
| K <3.5 | 16.2 (12.8-20.6) | 1.95 (1.52-2.50) | 1.46 (1.13-1.89) |  |
| K ≥ 3.5-<4.0 | 9.8 (8.8-10.9) | 1.19 (1.05-1.36) | 1.07 (0.94-1.22) |  |
| K ≥ 4.0-<4.5 | 8.2 (7.7-8.8) | Reference | Reference |  |
| K ≥ 4.5-<5.0 | 7.7 (7.2-8.2) | 0.93 (0.85-1.02) | 0.90 (0.82-0.99) |  |
| K ≥ 5.0-<5.5 | 9.2 (8.3-10.2) | 1.09 (0.96-1.23) | 0.99 (0.87-1.12) |  |
| K ≥ 5.5 | 10.8 (8.8-13.2) | 1.23 (1.00-1.53) | 1.01 (0.81-1.25) |  |
| **Non-cardiovascular death** | | | | 0.99 |
| K <3.5 | 2.2 (1.1-4.2) | 1.42 (0.73-2.77) | 1.11 (0.54-2.27) |  |
| K ≥ 3.5-<4.0 | 1.3 (1.0-1.8) | 0.89-0.64-1.25) | 0.88 (0.643-1.24) |  |
| K ≥ 4.0-<4.5 | 1.5 (1.3-1.8) | Reference | Reference |  |
| K ≥ 4.5-<5.0 | 1.8 (1.5-2.0) | 1.13 (0.92-1.38) | 1.07 (0.87-1.31) |  |
| K ≥ 5.0-<5.5 | 1.7 (1.3-2.2) | 1.05 (0.79-1.40) | 0.93 (0.70-1.25) |  |
| K ≥ 5.5 | 2.6 (1.8-4.0) | 1.53 (0.99-2.37) | 1.18 (0.76-1.85) |  |
| **Sudden death** | | | | 0.94 |
| K <3.5 | 5.1 (3.3-7.8) | 1.64 (1.05-2.54) | 1.43 (0.91-2.22) |  |
| K ≥ 3.5-<4.0 | 3.8 (3.2-4.5) | 1.24 (1.01-1.53) | 1.12 (0.91-1.39) |  |
| K ≥ 4.0-<4.5 | 3.1 (2.8-3.4) | Reference | Reference |  |
| K ≥ 4.5-<5.0 | 3.1 (2.8-3.4) | 1.01 (0.87-1.17) | 1.00 (0.86-1.16) |  |
| K ≥ 5.0-<5.5 | 3.0 (2.5-3.6) | 1.02 (0.82-1.25) | 0.99 (0.80-1.22) |  |
| K ≥ 5.5 | 3.7 (2.6-5.2) | 1.26 (0.88-1.81) | 1.14 (0.79-1.65) |  |
| **Pump failure death** | | | | 0.94 |
| K <3.5 | 7.5 (5.3-10.7) | 2.73 (1.8-3.95) | 1.71 (1.16-2.52) |  |
| K ≥ 3.5-<4.0 | 3.8 (3.2-4.6) | 1.44 (1.16-1.77) | 1.22 (0.98-1.52) |  |
| K ≥ 4.0-<4.5 | 2.7 (2.4-3.0) | Reference | Reference |  |
| K ≥ 4.5-<5.0 | 2.5 (2.2-2.8) | 0.89 (0.76-1.05) | 0.86 (0.73-1.02) |  |
| K ≥ 5.0-<5.5 | 3.2 (2.7-3.8) | 1.10 (0.89-1.36) | 0.98 (0.79-1.21) |  |
| K ≥ 5.5 | 3.4 (2.4-4.9) | 1.07 (0.74-1.56) | 0.81 (0.55-1.19) |  |
| **First heart failure hospitalization** | | | | 0.44 |
| K <3.5 | 19.8 (15.6-25.1) | 1.68 (1.32-2.16) | 1.20 (0.92-1.55) |  |
| K ≥ 3.5-<4.0 | 14.1 (12.8-15.6) | 1.29 (1.15-1.45) | 1.14 (1.02-1.29) |  |
| K ≥ 4.0-<4.5 | 10.8 (10.2-11.5) | Reference | Reference |  |
| K ≥ 4.5-<5.0 | 9.7 (9.1-10.3) | 0.88 (0.80-0.95) | 0.86 (0.79-0.94) |  |
| K ≥ 5.0-<5.5 | 10.9 (9.9-12.1) | 0.96 (0.85-1.08) | 0.91 (0.81-1.03) |  |
| K ≥ 5.5 | 13.3 (10.9-16.1) | 1.04 (0.85-1.27) | 0.92 (0.75-1.13) |  |
| **First heart failure hospitalization or all-cause death** | | | | 0.41 |
| K <3.5 | 32.1 (26.6-38.8) | 1.74 (1.43-2.11) | 1.31 (1.07-1.60) |  |
| K ≥ 3.5-<4.0 | 21.8 (20.2-23.7) | 1.23 (1.12-1.35) | 1.10 (1.00-1.21) |  |
| K ≥ 4.0-<4.5 | 17.6 (16.8-18.4) | Reference | Reference |  |
| K ≥ 4.5-<5.0 | 16.6 (15.8-17.3) | 0.92 (0.86-0.98) | 0.90 (0.84-0.97) |  |
| K ≥ 5.0-<5.5 | 18.8 (17.5-20.3) | 1.02 (0.94-1.12) | 0.96 (0.88-1.05) |  |
| K ≥ 5.5 | 24.3 (21.0-28.1) | 1.22 (1.04-1.42) | 1.05 (0.90-1.22) |  |
| **First heart failure hospitalization or cardiovascular death** | | | | 0.33 |
| K <3.5 | 29.2 (24.0-35.5) | 1.72 (1.40-2.10) | 1.29 (1.05-1.59) |  |
| K ≥ 3.5-<4.0 | 20.4 (18.8-22.2) | 1.26 (1.15-1.39) | 1.12 (1.02-1.24) |  |
| K ≥ 4.0-<4.5 | 16.1 (15.3-16.9) | Reference | Reference |  |
| K ≥ 4.5-<5.0 | 14.9 (14.2-15.6) | 0.91 (0.85-0.97) | 0.89 (0.83-0.96) |  |
| K ≥ 5.0-<5.5 | 17.0 (15.6-18.4) | 1.01 (0.92-1.11) | 0.95 (0.87-1.05) |  |
| K ≥ 5.5 | 21.1 (18.1-24.7) | 1.16 (0.99-1.37) | 1.00 (0.85-1.18) |  |
| **Interventions (n = 17,330)** | | | |  |
| **HFrEF (LVEF≤40%)** | **Crude incidence rate, 100 patients-year (95% CI)** | **Unadjusted hazard ratios* (95% CI)** | **Adjusted hazard ratios** (95% CI)** |  |
| **All-cause death** | | | |  |
| K <3.5 (n=269) | 16.9 (13.6-20.9) | 1.87 (1.50-2.33) | 1.62 (1.29-2.02) |  |
| K ≥ 3.5-<4.0 (n=1,746) | 10.7 (9.7-11.8) | 1.22 (1.09-1.37) | 1.09 (0.97-1.22) |  |
| K ≥ 4.0-<4.5 (n=6,192) | 8.8 (8.4-9.3) | Reference | Reference |  |
| K ≥ 4.5-<5.0 (n=6,411) | 9.0 (8.5-9.5) | 1.01 (0.93-1.09) | 0.96 (0.89-1.04) |  |
| K ≥ 5.0-<5.5 (n=2,222) | 9.8 (9.0-10.7) | 1.05 (0.95-1.17) | 0.93 (0.83-1.04) |  |
| K ≥ 5.5 (n=490) | 13.3 (11.2-15.7) | 1.34 (1.12-1.61) | 0.99 (0.82-1.19) |  |
| **Cardiovascular death** | | | |  |
| K <3.5 | 14.6 (11.6-18.4) | 2.01 (1.58-2.54) | 1.70 (1.34-2.16) |  |
| K ≥ 3.5-<4.0 | 9.0 (8.1-10.0) | 1.27 (1.12-1.43) | 1.11 (0.98-1.26) |  |
| K ≥ 4.0-<4.5 | 7.2 (6.8-7.7) | Reference | Reference |  |
| K ≥ 4.5-<5.0 | 7.2 (6.8-7.7) | 0.99 (0.91-1.08) | 0.94 (0.86-1.03) |  |
| K ≥ 5.0-<5.5 | 7.9 (7.1-8.7) | 1.05 (0.94-1.19) | 0.93 (0.82-1.05) |  |
| K ≥ 5.5 | 10.0 (8.2-12.2) | 1.27 (1.03-1.57) | 0.92 (0.74-1.14) |  |
| **Non-cardiovascular death** | | | |  |
| K <3.5 | 1.81 (0.94-3.47) | 1.31 (0.67-2.55) | 1.22 (0.60-2.48) |  |
| K ≥ 3.5-<4.0 | 1.39 (1.06-1.82) | 1.04 (0.77-1.41) | 0.96 (0.70-1.32) |  |
| K ≥ 4.0-<4.5 | 1.36 (1.18-1.56) | Reference | Reference |  |
| K ≥ 4.5-<5.0 | 1.46 (1.27-1.67) | 1.06 (0.87-1.29) | 1.00 (0.82-1.22) |  |
| K ≥ 5.0-<5.5 | 1.50 (1.19-1.89) | 1.04 (0.80-1.37) | 0.92 (0.70-1.22) |  |
| K ≥ 5.5 | 2.57 (1.74-3.80) | 1.68 (1.11-2.56) | 1.33 (0.86-2.06) |  |
| **Sudden death** | | | |  |
| K <3.5 | 5.6 (3.9-8.1) | 2.10 (1.43-3.09) | 1.71 (1.16-2.52) |  |
| K ≥ 3.5-<4.0 | 3.0 (2.5-3.7) | 1.12 (0.91-1.38) | 1.01 (0.82-1.25) |  |
| K ≥ 4.0-<4.5 | 2.7 (2.5-3.0) | Reference | Reference |  |
| K ≥ 4.5-<5.0 | 2.7 (2.4-2.9) | 0.98 (0.85-1.13) | 0.96 (0.83-1.11) |  |
| K ≥ 5.0-<5.5 | 2.6 (2.1-3.1) | 0.96 (0.78-1.17) | 0.87 (0.71-1.08) |  |
| K ≥ 5.5 | 3.3 (2.3-4.6) | 1.23 (0.85-1.76) | 0.97 (0.67-1.41) |  |
| **Pump failure death** | | | |  |
| K <3.5 | 5.4 (3.7-7.9) | 2.06 (1.39-3.05) | 1.73 (1.16-2.57) |  |
| K ≥ 3.5-<4.0 | 3.1 (2.6-3.7) | 1.32 (1.07-1.63) | 1.07 (0.86-1.32) |  |
| K ≥ 4.0-<4.5 | 2.4 (2.1-2.6) | Reference | Reference |  |
| K ≥ 4.5-<5.0 | 2.3 (2.0-2.5) | 0.91 (0.78-1.06) | 0.85 (0.73-1.00) |  |
| K ≥ 5.0-<5.5 | 2.6 (2.2-3.1) | 0.98 (0.80-1.20) | 0.87 (0.71-1.08) |  |
| K ≥ 5.5 | 3.6 (2.6-5.0) | 1.19 (0.84-1.68) | 0.90 (0.63-1.29) |  |
| **First heart failure hospitalization** | | | |  |
| K <3.5 | 15.3 (12.0-19.4) | 1.63 (1.27-2.09) | 1.26 (0.97-1.62) |  |
| K ≥ 3.5-<4.0 | 10.8 (9.8-12.0) | 1.27 (1.13-1.43) | 1.11 (0.98-1.25) |  |
| K ≥ 4.0-<4.5 | 8.5 (8.0-9.0) | Reference | Reference |  |
| K ≥ 4.5-<5.0 | 8.3 (7.8-8.8) | 0.94 (0.87-1.02) | 0.91 (0.83-0.99) |  |
| K ≥ 5.0-<5.5 | 9.3 (8.4-10.2) | 0.98 (0.88-1.10) | 0.92 (0.81-1.03) |  |
| K ≥ 5.5 | 9.6 (7.8-11.9) | 0.93 (0.75-1.16) | 0.72 (0.58-0.91) |  |
| **First heart failure hospitalization or all-cause death** | | | |  |
| K <3.5 | 27.0 (22.6-32.4) | 1.79 (1.49-2.16) | 1.44 (1.19-1.75) |  |
| K ≥ 3.5-<4.0 | 17.7 (16.3-19.2) | 1.24 (1.13-1.36) | 1.11 (1.01-1.21) |  |
| K ≥ 4.0-<4.5 | 14.4 (13.7-15.0) | Reference | Reference |  |
| K ≥ 4.5-<5.0 | 14.5 (13.9-15.2) | 0.99 (0.93-1.06) | 0.95 (0.89-1.01) |  |
| K ≥ 5.0-<5.5 | 16.3 (15.2-17.6) | 1.05 (0.97-1.15) | 0.96 (0.88-1.05) |  |
| K ≥ 5.5 | 19.2 (16.6-22.4) | 1.17 (1.00-1.37) | 0.87 (0.74-1.03) |  |
| **First heart failure hospitalization or cardiovascular death** | | | |  |
| K <3.5 | 25.0 (20.7-30.2) | 1.82 (1.50-2.21) | 1.45 (1.19-1.76) |  |
| K ≥ 3.5-<4.0 | 16.3 (15.0-17.7) | 1.25 (1.14-1.38) | 1.11 (1.01-1.23) |  |
| K ≥ 4.0-<4.5 | 13.0 (12.4-13.7) | Reference | Reference |  |
| K ≥ 4.5-<5.0 | 13.0 (12.4-13.7) | 0.98 (0.91-1.05) | 0.94 (0.87-1.00) |  |
| K ≥ 5.0-<5.5 | 14.5 (13.4-15.7) | 1.04 (0.95-1.14) | 0.95 (0.86-1.04) |  |
| K ≥ 5.5 | 16.2 (13.8-19.1) | 1.09 (0.92-1.29) | 0.81 (0.68-0.97) |  |

Abbreviations: CI, confidence interval; HFrEF, heart failure with reduced ejection fraction; and LVEF, left ventricular ejection fraction.

P for interaction was assessed using a likelihood ratio test, comparing models with and without the relevant interaction terms for potassium with randomized treatment assignment.

*****Models were stratified according to the trial.

******Models were stratified according to the trial, and adjusted for age, sex, region, race, heart rate, New York Heart Association functional classes III or IV, systolic blood pressure, body mass index, left ventricular ejection fraction, serum creatinine, hemoglobin, NT-proBNP (log-transformed), history of atrial fibrillation, history of myocardial infarction, history of diabetes, diuretics use, mineralocorticoid receptor antagonist use, and angiotensin-converting enzyme inhibitor/angiotensin receptor blocker/angiotensin receptor neprilysin inhibitor use.

In the cases with missing data, the missing indicator method was used for NT-proBNP values.

**Supplemental Table 8**. **Clinical outcomes according to the MRA use in patients with HFrEF**

| **MRA (-) (n = 15,125)** | | | |  |
| --- | --- | --- | --- | --- |
| **HFrEF (LVEF≤40%)** | **Crude incidence rate, 100 patients-year (95% CI)** | **Unadjusted hazard ratios (95% CI)*** | **Adjusted hazard ratios (95% CI)**** | **P for interaction (adjusted hazard ratios) between MRA use and serum potassium levels** |
| **All-cause death** | | | | 0.28 |
| K <3.5 (n=292) | 15.9 (13.0-19.5) | 1.82 (1.47-2.25) | 1.53 (1.23-1.91) |  |
| K ≥ 3.5-<4.0 (n=1,892) | 10.1 (9.2-11.1) | 1.19 (1.07-1.33) | 1.09 (0.98-1.22) |  |
| K ≥ 4.0-<4.5 (n=5,874) | 8.5 (8.0-8.9) | Reference | Reference |  |
| K ≥ 4.5-<5.0 (n=5,222) | 8.7 (8.2-9.2) | 1.02 (0.94-1.11) | 0.96 (0.89-1.05) |  |
| K ≥ 5.0-<5.5 (n=1,542) | 10.0 (9.0-11.1) | 1.14 (1.01-1.28) | 1.00 (0.89-1.13) |  |
| K ≥ 5.5 (n=303) | 14.2 (11.6-17.5) | 1.55 (1.25-1.92) | 1.18 (0.95-1.46) |  |
| **Cardiovascular death** | | | | 0.31 |
| K <3.5 | 13.8 (11.1-17.2) | 1.92 (1.53-2.42) | 1.57 (1.24-1.98) |  |
| K ≥ 3.5-<4.0 | 8.6 (7.8-9.6) | 1.24 (1.10-1.39) | 1.11 (0.98-1.25) |  |
| K ≥ 4.0-<4.5 | 7.0 (6.5-7.4) | Reference | Reference |  |
| K ≥ 4.5-<5.0 | 6.7 (6.3-7.2) | 0.96 (0.87-1.05) | 0.90 (0.82-0.99) |  |
| K ≥ 5.0-<5.5 | 8.0 (7.1-9.0) | 1.12 (0.98-1.28) | 0.99 (0.87-1.14) |  |
| K ≥ 5.5 | 11.3 (9.0-14.2) | 1.53 (1.21-1.95) | 1.13 (0.88-1.44) |  |
| **Non-cardiovascular death** | | | | 0.19 |
| K <3.5 | 1.6 (0.8-3.0) | 1.15 (0.59-2.25) | 1.10 (0.54-2.24) |  |
| K ≥ 3.5-<4.0 | 1.3 (1.0-1.7) | 0.99 (0.74-1.34) | 1.01 (0.75-1.36) |  |
| K ≥ 4.0-<4.5 | 1.3 (1.2-1.5) | Reference | Reference |  |
| K ≥ 4.5-<5.0 | 1.7 (1.5-2.0) | 1.29 (1.06-1.56) | 1.22 (1.00-1.49) |  |
| K ≥ 5.0-<5.5 | 1.7 (1.3-2.2) | 1.19 (0.89-1.60) | 1.05 (0.78-1.41) |  |
| K ≥ 5.5 | 2.3 (1.4-3.8) | 1.58 (0.93-2.68) | 1.32 (0.77-2.25) |  |
| **Sudden death** | | | | 0.80 |
| K <3.5 | 4.7 (3.2-6.9) | 1.70 (1.15-2.51) | 1.44 (0.97-2.13) |  |
| K ≥ 3.5-<4.0 | 3.3 (2.8-3.9) | 1.19 (0.99-1.45) | 1.05 (0.87-1.28) |  |
| K ≥ 4.0-<4.5 | 2.8 (2.5-3.1) | Reference | Reference |  |
| K ≥ 4.5-<5.0 | 2.8 (2.5-3.1) | 1.01 (0.88-1.17) | 0.96 (0.83-1.12) |  |
| K ≥ 5.0-<5.5 | 3.0 (2.5-3.6) | 1.11 (0.90-1.38) | 1.02 (0.82-1.27) |  |
| K ≥ 5.5 | 3.7 (2.5-5.5) | 1.38 (0.91-2.09) | 1.11 (0.72-1.69) |  |
| **Pump failure death** | | | | 0.85 |
| K <3.5 | 5.8 (4.1-8.1) | 2.47 (1.72-3.54) | 1.87 (1.29-2.74) |  |
| K ≥ 3.5-<4.0 | 3.0 (2.5-3.5) | 1.39 (1.13-1.71) | 1.19 (0.96-1.48) |  |
| K ≥ 4.0-<4.5 | 2.1 (1.9-2.3) | Reference | Reference |  |
| K ≥ 4.5-<5.0 | 1.9 (1.7-2.1) | 0.89 (0.75-1.05) | 0.85 (0.71-1.01) |  |
| K ≥ 5.0-<5.5 | 2.2 (1.8-2.7) | 0.97 (0.75-1.24) | 0.86 (0.67-1.12) |  |
| K ≥ 5.5 | 3.6 (2.4-5.4) | 1.41 (0.92-2.15) | 1.01 (0.65-1.56) |  |
| **First heart failure hospitalization** | | | | 0.72 |
| K <3.5 | 14.0 (11.1-17.7) | 1.45 (1.14-1.85) | 1.15 (0.89-1.48) |  |
| K ≥ 3.5-<4.0 | 10.9 (9.9-12.0) | 1.23 (1.10-1.38) | 1.13 (1.00-1.26) |  |
| K ≥ 4.0-<4.5 | 8.6 (8.1-9.1) | Reference | Reference |  |
| K ≥ 4.5-<5.0 | 7.6 (7.2-8.2) | 0.88 (0.80-0.96) | 0.86 (0.78-0.94) |  |
| K ≥ 5.0-<5.5 | 8.9 (7.9-10.0) | 0.98 (0.86-1.11) | 0.94 (0,82-1.07) |  |
| K ≥ 5.5 | 9.0 (6.9-11.8) | 0.93 (0.71-1.22) | 0.77 (0.59-1.02) |  |
| **First heart failure hospitalization or all-cause death** | | | | 0.65 |
| K <3.5 | 25.2 (21.1-30.0) | 1.63 (1.36-1.96) | 1.35 (1.12-1.62) |  |
| K ≥ 3.5-<4.0 | 17.7 (16.5-19.1) | 1.21 (1.11-1.32) | 1.11 (1.02-1.22) |  |
| K ≥ 4.0-<4.5 | 14.4 (13.8-15.1) | Reference | Reference |  |
| K ≥ 4.5-<5.0 | 13.9 (13.3-14.6) | 0.96 (0.90-1.03) | 0.92 (0.86-0.99) |  |
| K ≥ 5.0-<5.5 | 16.2 (14.9-17.6) | 1.07 (0.97-1.18) | 0.99 (0.90-1.10) |  |
| K ≥ 5.5 | 18.8 (15.6-22.6) | 1.19 (0.98-1.44) | 0.93 (0.77-1.13) |  |
| **First heart failure hospitalization or cardiovascular death** | | | | 0.43 |
| K <3.5 | 22.9 (19.1-27.6) | 1.62 (1.34-1.96) | 1.32 (1.08-1.60) |  |
| K ≥ 3.5-<4.0 | 16.5 (15.2-17.8) | 1.23 (1.12-1.35) | 1.12 (1.02-1.23) |  |
| K ≥ 4.0-<4.5 | 13.1 (12.5-13.8) | Reference | Reference |  |
| K ≥ 4.5-<5.0 | 12.2 (11.6-12.9) | 0.93 (0.86-0.99) | 0.89 (0.83-0.95) |  |
| K ≥ 5.0-<5.5 | 14.6 (13.3-16.0) | 1.07 (0.96-1.18) | 0.99 (0.89-1.10) |  |
| K ≥ 5.5 | 16.1 (13.2-19.6) | 1.12 (0.91-1.38) | 0.87 (0.71-1.08) |  |
| **MRA (+) (n=17,221)** | | | |  |
| **HFrEF (LVEF≤40%)** | **Crude incidence rate, 100 patients-year (95% CI)** | **Unadjusted hazard ratios (95% CI)*** | **Adjusted hazard ratios (95% CI)**** |  |
| **All-cause death** | | | |  |
| K <3.5 (n=210) | 20.6 (16.3-26.1) | 1.92 (1.51-2.45) | 1.49 (1.17-1.90) |  |
| K ≥ 3.5-<4.0 (n=1,409) | 12.5 (11.3-13.9) | 1.20 (1.06-1.35) | 1.04 (0.92-1.17) |  |
| K ≥ 4.0-<4.5 (n=5,637) | 10.5 (9.9-11.1) | Reference | Reference |  |
| K ≥ 4.5-<5.0 (n=6,741) | 9.9 (9.4-10.5) | 0.94 (0.87-1.01) | 0.92 (0.85-0.99) |  |
| K ≥ 5.0-<5.5 (n=2,577) | 10.9 (10.1-11.9) | 1.02 (0.92-1.13) | 0.92 (0.83-1.02) |  |
| K ≥ 5.5 (n=647) | 13.5 (11.6-15.8) | 1.21 (1.02-1.42) | 0.93 (0.79-1.11) |  |
| **Cardiovascular death** | | | |  |
| K <3.5 | 18.0 (14.0-23.1) | 2.09 (1.61-2.71) | 1.62 (1.24-2.10) |  |
| K ≥ 3.5-<4.0 | 10.5 (9.4-11.8) | 1.24 (1.09-1.42) | 1.08 (0.94-1.24) |  |
| K ≥ 4.0-<4.5 | 8.5 (8.0-9.0) | Reference | Reference |  |
| K ≥ 4.5-<5.0 | 8.1 (7.6-8.6) | 0.95 (0.87-1.03) | 0.94 (0.86-1.02) |  |
| K ≥ 5.0-<5.5 | 8.8 (8.0-9.6) | 1.02 (0.91-1.14) | 0.93 (0.83-1.05) |  |
| K ≥ 5.5 | 9.9 (8.2-11.8) | 1.11 (0.92-1.35) | 0.87 (0.71-1.06) |  |
| **Non-cardiovascular death** | | | |  |
| K <3.5 | 2.7 (1.4-5.1) | 1.62 (0.83-3.17) | 1.30 (0.64-2.65) |  |
| K ≥ 3.5-<4.0 | 1.5 (1.1-2.0) | 0.94 (0.66-1.32) | 0.82 (0.57-1.18) |  |
| K ≥ 4.0-<4.5 | 1.6 (1.3-1.8) | Reference | Reference |  |
| K ≥ 4.5-<5.0 | 1.5 (1.3-1.7) | 0.93 (0.76-1.14) | 0.85 (0.69-1.05) |  |
| K ≥ 5.0-<5.5 | 1.5 (1.2-1.9) | 0.94 (0.72-1.23) | 0.80 (0.61-1.05) |  |
| K ≥ 5.5 | 2.8 (2.0-3.9) | 1.58 (1.09-2.30) | 1.12 (0.76-1.65) |  |
| **Sudden death** | | | |  |
| K <3.5 | 6.5 (4.3-9.9) | 2.17 (1.41-3.34) | 1.81 (1.17-2.80) |  |
| K ≥ 3.5-<4.0 | 3.5 (2.9-4.3) | 1.18 (0.94-1.48) | 1.07 (0.85-1.35) |  |
| K ≥ 4.0-<4.5 | 3.0 (2.7-3.3) | Reference | Reference |  |
| K ≥ 4.5-<5.0 | 2.9 (2.6-3.2) | 0.96 (0.83-1.11) | 0.99 (0.86-1.15) |  |
| K ≥ 5.0-<5.5 | 2.6 (2.2-3.1) | 0.88 (0.72 (1.08) | 0.86 (0.70-1.05) |  |
| K ≥ 5.5 | 3.3 (2.5-4.6) | 1.15 (0.83-1.60) | 1.00 (0.72-1.40) |  |
| **Pump failure death** | | | |  |
| K <3.5 | 7.4 (5.0-10.9) | 2.29 (1.53-3.44) | 1.62 (1.08-2.44) |  |
| K ≥ 3.5-<4.0 | 4.2 (3.5-5.0) | 1.38 (1.11-1.71) | 1.09 (0.88-1.36) |  |
| K ≥ 4.0-<4.5 | 3.0 (2.7-3.4) | Reference | Reference |  |
| K ≥ 4.5-<5.0 | 2.8 (2.5-3.1) | 0.90 (0.78-1.04) | 0.85 (0.73-0.99) |  |
| K ≥ 5.0-<5.5 | 3.4 (2.9-3.9) | 1.06 (0.88-1.27) | 0.94 (0.78-1.13) |  |
| K ≥ 5.5 | 3.5 (2.6-4.7) | 1.01 (0.73-1.39) | 0.77 (0.56-1.07) |  |
| **First heart failure hospitalization** | | | |  |
| K <3.5 | 22.9 (17.9-29.3) | 1.94 (1.51-2.50) | 1.37 (1.06-1.78) |  |
| K ≥ 3.5-<4.0 | 14.6 (13.1-16.2) | 1.35 (1.19-1.52) | 1.13 (1.00-1.28) |  |
| K ≥ 4.0-<4.5 | 10.6 (10.0-11.3) | Reference | Reference |  |
| K ≥ 4.5-<5.0 | 10.1 (9.6-10.7) | 0.93 (0.86-1.01) | 0.91 (0.84-0.99) |  |
| K ≥ 5.0-<5.5 | 10.8 (9.9-11.8) | 0.97 (0.88-1.08) | 0.91 (0.82-1.02) |  |
| K ≥ 5.5 | 12.6 (10.6-14.9) | 1.02 (0.85-1.22) | 0.84 (0.70-1.01) |  |
| **First heart failure hospitalization or all-cause death** | | | |  |
| K <3.5 | 36.5 (30.1-44.4) | 1.96 (1.60-2.39) | 1.47 (1.20-1.80) |  |
| K ≥ 3.5-<4.0 | 22.5 (20.7-24.5) | 1.27 (1.16-1.41) | 1.10 (0.99-1.21) |  |
| K ≥ 4.0-<4.5 | 17.5 (16.7-18.3) | Reference | Reference |  |
| K ≥ 4.5-<5.0 | 16.8 (16.1-17.6) | 0.95 (0.89-1.01) | 0.93 (0.87-0.99) |  |
| K ≥ 5.0-<5.5 | 18.3 (17.1-19.6) | 1.01 (0.93-1.10) | 0.94 (0.86-1.02) |  |
| K ≥ 5.5 | 23.2 (20.4-26.2) | 1.20 (0.83-1.09) | 0.95 (0.83-1.10) |  |
| **First heart failure hospitalization or cardiovascular death** | | | |  |
| K <3.5 | 33.7 (27.5-41.2) | 1.98 (1.61-2.44) | 1.48 (1.20-1.83) |  |
| K ≥ 3.5-<4.0 | 20.9 (19.1-22.8) | 1.30 (1.17-1.44) | 1.11 (1.00-1.23) |  |
| K ≥ 4.0-<4.5 | 15.9 (15.1-16.7) | Reference | Reference |  |
| K ≥ 4.5-<5.0 | 15.4 (14.7-16.1) | 0.95 (0.89-1.02) | 0.93 (0.87-1.00) |  |
| K ≥ 5.0-<5.5 | 16.4 (15.2-17.6) | 1.00 (0.92-1.09) | 0.93 (0.85-1.01) |  |
| K ≥ 5.5 | 19.9 (17.3-22.7) | 1.13 (0.98-1.31) | 0.91 (0.78-1.05) |  |

Abbreviations: CI, confidence interval; HFrEF, heart failure with reduced ejection fraction; LVEF, left ventricular ejection fraction; and MRA, mineralocorticoid receptor antagonist.

P for interaction was assessed using a likelihood ratio test, comparing models with and without the relevant interaction terms for potassium with MRA use.

*****Models were stratified according to the trial and with the treatment assignment as covariate.

******Models were stratified according to the trial and with the treatment assignment as covariate, and adjusted for age, sex, region, race, heart rate, New York Heart Association functional classes III or IV, systolic blood pressure, body mass index, left ventricular ejection fraction, serum creatinine, hemoglobin, NT-proBNP (log-transformed), history of atrial fibrillation, history of myocardial infarction, history of diabetes, diuretics use, and angiotensin-converting enzyme inhibitor/angiotensin receptor blocker/angiotensin receptor neprilysin inhibitor use.

In the cases with missing data, the missing indicator method was used for NT-proBNP values.

**Supplemental Table 9**. **Potassium level associated with the lowest risk of clinical outcomes, based on binary recursive partitioning analysis, hazard ratio spline, and incidence rate spline.**

| HFrEF |  |  |  |
| --- | --- | --- | --- |
| **Outcome** | **Binary recursive partitioning** | **Nadir of HR** | **Nadir of incidence rate** |
| All-cause death | 3.7-5.0 | 4.7 | 4.4 |
| CV death | 3.7-5.0 | 4.7 | 4.4 |
| Sudden death | ≥3.4 | 4.7 | 4.4 |
| Pump failure death | 3.7-5.1 | 4.9 | 4.5 |
| Non-CV death | ≤5.5 | 4.1 | 4.2 |
| First HF hospitalization | 4.2-5.1 | - | 4.5 |
| First HF hospitalization + All-cause death | 4.1-5.0 | 4.7 | 4.4 |
| First HF hospitalization + CV death | 4.1-5.0 | 4.8 | 4.4 |
|  |  |  |  |
| HFpEF |  |  |  |
| **Outcome** | **Binary recursive partitioning** | **Nadir of HR** | **Nadir of incidence rate** |
| All-cause death | 3.8-5.3 | 4.4 | 4.3 |
| CV death | ≤5.1 | 4.4 | 4.3 |
| Sudden death | ≤5.6 | - | - |
| Pump failure death | 3.9-5.1 | 4.4 | 4.5 |
| Non-CV death | - | 4.5 | 4.4 |
| First HF hospitalization | 4.2-5.1 | 4.6 | 4.6 |
| First HF hospitalization + All-cause death | 3.9-5.1 | 4.5 | 4.5 |
| First HF hospitalization + CV death | 3.9-5.1 | 4.5 | 4.5 |

The unit of potassium is mmol/L.

For the HR calculation, models were stratified according to the trial and with the treatment assignment as covariate, and adjusted for age, sex, region, race, heart rate, New York Heart Association functional classes III or IV, systolic blood pressure, body mass index, left ventricular ejection fraction, serum creatinine, hemoglobin, NT-proBNP (log-transformed), history of atrial fibrillation, history of myocardial infarction, history of diabetes, diuretics use, mineralocorticoid receptor antagonist use, and angiotensin-converting enzyme inhibitor/angiotensin receptor blocker/angiotensin receptor neprilysin inhibitor use. In the cases with missing data, the missing indicator method was used for NT-proBNP values.

Abbreviations: CV, cardiovascular; HF, heart failure; HFpEF, heart failure with preserved ejection fraction; HFrEF, heart failure with reduced ejection fraction; and HR, hazard ratio.

**Supplemental Table 10**. **Clinical outcomes according to the treatment assignment (control vs intervention) in patients with HFpEF**

| **Controls (n = 6,844)** | | | |  |
| --- | --- | --- | --- | --- |
| **HFpEF (LVEF≥50%)** | **Crude incidence rate, 100 patients-year (95% CI)** | **Unadjusted hazard ratios (95% CI)*** | **Adjusted hazard ratios (95% CI)**** | **P for interaction (adjusted hazard ratios) between treatment assignment and serum potassium levels** |
| **All-cause death** | | | | 0.78 |
| K <3.5 (n=145) | 7.5 (5.2-10.7) | 1.30 (0.89-1.88) | 1.19 (0.82-1.74) |  |
| K ≥ 3.5-<4.0 (n=978) | 6.0 (5.2-7.0) | 1.14 (0.95-1.35) | 1.08 (0.90-1.29) |  |
| K ≥ 4.0-<4.5 (n=2,629) | 5.2 (4.7-5.7) | Reference | Reference |  |
| K ≥ 4.5-<5.0 (n=2,315) | 5.5 (5.0-6.1) | 1.09 (0.95-1.25) | 1.01 (0.88-1.16) |  |
| K ≥ 5.0-<5.5 (n=681) | 5.4 (4.5-6.5) | 1.13 (0.92-1.39) | 1.01 (0.81-1.25) |  |
| K ≥ 5.5 (n=96) | 9.3 (6.3-13.7) | 1.97 (1.32-2.93) | 1.50 (1.00-2.25) |  |
| **Cardiovascular death** | | | | 0.99 |
| K <3.5 | 4.0 (2.4-6.5) | 1.26 (0.76-2.10) | 1.17 (0.69-1.98) |  |
| K ≥ 3.5-<4.0 | 3.5 (2.9-4.2) | 1.14 (0.91-1.44) | 1.14 (0.90-1.44) |  |
| K ≥ 4.0-<4.5 | 3.0 (2.6-3.4) | Reference | Reference |  |
| K ≥ 4.5-<5.0 | 3.1 (2.7-3.5) | 1.08 (0.90-1.30) | 1.04 (0.87-1.26) |  |
| K ≥ 5.0-<5.5 | 3.7 (2.9-4.6) | 1.32 (1.02-1.71) | 1.19 (0.91-1.56) |  |
| K ≥ 5.5 | 5.0 (3.0-8.5) | 1.82 (1.06-3.13) | 1.38 (0.79-2.39) |  |
| **Non-cardiovascular death** | | | | 0.12 |
| K <3.5 | 3.5 (2.1-5.9) | 1.73 (1.00-2.99) | 1.54 (0.88-2.67) |  |
| K ≥ 3.5-<4.0 | 2.2 (1.7-2.8) | 1.16 (0.87-1.56) | 1.05 (0.77-1.42) |  |
| K ≥ 4.0-<4.5 | 1.9 (1.6-2.2) | Reference | Reference |  |
| K ≥ 4.5-<5.0 | 2.0 (1.7-2.3) | 1.07 (0.85-1.35) | 0.94 (0.74-1.19) |  |
| K ≥ 5.0-<5.5 | 1.6 (1.1-2.2) | 0.90 (0.62-1.31) | 0.74 (0.50-1.10) |  |
| K ≥ 5.5 | 3.9 (2.2-7.1) | 2.32 (1.26-4.28) | 1.74 (0.93-3.26) |  |
| **Sudden death** | | | | 0.49 |
| K <3.5 | 0.7 (0.2-2.3) | 0.70 (0.22-2.22) | 0.73 (0.23-2.31) |  |
| K ≥ 3.5-<4.0 | 1.2 (0.8-1.6) | 1.09 (0.73-1.63) | 1.06 (0.70-1.60) |  |
| K ≥ 4.0-<4.5 | 1.0 (0.8-1.3) | Reference | Reference |  |
| K ≥ 4.5-<5.0 | 1.2 (1.0-1.5) | 1.21 (0.90-1.63) | 1.15 (0.85-1.56) |  |
| K ≥ 5.0-<5.5 | 1.0 (0.6-1.5) | 0.95 (0.59-1.54) | 0.79 (0.48-1.31) |  |
| K ≥ 5.5 | 2.5 (1.2-5.3) | 2.50 (1.16-5.42) | 1.75 (0.79-3.87) |  |
| **Pump failure death** | | | | 0.75 |
| K <3.5 | 1.7 (0.8-3.7) | 1.81 (0.83-3.95) | 1.69 (0.77-3.71) |  |
| K ≥ 3.5-<4.0 | 1.0 (0.7-1.4) | 1.11 (0.71-1.72) | 1.06 (0.68-1.66) |  |
| K ≥ 4.0-<4.5 | 0.8 (0.7-1.1) | Reference | Reference |  |
| K ≥ 4.5-<5.0 | 0.7 (0.5-0.9) | 0.87 (0.60-1.25) | 0.86 (0.59-1.24) |  |
| K ≥ 5.0-<5.5 | 1.0 (0.6-1.5) | 1.32 (0.80-2.17) | 1.16 (0.69-1.95) |  |
| K ≥ 5.5 | 1.8 (0.7-4.3) | 2.43 (0.98-6.03) | 1.45 (0.56-3.72) |  |
| **First heart failure hospitalization** | | | | 0.60 |
| K <3.5 | 8.9 (6.3-12.7) | 1.29 (0.90-1.84) | 1.13 (0.78-1.62) |  |
| K ≥ 3.5-<4.0 | 7.7 (6.7-8.8) | 1.23 (1.04-1.45) | 1.09 (0.92-1.29) |  |
| K ≥ 4.0-<4.5 | 6.1 (5.6-6.7) | Reference | Reference |  |
| K ≥ 4.5-<5.0 | 5.7 (5.2-6.3) | 0.96 (0.83-1.10) | 0.92 (0.80-1.06) |  |
| K ≥ 5.0-<5.5 | 6.5 (5.5-7.7) | 1.18 (0.97-1.44) | 0.99 (0.80-1.22) |  |
| K ≥ 5.5 | 6.7 (4.2-10.8) | 1.21 (0.74-1.97) | 0.76 (0.46-1.26) |  |
| **First heart failure hospitalization or all-cause death** | | | | 0.54 |
| K <3.5 | 14.5 (11.1-19.1) | 1.33 (1.01-1.77) | 1.18 (0.88-1.57) |  |
| K ≥ 3.5-<4.0 | 11.5 (10.3-12.9) | 1.15 (1.01-1.32) | 1.05 (0.91-1.20) |  |
| K ≥ 4.0-<4.5 | 9.8 (9.1-10.5) | Reference | Reference |  |
| K ≥ 4.5-<5.0 | 9.8 (9.1-10.6) | 1.02 (0.92-1.14) | 0.97 (0.87-1.09) |  |
| K ≥ 5.0-<5.5 | 10.0 (8.7-11.6) | 1.12 (0.95-1.31) | 0.97 (0.82-1.14) |  |
| K ≥ 5.5 | 13.5 (9.6-18.8) | 1.49 (1.06-2.11) | 1.02 (0.72-1.46) |  |
| **First heart failure hospitalization or cardiovascular death** | | | | 0.51 |
| K <3.5 | 11.7 (8.7-15.9) | 1.33 (0.97-1.82) | 1.17 (0.85-1.61) |  |
| K ≥ 3.5-<4.0 | 9.6 (8.5-10.9) | 1.17 (1.01-1.36) | 1.07 (0.92-1.25) |  |
| K ≥ 4.0-<4.5 | 8.0 (7.4-8.7) | Reference | Reference |  |
| K ≥ 4.5-<5.0 | 7.8 (7.2-8.5) | 1.00 (0.89-1.12) | 0.97 (0.86-1.09) |  |
| K ≥ 5.0-<5.5 | 8.8 (7.5-10.2) | 1.19 (1.00-1.41) | 1.02 (0.86-1.23) |  |
| K ≥ 5.5 | 9.9 (6.7-14.6) | 1.33 (0.89-1.99) | 0.89 (0.59-1.35) |  |
| **Interventions (n = 6,879)** | | | |  |
| **HFpEF (LVEF≥50%)** | **Crude incidence rate, 100 patients-year (95% CI)** | **Unadjusted hazard ratios (95% CI)*** | **Adjusted hazard ratios (95% CI) **** |  |
| **All-cause death** | | | |  |
| K <3.5 (n=145) | 7.3 (5.1-10.4) | 1.31 (0.90-1.91) | 1.07 (0.73-1.56) |  |
| K ≥ 3.5-<4.0 (n=990) | 5.6 (4.9-6.6) | 1.09 (0.91-1.30) | 1.05 (0.87-1.26) |  |
| K ≥ 4.0-<4.5 (n=2,727) | 5.0 (4.6-5.5) | Reference | Reference |  |
| K ≥ 4.5-<5.0 (n=2,287) | 5.1 (4.6-5.7) | 1.04 (0.90-1.20) | 1.02 (0.89-1.18) |  |
| K ≥ 5.0-<5.5 (n=645) | 6.1 (5.1-7.3) | 1.31 (1.07-1.60) | 1.10 (0.89-1.36) |  |
| K ≥ 5.5 (n=85) | 8.7 (5.7-13.1) | 1.87 (1.21-2.87) | 1.08 (0.69-1.70) |  |
| **Cardiovascular death** | | | |  |
| K <3.5 | 3.9 (2.4-6.3) | 1.38 (0.83-2.30) | 1.10 (0.65-1.84) |  |
| K ≥ 3.5-<4.0 | 3.6 (3.0-4.4) | 1.29 (1.03-1.62) | 1.26 (0.99-1.59) |  |
| K ≥ 4.0-<4.5 | 2.7 (2.4-3.1) | Reference | Reference |  |
| K ≥ 4.5-<5.0 | 2.9 (2.5-3.3) | 1.06 (0..88-1.28) | 1.03 (0.85-1.25) |  |
| K ≥ 5.0-<5.5 | 3.8 (3.0-4.7) | 1.43 (1.10-1.85) | 1.18 (0.90-1.55) |  |
| K ≥ 5.5 | 5.9 (3.6-9.8) | 2.23 (1.32-3.76) | 1.13 (0.64-1.97) |  |
| **Non-cardiovascular death** | | | |  |
| K <3.5 | 2.7 (1.5-4.8) | 1.23 (0.67-2.28) | 1.05 (0.57-1.97) |  |
| K ≥ 3.5-<4.0 | 1.7 (1.3-2.2) | 0.87 (0.64-1.19) | 0.83 (0.59-1.16) |  |
| K ≥ 4.0-<4.5 | 1.9 (1.6-2.2) | Reference | Reference |  |
| K ≥ 4.5-<5.0 | 1.9 (1.6-2.3) | 1.08 (0.86-1.35) | 1.07 (0.85-1.36) |  |
| K ≥ 5.0-<5.5 | 2.0 (1.5-2.8) | 1.21 (0.85-1.71) | 1.04 (0.73-1.50) |  |
| K ≥ 5.5 | 1.2 (0.4-3.7) | 0.71 (0.23-2.22) | 0.52 (0.17-1.66) |  |
| **Sudden death** | | | |  |
| K <3.5 | 0.7 (0.2-2.3) | 0.81 (0.25-2.57) | 0.70 (0.22-2.25) |  |
| K ≥ 3.5-<4.0 | 0.8 (0.5-1.1) | 0.83 (0.52-1.32) | 0.85 (0.53-1.36) |  |
| K ≥ 4.0-<4.5 | 0.9 (0.7-1.1) | Reference | Reference |  |
| K ≥ 4.5-<5.0 | 1.1 (0.8-1.3) | 1.19 (0.87-1.64) | 1.18 (0.85-1.63) |  |
| K ≥ 5.0-<5.5 | 1.5 (1.0-2.1) | 1.70 (1.11-2.61) | 1.43 (0.92-2.22) |  |
| K ≥ 5.5 | 2.8 (1.3-5.8) | 3.08 (1.42-6.70) | 2.11 (0.95-4.72) |  |
| **Pump failure death** | | | |  |
| K <3.5 | 1.5 (0.7-3.2) | 1.81 (0.78-4.21) | 1.38 (0.58-3.26) |  |
| K ≥ 3.5-<4.0 | 1.3 (1.0-1.8) | 1.71 (1.16-2.54) | 1.56 (1.02-2.36) |  |
| K ≥ 4.0-<4.5 | 0.8 (0.6-1.0) | Reference | Reference |  |
| K ≥ 4.5-<5.0 | 0.8 (0.6-1.1) | 1.09 (0.76-1.55) | 1.11 (0.77-1.62) |  |
| K ≥ 5.0-<5.5 | 0.8 (0.5-1.4) | 1.15 (0.67-1.96) | 1.04 (0.60-1.81) |  |
| K ≥ 5.5 | 2.4 (1.1-5.3) | 3.26 (1.41-7.56) | 1.44 (0.59-3.53) |  |
| **First heart failure hospitalization** | | | |  |
| K <3.5 | 7.7 (5.3-11.1) | 1.22 (0.83-1.79) | 1.01 (0.69-1.49) |  |
| K ≥ 3.5-<4.0 | 7.1 (6.2-8.2) | 1.25 (1.06-1.48) | 1.20 (1.01-1.43) |  |
| K ≥ 4.0-<4.5 | 5.6 (5.1-6.1) | Reference | Reference |  |
| K ≥ 4.5-<5.0 | 5.4 (4.8-6.0) | 0.98 (0.85-1.13) | 1.00 (0.86-1.15) |  |
| K ≥ 5.0-<5.5 | 5.8 (4.8-7.0) | 1.10 (0.89-1.36) | 1.00 (0.80-1.25) |  |
| K ≥ 5.5 | 9.3 (6.0-14.4) | 1.72 (1.10-2.70) | 1.32 (0.82-2.10) |  |
| **First heart failure hospitalization or all-cause death** | | | |  |
| K <3.5 | 12.6 (9.4-16.8) | 1.24 (0.92-1.67) | 1.04 (0.77-1.41) |  |
| K ≥ 3.5-<4.0 | 10.7 (9.5-12.0) | 1.14 (1.00-1.31) | 1.11 (0.97-1.28) |  |
| K ≥ 4.0-<4.5 | 9.1 (8.4-9.8) | Reference | Reference |  |
| K ≥ 4.5-<5.0 | 8.9 (8.2-9.7) | 1.00 (0.89-1.11) | 0.99 (0.88-1.10) |  |
| K ≥ 5.0-<5.5 | 10.6 (9.2-12.2) | 1.23 (1.05-1.44) | 1.08 (0.91-1.27) |  |
| K ≥ 5.5 | 15.7 (11.2-22.0) | 1.81 (1.28-2.56) | 1.32 (0.92-1.89) |  |
| **First heart failure hospitalization or cardiovascular death** | | | |  |
| K <3.5 | 10.1 (7.3-14.0) | 1.26 (0.90-1.75) | 1.04 (0.74-1.46) |  |
| K ≥ 3.5-<4.0 | 9.3 (8.2-10.5) | 1.24 (1.07-1.44) | 1.20 (1.03-1.40) |  |
| K ≥ 4.0-<4.5 | 7.3 (6.7-7.9) | Reference | Reference |  |
| K ≥ 4.5-<5.0 | 7.2 (6.6-7.9) | 1.00 (0.89-1.13) | 1.00 (0.88-1.13) |  |
| K ≥ 5.0-<5.5 | 8.7 (7.5-10.2) | 1.25 (1.05-1.50) | 1.11 (0.92-1.33) |  |
| K ≥ 5.5 | 13.0 (9.0-18.8) | 1.84 (1.26-2.69) | 1.32 (0.89-1.97) |  |

Abbreviations: CI, confidence interval; HFpEF, heart failure with preserved ejection fraction; and LVEF, left ventricular ejection fraction.

P for interaction was assessed using a likelihood ratio test, comparing models with and without the relevant interaction terms for potassium with randomized treatment assignment.

*****Models were stratified according to the trial.

******Models were stratified according to the trial, and adjusted for age, sex, region, race, heart rate, New York Heart Association functional classes III or IV, systolic blood pressure, body mass index, left ventricular ejection fraction, serum creatinine, hemoglobin, NT-proBNP (log-transformed), history of atrial fibrillation, history of myocardial infarction, history of diabetes, diuretics use, mineralocorticoid receptor antagonist use, and angiotensin-converting enzyme inhibitor/angiotensin receptor blocker/angiotensin receptor neprilysin inhibitor use.

In the cases with missing data, the missing indicator method was used for NT-proBNP values.

**Supplemental Table 11**. **Clinical outcomes according to the MRA use in patients with HFpEF**

| **MRA (-) (n = 12,179)** | | | |  |
| --- | --- | --- | --- | --- |
| **HFpEF (LVEF≥50%)** | **Crude incidence rate, 100 patients-year (95% CI)** | **Unadjusted hazard ratios (95% CI)*** | **Adjusted hazard ratios (95% CI)**** | **P for interaction (adjusted hazard ratios) between MRA use and serum potassium levels** |
| **All-cause death** | | | | 0.73 |
| K <3.5 (n=273) | 7.7 (5.9-9.9) | 1.34 (1.02-1.75) | 1.20 (0.91-1.57) |  |
| K ≥ 3.5-<4.0 (n=1,841) | 5.8 (5.2-6.5) | 1.12 (0.98-1.27) | 1.06 (0.93-1.22) |  |
| K ≥ 4.0-<4.5 (n=4,869) | 5.1 (4.7-5.4) | Reference | Reference |  |
| K ≥ 4.5-<5.0 (n=4,004) | 5.2 (4.9-5.7) | 1.06 (0.95-1.17) | 1.03 (0.92-1.14) |  |
| K ≥ 5.0-<5.5 (n=1,052) | 5.4 (4.7-6.3) | 1.15 (0.98-1.36) | 1.04 (0.88-1.23) |  |
| K ≥ 5.5 (n=140) | 8.1 (5.7-11.3) | 1.72 (1.21-2.43) | 1.28 (0.90-1.84) |  |
| **Cardiovascular death** | | | | 0.10 |
| K <3.5 | 4.2 (3.0-6.0) | 1.41 (0.98-2.02) | 1.25 (0.87-1.81) |  |
| K ≥ 3.5-<4.0 | 3.5 (3.0-4.0) | 1.20 (1.01-1.43) | 1.16 (0.98-1.39) |  |
| K ≥ 4.0-<4.5 | 2.8 (2.5-3.1) | Reference | Reference |  |
| K ≥ 4.5-<5.0 | 2.9 (2.6-3.2) | 1.07 (0.93-1.23) | 1.05 (0.91-1.22) |  |
| K ≥ 5.0-<5.5 | 3.5 (2.9-4.1) | 1.30 (1.06-1.61) | 1.17 (0.94-1.45) |  |
| K ≥ 5.5 | 4.4 (2.8-7.0) | 1.67 (1.04-2.67) | 1.14 (0.70-1.87) |  |
| **Non-cardiovascular death** | | | | 0.70 |
| K <3.5 | 3.2 (2.1-4.7) | 1.50 (0.98-2.27) | 1.37 (0.90-2.08) |  |
| K ≥ 3.5-<4.0 | 2.0 (1.7-2.3) | 1.04 (0.84-1.30) | 0.97 (0.77-1.22) |  |
| K ≥ 4.0-<4.5 | 1.9 (1.7-2.1) | Reference | Reference |  |
| K ≥ 4.5-<5.0 | 1.9 (1.7-2.2) | 1.04 (0.88-1.24) | 0.99 (0.83-1.18) |  |
| K ≥ 5.0-<5.5 | 1.7 (1.3-2.2) | 0.98 (0.74) | 0.86 (0.64-1.16) |  |
| K ≥ 5.5 | 2.4 (1.3-4.5) | 1.39 (0.74-2.62) | 1.18 (0.62-2.24) |  |
| **Sudden death** | | | | 0.26 |
| K <3.5 | 0.8 (0.4-1.8) | 0.77 (0.34-1.75) | 0.74 (0.33-1.68) |  |
| K ≥ 3.5-<4.0 | 0.9 (0.7-1.2) | 0.88 (0.63-1.21) | 0.86 (0.62-1.19) |  |
| K ≥ 4.0-<4.5 | 1.0 (0.8-1.2) | Reference | Reference |  |
| K ≥ 4.5-<5.0 | 1.1 (1.0-1.3) | 1.15 (0.91-1.45) | 1.12 (0.89-1.42) |  |
| K ≥ 5.0-<5.5 | 1.1 (0.8-1.6) | 1.18 (0.82-1.68) | 0.97 (0.67-1.42) |  |
| K ≥ 5.5 | 2.4 (1.3-4.5) | 2.53 (1.33-4.80) | 1.87 (0.97-3.59) |  |
| **Pump failure death** | | | | 0.67 |
| K <3.5 | 1.7 (1.0-3.0) | 1.95 (1.09-3.47) | 1.62 (0.90-2.91) |  |
| K ≥ 3.5-<4.0 | 1.1 (.9-1.5) | 1.44 (1.06-1.96) | 1.31 (0.95-1.80) |  |
| K ≥ 4.0-<4.5 | 0.8 (0.6-0.9) | Reference | Reference |  |
| K ≥ 4.5-<5.0 | 0.7 (0.6-0.9) | 0.98 (0.74-1.29) | 0.99 (0.74-1.32) |  |
| K ≥ 5.0-<5.5 | 0.8 (0.6-1.2) | 1.16 (0.76-1.77) | 1.16 (0.75-1.78) |  |
| K ≥ 5.5 | 1.5 (0.7-3.3) | 2.08 (0.91-4.72) | 1.10 (0.47-2.62) |  |
| **First heart failure hospitalization** | | | | 0.47 |
| K <3.5 | 8.2 (6.3-10.6) | 1.26 (0.96-1.66) | 1.12 (0.85-1.48) |  |
| K ≥ 3.5-<4.0 | 7.3 (6.6-8.1) | 1.28 (1.13-1.44) | 1.17 (1.03-1.33) |  |
| K ≥ 4.0-<4.5 | 5.6 (5.2-6.0) | Reference | Reference |  |
| K ≥ 4.5-<5.0 | 5.2 (4.8-5.7) | 0.96 (0.86-1.07) | 0.97 (0.87-1.08) |  |
| K ≥ 5.0-<5.5 | 5.8 (5.0-6.7) | 1.15 (0.97-1.35) | 1.06 (0.89-1.26) |  |
| K ≥ 5.5 | 7.5 (5.1-10.9) | 1.42 (0.97-2.09) | 1.09 (0.73-1.64) |  |
| **First heart failure hospitalization or all-cause death** | | | | 0.61 |
| K <3.5 | 13.7 (11.1-16.8) | 1.31 (1.06-1.62) | 1.18 (0.95-1.46) |  |
| K ≥ 3.5-<4.0 | 11.0 (10.1-11.9) | 1.16 (1.05-1.29) | 1.10 (0.99-1.22) |  |
| K ≥ 4.0-<4.5 | 9.2 (8.7-9.7) | Reference | Reference |  |
| K ≥ 4.5-<5.0 | 9.1 (8.5-9.6) | 1.01 (0.93-1.09) | 0.99 (0.91-1.08) |  |
| K ≥ 5.0-<5.5 | 9.7 (8.7-10.9) | 1.16 (1.02-1.31) | 1.05 (0.92-1.20) |  |
| K ≥ 5.5 | 13.6 (10.3-18.0) | 1.57 (1.18-2.09) | 1.23 (0.91-1.65) |  |
| **First heart failure hospitalization or cardiovascular death** | | | | 0.62 |
| K <3.5 | 11.0 (8.7-13.8) | 1.32 (1.04-1.67) | 1.18 (0.93-1.50) |  |
| K ≥ 3.5-<4.0 | 9.3 (8.5-10.2) | 1.22 (1.09-1.36) | 1.15 (1.03-1.29) |  |
| K ≥ 4.0-<4.5 | 7.4 (6.9-7.9) | Reference | Reference |  |
| K ≥ 4.5-<5.0 | 7.2 (6.8-7.7) | 1.00 (0.91-1.10) | 1.00 (0.91-1.10) |  |
| K ≥ 5.0-<5.5 | 8.2 (7.2-9.3) | 1.21 (1.05-1.39) | 1.10 (0.96-1.28) |  |
| K ≥ 5.5 | 10.6 (7.7-14.5) | 1.50 (1.09-2.08) | 1.14 (0.81-1.61) |  |
| **MRA (+) (n=1,542)** | | | |  |
| **HFpEF (LVEF≥50%)** | **Crude incidence rate, 100 patients-year (95% CI)** | **Unadjusted hazard ratios (95% CI)*** | **Adjusted hazard ratios (95% CI)**** |  |
| **All-cause death** | | | |  |
| K <3.5 (n=17) | 3.6 (0.9-14.4) | 0.71 (0.18-2.90) | 0.42 (0.10-1.79) |  |
| K ≥ 3.5-<4.0 (n=127) | 6.1 (4.1-9.1) | 1.10 (0.71-1.72) | 1.20 (0.76-1.90) |  |
| K ≥ 4.0-<4.5 (n=486) | 5.5 (4.4-6.7) | Reference | Reference |  |
| K ≥ 4.5-<5.0 (n=597) | 5.7 (4.7-6.9) | 1.09 (0.82-1.44) | 1.03 (0.77-1.37) |  |
| K ≥ 5.0-<5.5 (n=274) | 7.0 (5.5-9.0) | 1.34 (0.97-1.86) | 1.10 (0.78-1.55) |  |
| K ≥ 5.5 (n=41) | 12.0 (7.3-20.0) | 2.23 (1.29-3.86) | 1.21 (0.67-2.17) |  |
| **Cardiovascular death** | | | |  |
| K <3.5 | 0 (0) | 0 (0) | 0 (0) |  |
| K ≥ 3.5-<4.0 | 4.9 (3.2-7.6) | 1.42 (0.85-2.37) | 1.56 (0.92-2.65) |  |
| K ≥ 4.0-<4.5 | 3.3 (2.6-4.4) | Reference | Reference |  |
| K ≥ 4.5-<5.0 | 3.3 (2.6-4.3) | 1.06 (0.74-1.52) | 1.01 (0.70-1.47) |  |
| K ≥ 5.0-<5.5 | 4.7 (3.4-6.4) | 1.47 (0.98-2.21) | 1.16 (0.75-1.79) |  |
| K ≥ 5.5 | 8.8 (4.9-15.9) | 2.61 (0.62-1.10) | 1.40 (0.70-2.82) |  |
| **Non-cardiovascular death** | | | |  |
| K <3.5 | 1.8 (0.3-12.8) | 1.13 (0.15-8.38) | 0.49 (0.06-4.12) |  |
| K ≥ 3.5-<4.0 | 1.0 (0.4-2.6) | 0.57 (0.20-1.63) | 0.63 (0.22-1.83) |  |
| K ≥ 4.0-<4.5 | 1.7 (1.1-2.4) | Reference | Reference |  |
| K ≥ 4.5-<5.0 | 2.1 (1.6-2.9) | 1.34 (0.82-2.18) | 1.18 (0.71-1.94) |  |
| K ≥ 5.0-<5.5 | 2.1 (1.3-3.3) | 1.32 (0.72-2.39) | 1.08 (0.58-2.02) |  |
| K ≥ 5.5 | 3.2 (1.2-8.6) | 2.00 (0.70-5.73) | 1.01 (0.33-3.11) |  |
| **Sudden death** | | | |  |
| K <3.5 | 0 (0) | 0 (0) | 0 (0) |  |
| K ≥ 3.5-<4.0 | 2.0 (1.0-3.9) | 2.35 (0.97-5.67) | 2.44 (0.99-6.01) |  |
| K ≥ 4.0-<4.5 | 0.8 (0.5-1.4) | Reference | Reference |  |
| K ≥ 4.5-<5.0 | 1.3 (0.9-1.9) | 1.77 (0.90-3.46) | 1.74 (0.88-3.45) |  |
| K ≥ 5.0-<5.5 | 1.5 (0.9-2.6) | 2.01 (0.93-4.35) | 1.78 (0.79-3.99) |  |
| K ≥ 5.5 | 3.2 (1.2-8.6) | 3.95 (1.29-12.16) | 2.32 (0.67-8.01) |  |
| **Pump failure death** | | | |  |
| K <3.5 | 0 (0) | 0 (0) | 0 (0) |  |
| K ≥ 3.5-<4.0 | 1.2 (0.5-2.9) | 1.08 (0.40-2.91) | 1.11 (0.39-3.14) |  |
| K ≥ 4.0-<4.5 | 1.1 (0.7-1.8) | Reference | Reference |  |
| K ≥ 4.5-<5.0 | 0.9 (0.6-1.5) | 1.08 (0.40-2.91) | 0.85 (0.43-1.68) |  |
| K ≥ 5.0-<5.5 | 1.3 (0.7-2.3) | 1.19 (0.56-2.53) | 0.94 (0.42-2.07) |  |
| K ≥ 5.5 | 4.0 (1.7-9.6) | 3.64 (1.35-9.85) | 2.78 (0.96-8.05) |  |
| **First heart failure hospitalization** | | | |  |
| K <3.5 | 10.1 (4.2-24.2) | 1.20 (0.49-2.95) | 0.88 (0.35-2.24) |  |
| K ≥ 3.5-<4.0 | 8.2 (5.7-11.8) | 1.02 (0.68-1.53) | 0.88 (0.57-1.33) |  |
| K ≥ 4.0-<4.5 | 8.1 (6.8-9.8) | Reference | Reference |  |
| K ≥ 4.5-<5.0 | 7.6 (6.4-9.0) | 0.92 (0.72-1.19) | 0.91 (0.70-1.18) |  |
| K ≥ 5.0-<5.5 | 7.7 (6.0-10.0) | 0.94 (0.69-1.29) | 0.87 (0.62-1.20) |  |
| K ≥ 5.5 | 9.2 (5.0-17.1) | 1.13 (0.59-2.16) | 0.84 (0.43-1.63) |  |
| **First heart failure hospitalization or all-cause death** | | | |  |
| K <3.5 | 12.1 (5.4-26.9) | 1.04 (0.46-2.35) | 0.71 (0.31-1.66) |  |
| K ≥ 3.5-<4.0 | 12.8 (9.5-17.1) | 1.07 (0.77-1.49) | 0.96 (0.69-1.35) |  |
| K ≥ 4.0-<4.5 | 11.9 (10.2-13.8) | Reference | Reference |  |
| K ≥ 4.5-<5.0 | 114 (9.9-13.1) | 0.97 (0.79-1.19) | 0.94 (0.76-1.16) |  |
| K ≥ 5.0-<5.5 | 12.6 (10.3-15.4) | 1.07 (0.83-1.37) | 0.94 (0.72-1.23) |  |
| K ≥ 5.5 | 17.5 (11.2-27.5) | 1.48 (0.92-2.37) | 1.02 (0.62-1.66) |  |
| **First heart failure hospitalization or cardiovascular death** | | | |  |
| K <3.5 | 10.1 (4.2-24.2) | 1.03 (0.42-2.51) | 0.68 (0.27-1.72) |  |
| K ≥ 3.5-<4.0 | 11.6 (8.6-15.8) | 1.15 (0.81-1.62) | 1.01 (0.71-1.45) |  |
| K ≥ 4.0-<4.5 | 10.1 (8.6-11.9) | Reference | Reference |  |
| K ≥ 4.5-<5.0 | 9.4 (8.0-11.0) | 0.94 (0.75-1.18) | 0.92 (0.73-1.16) |  |
| K ≥ 5.0-<5.5 | 10.9 (8.8-13.5) | 1.08 (0.83-1.42) | 0.95 (0.72-1.27) |  |
| K ≥ 5.5 | 13.8 (8.3-22.9) | 1.36 (0.80-2.32) | 0.95 (0.54-1.65) |  |

Abbreviations: CI, confidence interval; HFpEF, heart failure with preserved ejection fraction; LVEF, left ventricular ejection fraction; and MRA, mineralocorticoid receptor antagonist.

P for interaction was assessed using a likelihood ratio test, comparing models with and without the relevant interaction terms for potassium with MRA use.

*****Models were stratified according to the trial and with the treatment assignment as covariate.

******Models were stratified according to the trial and with the treatment assignment as covariate, and adjusted for age, sex, region, race, heart rate, New York Heart Association functional classes III or IV, systolic blood pressure, body mass index, left ventricular ejection fraction, serum creatinine, hemoglobin, NT-proBNP (log-transformed), history of atrial fibrillation, history of myocardial infarction, history of diabetes, diuretics use, and angiotensin-converting enzyme inhibitor/angiotensin receptor blocker/angiotensin receptor neprilysin inhibitor use.

In the cases with missing data, the missing indicator method was used for NT-proBNP values.

**Supplemental Table 12**. **Serious Adverse Events Related to Hyperkalaemia and Hypokalaemia Across Trials**

|  | Hyperkalemia | | | | Hypokalemia | | | |
| --- | --- | --- | --- | --- | --- | --- | --- | --- |
|  | Any SAE (including death) | | Fatal SAE | | Any SAE (including death) | | Fatal SAE | |
| Trial | Control | Intervention | Control | Intervention | Control | Intervention | Control | Intervention |
| CHARM-Added/Alternative | 37/2287 (1.6) | 59/2289 (2.6) | 1/2287 (0.04) | 2/2289 (0.09) | 19/2287 (0.8) | 10/2289 (0.4) | 0/1509 (0) | 0/1514 (0) |
| EMPHASIS-HF | 7/1372 (0.5) | 19/1364 (1.4) | 0/1372 (0) | 0/1364 (0) | 6/1372 (0.4) | 4/1364 (0.3) | 0/1372 (0) | 0/1364 (0) |
| ATMOSPHERE (1)* | 20/2345 (0.9) | 13/2348 (0.5) | 0/2345 (0) | 1/2348 (0.04) | 10/2345 (0.4) | 12/2348 (0.5) | 0/2345 (0) | 0/2345 (0) |
| ATMOSPHERE (2)* | 20/2345 (0.9) | 21/2347 (0.9) | 0/2345 (0) | 2/2347 (0.09) | 10/2345 (0.4) | 10/2347 (0.4) | 0/2345 (0) | 0/2345 (0) |
| PARADIGM-HF | 42/4210 (1.0) | 17/4185 (0.4) | 3/4210 (0.07) | 1/4185 (0.02) | 9/4210 (0.2) | 12/4185 (0.3) | 0/4210 (0) | 1/4185 (0.02) |
| DAPA-HF | 5/2368 (0.2) | 2/2368 (0.1) | 0/2368 (0) | 0/2368 (0) | 0/2368 (0) | 2/2368 (0.1) | 0/2368 (0) | 0/2368 (0) |
| GALACTIC-HF | 21/4101 (0.5) | 20/4110 (0.5) | 0/4101 (0) | 1/4110 (0.02) | 13/4101 (0.3) | 7/4110 (0.2) | 0/4101 (0) | 0/4110 (0) |
| CHARM-Preserved | 9/1509 (0.6) | 24/1514 (1.6) | 0/1509 (0) | 0/1514 (0) | 16/1509 (1.1) | 5/1514 (0.3) | 0/1509 (0) | 0/1514 (0) |
| I-Preserve | 9/2061 (0.4) | 9/2067 (0.4) | 0/2061 (0) | 0/2067 (0) | 11/2061 (0.5) | 2/2067 (0.1) | 0/2061 (0) | 0/2067 (0) |
| TOPCAT (Americas  ) | 5/881 (0.6) | 19/886 (2.1) | 0/881 (0) | 1/886 (0.1) | 4/881 (0.5) | 0/886 (0) | 0/881 (0) | 0/886 (0) |
| PARAGON-HF | 42/2388 (1.8) | 19/2407 (0.8) | 0/2388 (0) | 2/2407 (0.08) | 14/2388 (0.6) | 8/2407 (0.3) | 1/2388 (0.04) | 0/2407 (0) |
| FINEARTS-HF | 6/2993 (0.2) | 16/2993 (0.5) | 0/2993 (0) | 0/2993 (0) | 6/2993 (0.2) | 3/2993 (0.1) | 0/2993 (0) | 0/2993 (0) |
| Total** | 203/26515 (0.8) | 238/28878 (0.8) | 4/26515 (0.02) | 10/28878 (0.03) | 108/26515 (0.4) | 75/28878 (0.3) | 1/26515  (0.004) | 1/28878  (0.003) |

Values are expressed as n/N (%). Abbreviations: SAE, serious adverse event

* (1) enalapril vs aliskiren; (2) enalapril vs combination of aliskiren plus enalapril

** In the control group, enalapril from the ATMOSPHERE trial was counted only once.

SAEs related to hyperkalaemia and hypokalaemia were reviewed by using the original publications, clinical study reports, and additional analyses. We subsequently report hyperkalaemia- and hypokalaemia-related SAEs and fatal SAEs, separately for the intervention and control groups in each trial.

It should be noted that these analyses were performed in the overall trial population or the safety population within each trial, and therefore include patients who were excluded from the present study. Unless otherwise specified, SAEs were identified according to the definitions used in each individual trial.

CHARM-Added, Alternative, and Preserved were checked by using clinical study reports.

EMPHASIS-HF was checked by using the following URL and analysing original data. (<https://clinicaltrials.gov/study/NCT00232180?term=EMPHASIS-HF&rank=1&tab=results>) accessed on 20^th^ Jan 2026.

ATMOSPHERE was checked by using clinical study reports.

PARADIGM-HF was checked using by the following URL and analysing original data. (<https://www.accessdata.fda.gov/drugsatfda_docs/nda/2015/207620orig1s000medr.pdf>) accessed on 20^th^ Jan 2026. A fatal SAE was defined as an SAE for which the end date coincided with the date of death.

DAPA-HF was checked by using Supplementary Appendix of the original paper (N Engl J Med 2019;381:1995-2008. DOI: 10.1056/NEJMoa1911303) and analysing original data.

GALACTIC-HF, I-Preserve, and TOPCAT were checked by analysing original data.

I-Preserve was checked by analysing original data.

PARAGON-HF was checked by using Supplementary Appendix of the original paper (N Engl J Med 2019;381:1609-1620. DOI: 10.1056/NEJMoa1908655) and analysing original data.

FINEARTS-HF was checked by using the original paper (N Engl J Med 2024;391:1475-1485. DOI: 10.1056/NEJMoa2407107) and analysing original data. SAE was defined as fatal outcome or hospitalization due to hyperkalaema or hypokalaemia.

**Supplemental Figure 1. Inclusion and exclusion criteria of this analysis**

Abbreviations: HFpEF, heart failure with preserved ejection fraction; HFrEF, heart failure with reduced ejection fraction; K, potassium; LVEF, left ventricular ejection fraction

**Supplemental Figure 2. Distribution of baseline potassium levels in HFrEF and HFpEF**

Abbreviations: HF, heart failure; HFpEF, heart failure with preserved ejection fraction; HFrEF, heart failure with reduced ejection fraction.

**Supplemental Figure 3. Incidence rate for each outcome across serum potassium levels stratified by eGFR in HFrEF.**

(A) All-cause death, (B) CV death, (C) Sudden death, (D) Pump failure death, (E) Non-CV death, (F) First HF hospitalization, (G) First HF hospitalization or all-cause death, and (H) First HF hospitalization or CV death. The X axis shows potassium, and the Y axis shows the unadjusted incidence rate for each outcome. The solid line shows a continuous incidence rate, and the interrupted lines on either side of this show the 95% CI. All the incidence rates are shown per 100 patient-years. Abbreviations: eGFR, estimated glomerular filtration rate (mL/min/1.73m^2^); CV, cardiovascular; HF, heart failure; and HFrEF, heart failure with reduced ejection fraction.

**Supplemental Figure 4. Incidence rate of each outcome across serum potassium levels stratified by eGFR in HFpEF.**

(A) All-cause death, (B) CV death, (C) Sudden death, (D) Pump failure death, (E) Non-CV death, (F) First HF hospitalization, (G) First HF hospitalization or all-cause death, and (H) First HF hospitalization or CV death. The X axis shows potassium, and the Y axis shows the unadjusted incidence rate for each outcome. The solid line shows a continuous incidence rate, and the interrupted lines on either side of this show the 95% CI. All the incidence rates are shown per 100 patient-years. Abbreviations: eGFR, estimated glomerular filtration rate (mL/min/1.73m^2^); CV, cardiovascular; HF, heart failure; and HFpEF, heart failure with preserved ejection fraction.

**Supplemental Figure 5. Incidence rate of each outcome across serum potassium levels stratified by MRA use**

**in HFrEF.**

(A) All-cause death, (B) CV death, (C) Sudden death, (D) Pump failure death, (E) Non-CV death, (F) First HF hospitalization, (G) First HF hospitalization or all-cause death, and (H) First HF hospitalization or CV death. The X axis shows potassium, and the Y axis shows the unadjusted incidence rate for each outcome. The solid line shows a continuous incidence rate, and the interrupted lines on either side of this show the 95% CI. All the incidence rates are shown per 100 patient-years. Abbreviations: CV, cardiovascular; HF, heart failure; and HFpEF, heart failure with preserved ejection fraction; MRA, mineralocorticoid receptor antagonist.

**Supplemental Figure 6. Kaplan-Meier curves for clinical outcomes based on potassium level at baseline in HFrEF**

Kaplan-Meier curves illustrating the association between baseline potassium levels and the clinical outcomes listed below in HFrEF population.

(A) All-cause death, (B) CV death, (C) Sudden death, (D) Pump failure death, (E) Non-CV death, (F) First HF hospitalization, (G) First HF hospitalization or all-cause death, and (H) First HF hospitalization or CV death.

Abbreviations: CV, cardiovascular; HF, heart failure; HFrEF, heart failure with reduced ejection fraction.

**Supplemental Figure 7. Kaplan-Meier curves for clinical outcomes based on potassium level at baseline in HFpEF**

Kaplan-Meier curves illustrating the association between baseline potassium levels and the clinical outcomes listed below in HFpEF population.

(A) All-cause death, (B) CV death, (C) Sudden death, (D) Pump failure death, (E) Non-CV death, (F) First HF hospitalization, (G) First HF hospitalization or all-cause death, and (H) First HF hospitalization or CV death.

Abbreviations: CV, cardiovascular; HF, heart failure; HFpEF, heart failure with preserved ejection fraction.

**Supplemental Figure 8. Hazard ratios for each clinical outcome according to baseline potassium level in HFrEF.**

(A) All-cause death, (B) CV death, (C) Sudden death, (D) Pump failure death, (E) Non-CV death, (F) First HF hospitalization, (G) First HF hospitalization or all-cause death, and (H) First HF hospitalization or CV death.

Abbreviations: CV, cardiovascular; HF, heart failure; HFrEF, heart failure with reduced ejection fraction.

Unadjusted models were stratified according to the trial and with the treatment assignment as covariate.

Adjusted models were stratified according to the trial and with the treatment assignment as covariate, and adjusted for age, sex, region, race, heart rate, New York Heart Association functional classes III or IV, systolic blood pressure, body mass index, left ventricular ejection fraction, serum creatinine, hemoglobin, NT-proBNP (log-transformed), history of atrial fibrillation, history of myocardial infarction, history of diabetes, diuretics use, mineralocorticoid receptor antagonist use, and angiotensin-converting enzyme inhibitor/angiotensin receptor blocker/angiotensin receptor neprilysin inhibitor use. In the cases with missing data, the missing indicator method was used for NT-proBNP values.

**Supplemental Figure 9. Hazard ratios for each clinical outcome according to baseline potassium level in HFrEF (GALACTIC-HF, DAPA-HF, and PARADIGM-HF) adjusted by the model including dose of oral loop diuretics**

(A) All-cause death, (B) CV death, (C) Sudden death, (D) Pump failure death, (E) Non-CV death, (F) First HF hospitalization, (G) First HF hospitalization or all-cause death, and (H) First HF hospitalization or CV death.

Abbreviations: CV, cardiovascular; HF, heart failure; HFpEF, heart failure with preserved ejection fraction.

Unadjusted models were stratified according to the trial and with the treatment assignment as covariate.

Adjusted models were stratified according to the trial and with the treatment assignment as covariate, and adjusted for age, sex, region, race, heart rate, New York Heart Association functional classes III or IV, systolic blood pressure, body mass index, left ventricular ejection fraction, serum creatinine, hemoglobin, NT-proBNP (log-transformed), history of atrial fibrillation, history of myocardial infarction, history of diabetes, diuretics use, mineralocorticoid receptor antagonist use, angiotensin-converting enzyme inhibitor/angiotensin receptor blocker/angiotensin receptor neprilysin inhibitor use, and dose of oral loop diuretics. In the cases with missing data, the missing indicator method was used for NT-proBNP values and with (red) and without (cyan) dose of oral loop diuretics.

**Supplemental Figure 10. Hazard ratios for each clinical outcome according to baseline potassium level in HFpEF.**

(A) All-cause death, (B) CV death, (C) Sudden death, (D) Pump failure death, (E) Non-CV death, (F) First HF hospitalization, (G) First HF hospitalization or all-cause death, and (H) First HF hospitalization or CV death.

Abbreviations: CV, cardiovascular; HF, heart failure; HFpEF, heart failure with preserved ejection fraction.

Unadjusted models were stratified according to the trial and with the treatment assignment as covariate.

Adjusted models were stratified according to the trial and with the treatment assignment as covariate, and adjusted for age, sex, region, race, heart rate, New York Heart Association functional classes III or IV, systolic blood pressure, body mass index, left ventricular ejection fraction, serum creatinine, hemoglobin, NT-proBNP (log-transformed), history of atrial fibrillation, history of myocardial infarction, history of diabetes, diuretics use, mineralocorticoid receptor antagonist use, and angiotensin-converting enzyme inhibitor/angiotensin receptor blocker/angiotensin receptor neprilysin inhibitor use. In the cases with missing data, the missing indicator method was used for NT-proBNP values.

**Supplemental Figure 11. Hazard ratios for each clinical outcome according to baseline potassium level in HFpEF (FINEARTS-HF, and PARAGON-HF) adjusted by the model including dose of oral loop diuretics**

(A) All-cause death, (B) CV death, (C) Sudden death, (D) Pump failure death, (E) Non-CV death, (F) First HF hospitalization, (G) First HF hospitalization or all-cause death, and (H) First HF hospitalization or CV death.

Abbreviations: CV, cardiovascular; HF, heart failure; HFpEF, heart failure with preserved ejection fraction.

Unadjusted models were stratified according to the trial and with the treatment assignment as covariate.

Adjusted models were stratified according to the trial and with the treatment assignment as covariate, and adjusted for age, sex, region, race, heart rate, New York Heart Association functional classes III or IV, systolic blood pressure, body mass index, left ventricular ejection fraction, serum creatinine, hemoglobin, NT-proBNP (log-transformed), history of atrial fibrillation, history of myocardial infarction, history of diabetes, diuretics use, mineralocorticoid receptor antagonist use, angiotensin-converting enzyme inhibitor/angiotensin receptor blocker/angiotensin receptor neprilysin inhibitor use, and with (red) and without (cyan) dose of oral loop diuretics. In the cases with missing data, the missing indicator method was used for NT-proBNP values and dose of oral loop diuretics.
